# Energy requirements for pregnant dairy cows

**DOI:** 10.1371/journal.pone.0235619

**Published:** 2020-07-07

**Authors:** Anna Luiza Lacerda Sguizzato, Marcos Inácio Marcondes, Jan Dijkstra, Sebastião de Campos Valadares Filho, Mariana Magalhães Campos, Fernanda Samarini Machado, Breno Castro Silva, Polyana Pizzi Rotta

**Affiliations:** 1 Department of Animal Science, Universidade Federal de Viçosa, Viçosa, Minas Gerais, Brazil; 2 Animal Nutrition Group, Wageningen University and Research, Wageningen, The Netherlands; 3 Embrapa Gado de Leite, Juiz de Fora, Minas Gerais, Brazil; Michigan State University, UNITED STATES

## Abstract

This study aimed to estimate energy requirements of pregnant Holstein × Gyr cows. Different planes of nutrition were established by two feeding regimens: ad libitum or maintenance. Sixty-two nonlactating cows with average body weight of 480 ± 10.1 kg and an age of 5 ± 0.5 years were used. Cows were divided into three groups: pregnant (n = 44), non-pregnant (n = 12), and baseline reference (n = 6). The 56 pregnant and non-pregnant cows were randomly allocated into a feeding regimen: ad libitum or maintenance. To evaluate the effects of days of pregnancy, pregnant and non-pregnant animals were slaughtered at 140, 200, 240, and 270 days of pregnancy. Energy requirements for maintenance differed between pregnant and non-pregnant cows, thus two equations were developed. Net energy and metabolizable energy requirements for maintenance of non-pregnant cows were 82 kcal/kg empty body weight^0.75^/day and 132 kcal/kg empty body weight^0.75^/day, respectively. The efficiency of use of metabolizable energy for maintenance of non-pregnant cows was 62.4%. Net energy and metabolizable energy for maintenance of pregnant cows were 86 kcal/kg empty body weight^0.75^/day and 137 kcal/kg empty body weight^0.75^/day, respectively. Efficiency of use of metabolizable energy for maintenance of pregnant cows was 62.5%. The efficiency of use of metabolizable energy for gain was 41.9%. The efficiency of use of metabolizable energy for pregnancy was 14.1%. Furthermore, net energy requirement for pregnancy was different from zero from day 70 of pregnancy onwards. In conclusion, net energy and metabolizable energy requirements for maintenance of non-pregnant cows are different from pregnant cows. Furthermore, we believe that the proposed non-linear equations to estimate net energy requirements for pregnancy are more adequate than current NRC equation, and should be recommended for Holstein × Gyr cows.

## Introduction

In dairy cattle, the late pregnancy period is important to prepare the mammary gland for lactation. This is also a period of utmost importance for fetal development [1]. Nevertheless, there are few studies to estimate energy requirements during pregnancy [1, 2, 3, 4]. Among available requirement systems [5, 6, 7, 8], the NRC [6] is the most widely used for dairy cattle.

Energy requirements for pregnancy in the NRC [6] were established according to results obtained by Bell et al. [3] and the efficiency of use of metabolizable energy by the conceptus was estimated by Ferrell et al. [1]. According to Bell et al. [3], the energy required for pregnancy is significant from 190 days, with linear accretion until 279 days. These pregnancy requirements were obtained with cows from *Bos taurus* breeds. Thus, when evaluating requirements for animals from different breeds, such as crossbred *Bos taurus* × *Bos indicus* (Holstein × Gyr), there is no clear evidence that energy requirements for pregnancy would be the same.

The Brazilian dairy herd is composed of approximately 70% of Holstein × Gyr animals [9, 10] and this crossbred has greater milk production than the Gyr breed itself. This greater milk production is likely a result of heterosis, which incorporates the best characteristics of each breed; milk production from Holstein and adaptability to tropical climate from Gyr [10]. In a meta-analysis, Oliveira [11] found lower maintenance requirements and lower efficiency for milk production of crossbred *Bos taurus* × *Bos indicus*, compared to *Bos taurus* breeds. Nevertheless, to our knowledge, no quantitative data are available on nutrient requirements of pregnant Holstein × Gyr cows. In addition, no studies were found regarding requirements for gain of Holstein × Gyr cows.

Therefore, studies evaluating if nutrient requirements for pregnant Holstein × Gyr cows differ from requirements for *Bos taurus* breeds are warranted. Thus, we hypothesized that energy requirements estimated for pregnant crossbred Holstein × Gyr cows might differ from what is predicted for *Bos taurus* breeds based on Bell et al. [3] and on metabolizable energy efficiency of gravid uterus of Ferrel et al. [1]. For this reason, the aim of this study was to estimate energy requirements of maintenance, pregnancy, and gain of pregnant Holstein × Gyr cows.

## Material and methods

### Animals and management

This study was carried out in strict accordance with the law no. 11.794, of October 8, 2008, Decree no. 6899 of July 15, 2009, and the rules issued by the Brazilian National Council for Animal Experimentation Control (CONCEA), and was approved by the Ethics Commission on the use of farm animals of Universidade Federal de Viçosa (CEUAP-UFV), protocol number: 47/2012.

**Fig 1:**
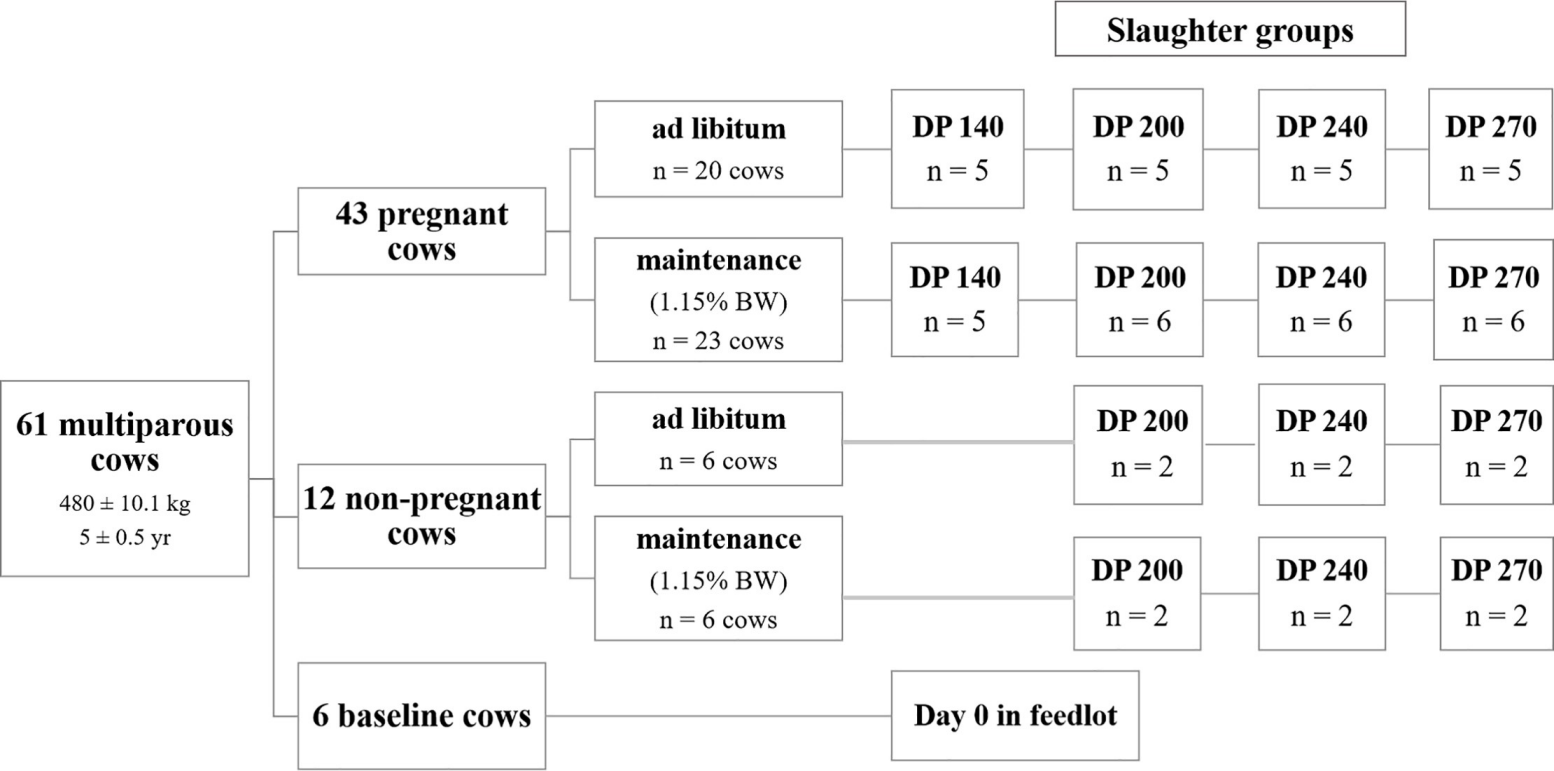
Experimental scheme, feeding regimens and slaughter groups.

Data used in the present analysis were obtained from a previous study conducted by Rotta et al. [12, 13, 14]. Briefly, sixty-two Holstein × Gyr cows with an average initial weight of 480 ± 10.1 kg and 5 ± 0.5 years were used. They were divided into 3 groups: pregnant (n = 44), non-pregnant (n = 12) and baseline (n = 6). Firstly, all 62 cows (all in non-pregnant stage) underwent an adaptation period of 14 days. The adaptation period is necessary to standardize all animals and management conditions. After adaptation, all baseline cows were slaughtered to compose the reference group, which is essential in comparative slaughter trials, once their body composition was used to estimate initial body composition and initial body weight of the remaining cows. After the slaughter of baseline cows, two different feeding regimens, ad libitum or maintenance (1.15% of body weight), were distributed among the remaining 56 cows, pregnant (44) and non-pregnant (12). One abortion was verified in a cow from the maintenance treatment at 140 days. Thus, data from 43 pregnant cows were used for analyses, and 5 cows at maintenance level were evaluated at 140 days of pregnancy. These 43 Holstein × Gyr cows were slaughtered at four different days of pregnancy: 140, 200, 240, and 270 days. The 12 remaining cows (non-pregnant cows) were slaughtered at 200, 240 and 270 days in feedlot (Fig 1).

Cows were housed in 30 m^2^ individual pens, of which 8 m^2^ was covered with concrete floors and equipped with individual feed bunks and an automatic water system. They were fed corn silage and a concentrate-based diet at a ratio of 93:7 on a dry matter basis as a total mixed ratio twice daily. The amounts of corn silage, concentrate, and orts were recorded daily. All cows had ad libitum access to water and in order to allow ad libitum access to feed, its delivery was adjusted to approximately 5% orts daily on an as-fed basis.

### Slaughtering procedures and sampling

**Fig 2:**
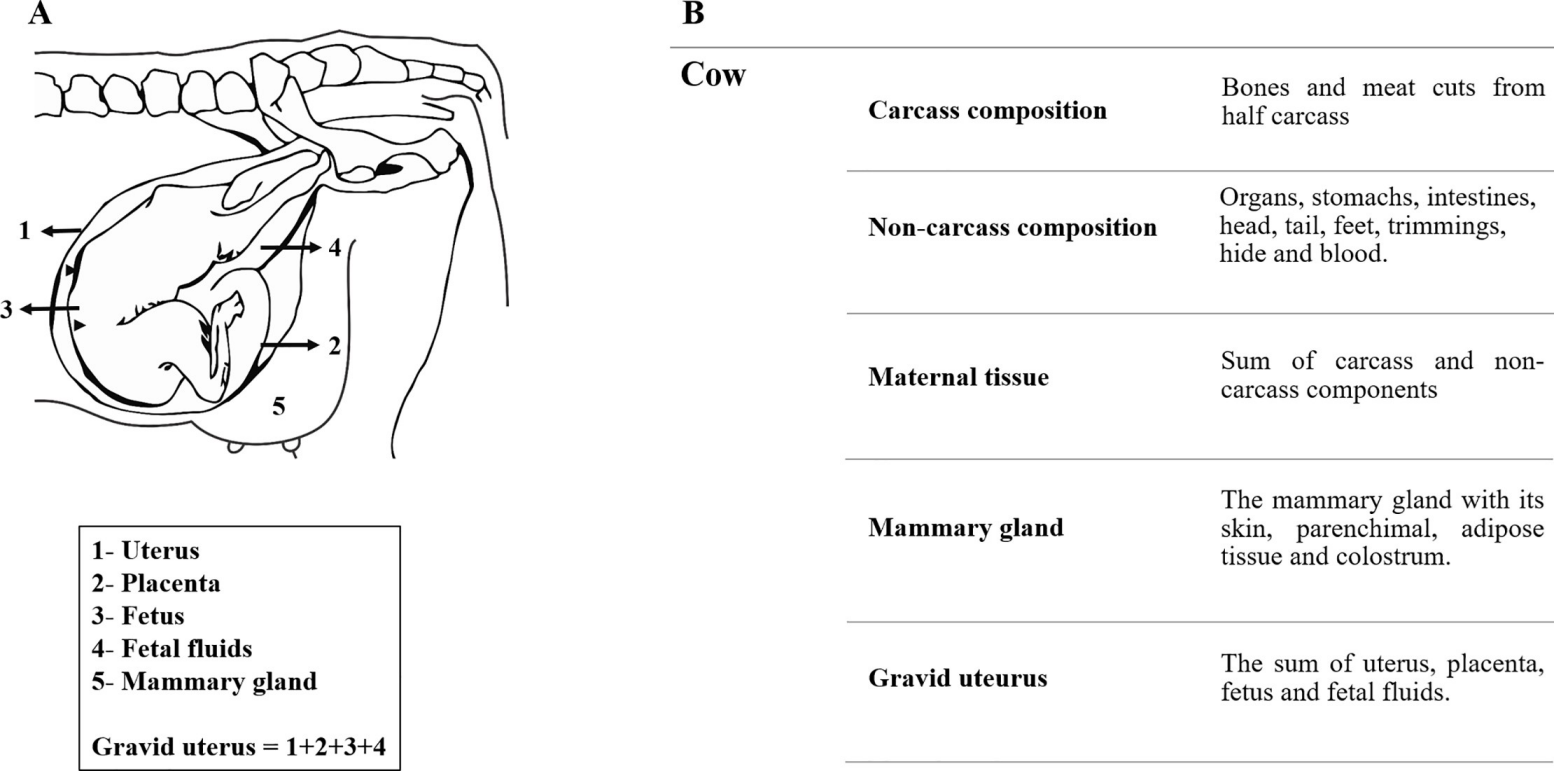
Pregnant and non-pregnant cow’s components. Components of gravid uterus and mammary gland (A) and maternal tissues’ sub-divisions according to sampling at slaughter (B).

Cows were slaughtered by a captive bolt stunner, followed by exsanguination. Right after exsanguination, the gravid uterus was immediately collected and weighted. It was sectioned in cervix height and then dissected in fetus, fetal membranes, uterus and fetal fluids, in a way that the weight of fetal fluids was obtained by difference between pregnant uterus and the other sectioned parts. Fetus, fetal membranes, and uterus were ground and sampled individually. The mammary gland was also sectioned and entirely ground (Fig 2). Then, a homogenized sample was created with uterus and fetal membranes. Samples from gravid uterus and mammary gland were maintained at -80ºC until further chemical analyses.

The carcass of each animal was divided into two half carcasses. They were weighted to determine carcass hot yield, then allocated in a cold chamber at 4ºC, during 24 h. Posteriorly, the carcasses were weighted to determine cold carcass yield. In addition, to compose the non-carcass sample, the four chambers of the stomach, small and large intestines were washed after slaughter and added to internal organs, head, tail, feet, trimmings, hide, and blood. All components were ground and homogenized, and then a sample of each carcass and non-carcass was taken for further analyses and finally compose cow’s tissue sample (Fig 2).

There were six periods of spot fecal collections, for evaluation of apparent total-tract digestibility, with each period lasting 28 days. Feces from all cows were collected during the last 5 days of each 28 days of period. Fecal collections were performed at 0600, 0900, 1200, 1500, and 1800 h on days 1, 2, 3, 4, and 5, respectively. A composite sample was obtained per collection period for each cow by utilizing 15 g of the dried and ground sample per collection time [12, 13, 14].

### Laboratory analyses

Samples of carcass, non-carcass, mammary gland, uterus, placenta, fetal fluids, fetus and feces were analyzed for dry matter [15; method 934.01], crude protein [15; method 981.10], and ether extract [16; method 945.16]. Energy content was estimated based on protein and ether extract contents, as proposed by ARC [17].

Fecal dry matter excretion was estimated by the internal marker technique [18], where indigestible neutral detergent fiber was the internal marker. Indigestible neutral detergent fiber content from feces, feeds, and orts were quantified in triplicate and obtained by in situ incubation procedures. The bags were incubated for 288 h [19] in the rumen of 2 cannulated bulls fed a diet consisting of 50% corn silage and 50% concentrate on a dry matter basis at maintenance level. More details about management and laboratory analyses are available in Rotta et al. [12, 13, 14].

### Calculations and estimates

Dietary non-fiber carbohydrates content was calculated according to Detmann and Valadares Filho [20]:

%Non‐fibercarbohydrates=100‐[(%crudeprotein‐%crudeproteinurea+%urea)+%neutraldetergentfiber+%etherextract+%crudeash] (1)

Where all variables are in % of diet DM.

The digestible energy of the diet was obtained by multiplying the digestible fraction of each nutrient by its caloric value [6]:

Digestibleenergy=(5.6×digestiblecrudeprotein)+(9.4×digestibleetherextract)+(4.2×digestibleneutraldetergentfiber)+(4.2×digestiblenon‐fibercarbohydrates) (2)

Where digestible energy = Mcal/day and digestible crude protein, digestible ether extract, digestible neutral detergent fiber, and digestible non-fiber carbohydrates = kg/day.

Metabolizable energy was obtained by the equation from NRC [21]:

Metabolizableenergy(Mcal/kgofdrymatter)=digestibleenergy(Mcal/kgofdrymatter)×0.82 (3)

Empty body weight of cows was composed by carcass, non-carcass, mammary gland and uterus. For pregnant cows, to estimate uterus and mammary gland components exclusively due to pregnancy, uterus and mammary gland components were estimated as if they were not pregnant. The empty body weight energy content for both non-pregnant and pregnant cows were obtained from the body contents of protein and fat and their respective caloric equivalents of 5.7 and 9.5 Mcal/kg [17].

Heat production was calculated as the difference between metabolizable energy intake and retained energy, which was calculated as the difference between the initial and the final total body energy content. The net energy requirement for maintenance (NE_m_) was assumed to be the intercept (β0) of the exponential regression between metabolizable energy intake and heat production (Eq 4), as proposed by Ferrell and Jenkins [22].

$$Heat\;production=\beta_{0}\times e^{\left(\beta 1\times metabolizable\;energy\;intake\right)}$$ (4)

Where: heat production and metabolizable energy intake are expressed in Mcal/kg empty body weight^0.75^/day, and *β*_0_ and *β*_1_ are the equation parameters.

For all comparisons between non-pregnant and pregnant animals, and among pregnancy groups, for the purpose of statistical tests, animals slaughtered between 137 and 144 days of pregnancy were included in the model as 140 days. Animals slaughtered between 196 and 201 days were included in the model as 200 days, animals slaughtered between 236 and 247 days were included in the model as 240 days. Lastly, animals slaughtered between 266 and 270 days of pregnancy were included in the model as 270 days. In order to estimate NE_m_ for non-pregnant and pregnant cows separately, we fitted two models. The first model was estimated using non-pregnant cows only, and the second one was estimated using pregnant cows at 140, 200, 240 and 270 days. The effect of days of pregnancy was tested on both parameters of Eq 4.

Metabolizable energy requirements for maintenance (ME_m_) expressed as Mcal/kg empty body weight^0.75^/day were estimated by the iterative method, as the point where metabolizable energy intake equals heat production (i.e., the point at which there is no energy retention in the body). In addition, the efficiency of use of metabolizable energy for maintenance (k_m_) was estimated by the ratio between net energy and ME_m_. Separate estimates of ME_m_ for non-pregnant and pregnant cows were obtained.

Requirements of net energy for gain (NE_g_) of non-pregnant animals were estimated as retained energy according to the equation proposed by Garret [23]:

$$Net\;energy\;for\;gain=\beta_{0}\times empty\;body\;weight^{0.75}\times empty\;body\;gain^{\beta 1}$$ (5)

Where: net energy for gain = Mcal/day, empty body weight = kg, empty body gain = kg/day and *β*_0_ and *β*_1_ are equation parameters.

Requirements of NEg in pregnant cows were considered as the amount of retained energy in maternal tissue (which consists of empty body weight minus the gravid uterus minus the mammary gland) of pregnant animals. Retained energy in maternal tissue of pregnant cows was estimated by a non-linear regression in function of maternal tissue composition, where only carcass and non-carcass weight were considered (Fig 2) and gain of maternal tissue (kg/day; Eq 6). Requirements for non-maternal tissues (mammary gland and gravid uterus) in pregnant cows were calculated separately.

$$Retained\;energy\;in\;maternal\;tissue=\beta_{0}\times maternal\;tissue^{0.75}\times gain\;of\;maternal\;tissue^{\beta 1}$$ (6)

Where: retained energy in maternal tissue of pregnant cows = Mcal/day, maternal tissue^0.75^ = kg, gain of maternal tissue = kg/day and *β*_0_ and *β*_1_ are equation parameters.

Average maternal tissue was considered the average of initial maternal tissue and final maternal tissue. A linear regression considering maternal tissue from animals of the baseline group in function of empty body weight was used to estimate initial maternal tissue.

To estimate energy requirements for pregnancy, the balance of pregnancy components and days of pregnancy was considered. Balance of pregnancy components was calculated as the sum of the difference between final and initial energy content of the gravid uterus and the difference between final and initial energy content of the udder in Mcal. To predict initial pregnancy components, a linear regression was estimated in function of final pregnancy components and final empty body weight, with baseline group and non-pregnant cows.

Three initial models were selected to fit pregnancy components weight and composition in function of days of pregnancy: one linear and two non-linear models (quadratic, simple exponential, and double exponential, respectively; Eqs 7, 8 and 9).

$$Quadratic\;model=\beta_{0}+\beta_{1}\times days\;of\;pregnancy+\beta_{2}\times days\;of\;pregnancy^{2}$$ (7)

$$Simple\;exponential\;model=\beta_{0}\times exp^{(\beta }{{}_{1}}^{\times days\;of\;pregnancy)}$$ (8)

$$Double\;exponential\;model=\beta_{0}\times exp^{exp(\beta }{{}_{1}}^{\times days\;of\;pregnancy\;)}$$ (9)

To evaluate the best fit, the AIC was used and Eq 8 presented the lowest AIC (303.9). The first derivate of Eq 8 was assumed to be the net energy for pregnancy (NE_preg_). In addition, an adjustment in function of calf body weight was added to the model as proposed by NRC [6].

The efficiency of use of metabolizable energy for gain of non-pregnant cows (k_g_) was estimated according to Marcondes et al. [24], considering only metabolizable energy intake on maintenance. The k_g_ was assumed to be the slope (*β*_1_) of the regression of retained energy in function of metabolizable energy intake for gain (calculated as metabolizable energy intake—metabolizable energy intake for maintenance).

$$Retained\;energy=\beta_{0}+\beta_{1}\times metabolizable\;energy\;for\;gain$$ (10)

Where: retained energy = Mcal/kg empty body weight^0.75^/day, metabolizable energy intake for gain = Mcal/kg empty body weight^0.75^/day and *β*_0_ and *β*_1_ are equation parameters.

The efficiency of use of metabolizable energy for pregnancy was estimated by iterative method using Eq 11.

$$\Delta =MEI-\left(\frac{NE_{m}}{k_{m}}+\frac{RE_{MT}}{k_{g}}+\frac{PREG}{k_{preg}}\right)$$ (11)

Where: MEI = daily metabolizable energy intake, NE_m_ = daily net energy requirement for maintenance estimated in this study, RE_MT_ = daily retained energy in maternal tissue, PREG = daily retained energy in the gravid uterus and the udder, k_m_ = efficiency of use of metabolizable energy for maintenance estimated in this study, k_g_ = efficiency of use of metabolizable energy for gain and k_preg_ = efficiency of use of metabolizable energy for pregnancy. The iteration was performed aiming an average Δ of zero. The parameters NE_m_, k_m_, retained energy in maternal tissue of pregnant cows_,_ k_g_ and PREG were already calculated. Thus, we estimated only the k_preg_ by iteration.

Requirements of metabolizable energy for gain of pregnant and non-pregnant cows were estimated by the ratio between NE_g_ and k_g_. Requirements of metabolizable energy for pregnancy were estimated by the ratio between daily net energy requirement for pregnancy (NE_preg_) and k_preg_.

### Statistical analyses

The model used to estimate maintenance requirements was fit using PROC NLMIXED of SAS (version 9.4; SAS Institute, Inc. Cary, NC). The effect of physiological condition (pregnant and non-pregnant) was tested on both parameters, *β*_0_ and *β*_1_. Models to estimate requirements for gain were evaluated using PROC NLMIXED of SAS (version 9.4; SAS Institute, Inc. Cary, NC). The effect of physiological condition was also tested on *β*_0_ and *β*_1_. Regarding requirements for pregnancy, effects of feeding regimen were tested on both parameters *β*_0_ and *β*_1_ of Eq 8 using PROC NLMIXED of SAS (version 9.4; SAS Institute, Inc. Cary, NC).

For all statistical analyses, the student t test was used to compare feeding regimen, and all significances were declared when P < 0.05.

## Results and discussion

### Net and metabolizable energy requirements for maintenance

**Fig 3:**
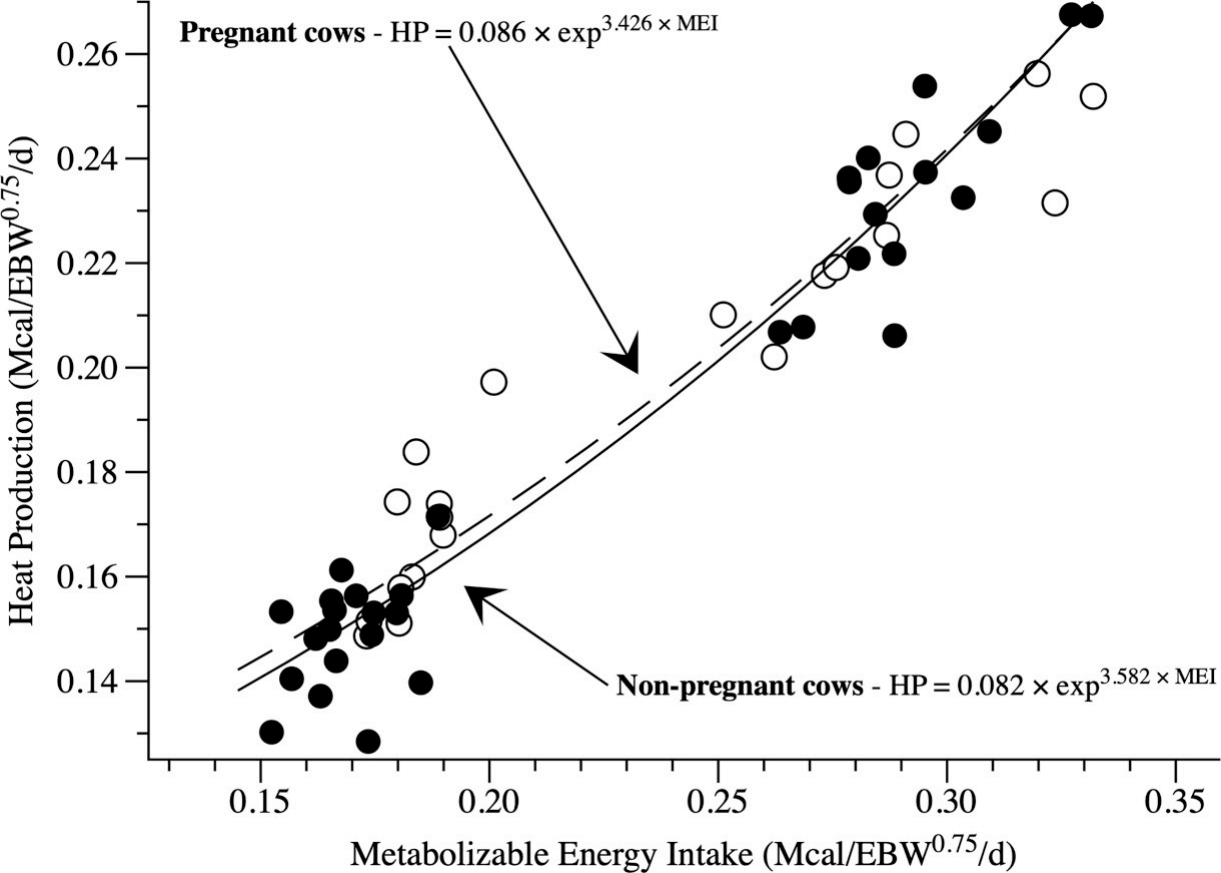
Representation of heat production equation for non-pregnant and pregnant cows. Close symbols refer to non-pregnant cows and open symbols refer to pregnant cows.

When evaluating the effect of days of pregnancy on the coefficients of the model to predict the NE_m_, we observed a difference between non-pregnant and pregnant cows on both parameters β_0_ and β_1_. The difference found between 0 and 140 days (*P* = 0.010 and *P* = 0.025, respectively) indicated that, during pregnancy, Holstein × Gyr cows may present a distinct NE_m_ (Fig 3).

No differences were observed from 140 to 270 days of pregnancy (*P* > 0.05). Thus, two equations were considered, one for each condition (non-pregnant, Eq 12; and pregnant, Eq 13).

$$Heat\;production_{non-pregnant}=0.0822\pm 0.0052\times e^{3.5822\pm 0.2574\times metabolizable\;energy\;intake}$$ (12)

(R^2^ = 0.9667; RMSE = 0.00004)

$$Heat\;production_{pregnant}=0.0857\pm 0.0036\times e^{3.4258\pm 0.1593\times metabolizable\;energy\;intake}$$ (13)

(R^2^ = 0.9186; RMSE = 0.0001)

Where: heat production and metabolizable energy intake are in Mcal/kg empty body weight^0.75^/day.

**Fig 4:**
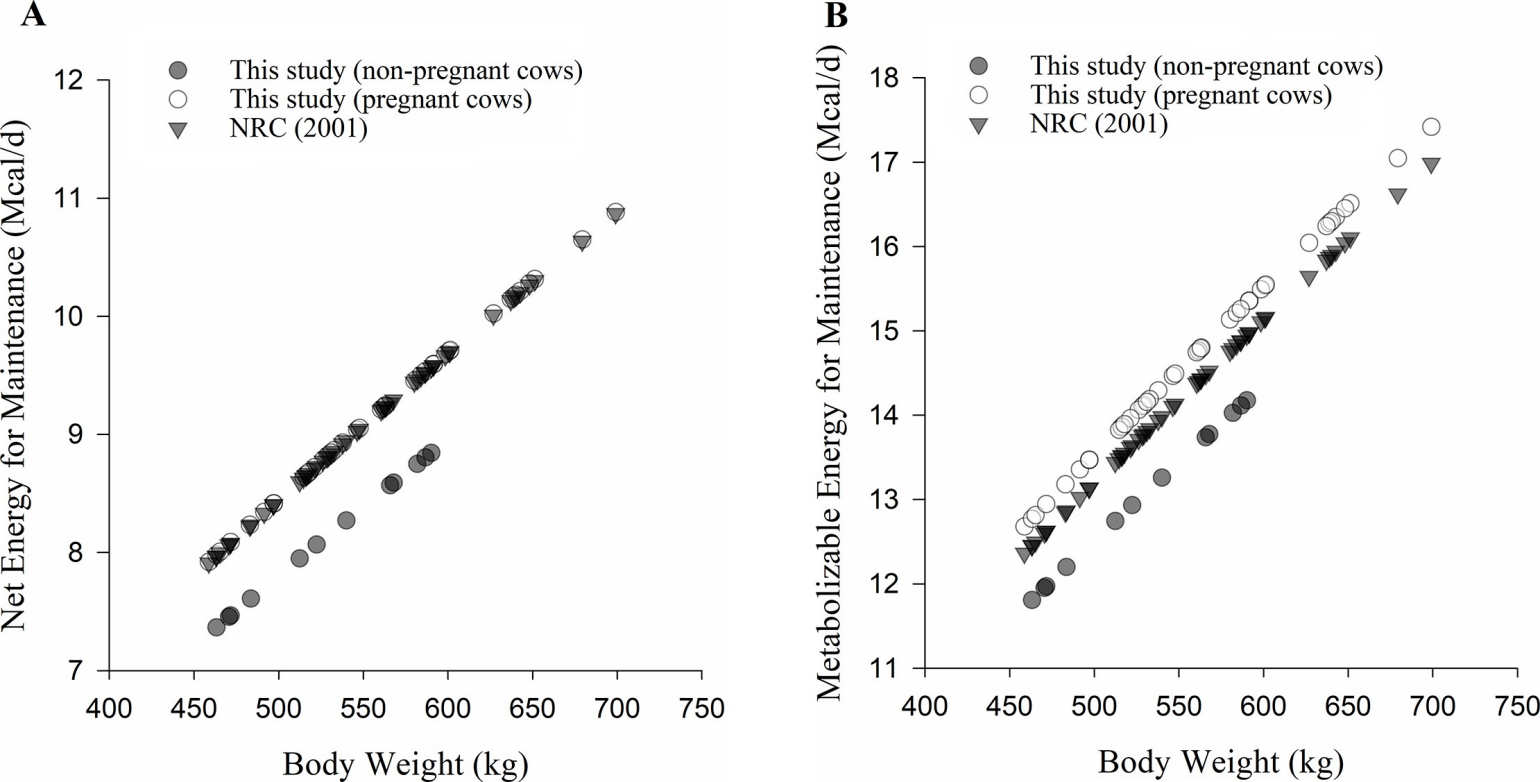
Net energy for maintenance. A—Estimation of net energy for maintenance requirement from NRC (2001) equation and the estimated equation from non-pregnant and pregnant Holstein × Gyr cows (this study). B–Estimation of metabolizable energy for maintenance requirement from NRC (2001) equation and the estimated equation from Holstein × Gyr cows (this study). Closed and open circles refer to values obtained from this study and closed triangles refer to values obtained from NRC (2001).

The estimated value of NE_m_ in this study for non-pregnant cows was 82 kcal/kg empty body weight^0.75^/day or 74 kcal/kg body weight^0.75^/day. Therefore, the value obtained is approximately 8% lower than the NE_m_ value from NRC [6], which was 80 kcal/kg body weight^0.75^/day (Fig 4A). The NE_m_ requirement for pregnant cows was 86 kcal/kg empty body weight^0.75^/day or 80 kcal/kg body weight^0.75^/day. This value, with approximations, is equal to the NE_m_ suggested by the NRC [6]. Lage [25] found values of 77 and 92 kcal/kg body weight^0.75^/day of NE_m_ for non-pregnant Gyr and crossbred Holstein × Gyr heifers, respectively. Estimations for energy requirements by Lage [25] were obtained by the indirect calorimetry technique. Furthermore, the BR-CORTE [4] considers NE_m_ for pregnant and non-pregnant Nellore cows of 86 kcal/kg empty body weight^0.75^/day, a value close to the estimate of pregnant cows in this study.

Metabolizable energy for maintenance was estimated by the iterative method, as the point where heat production equals metabolizable energy intake. The ME_m_ for non-pregnant cows was 132 ± 2.51 kcal/kg empty body weight^0.75^/day or 119 kcal/kg body weight^0.75^/day (Fig 4B). The efficiency of use of ME_m_ for non-pregnant cows was 62.4%. However, for pregnant cows, ME_m_ was greater than non-pregnant cows, 137 ± 1.73 kcal/kg empty body weight^0.75^/day or 127 kcal/kg body weight^0.75^/day. The efficiency of use of metabolizable energy for pregnant cows was 62.5%.

Solis et al. [26] compared energy requirements of five breeds (non-pregnant animals), three for beef production and two for milk production. In their study, they found a ME_m_ of 119 kcal/kg body weight^0.75^/day for Holstein cows. The NRC [6] considers a k_m_ of 64% and ME_m_ of 125 kcal/kg body weight^0.75^/day. Lage [25] also estimated ME_m_ for non-pregnant heifers and found greater values, 120 and 146 kcal/kg body weight^0.75^/day for Gyr and Holstein × Gyr crossbred, respectively. The BR-CORTE [4] considers as ME_m_ a value of 120 kcal/kg empty body weight^0.75^/day for non-pregnant and pregnant Nellore cows, with an increase of 8.5% in requirements for ME_m_ when animals are raised on pasture.

Our estimates of NE_m_ and ME_m_ for non-pregnant and pregnant cows presented a difference, on average, of 5%. However, for a greater metabolizable energy intake, heat production becomes closer for cows in both physiological states. Moreover, non-pregnant cows showed ME_m_ values close to those of Solis et al. [26] and Lage [25] (considering the Gyr heifers’ results from the last author). In contrast, pregnant cows had NE_m_ and ME_m_ equal to NRC [6] requirements, indicating similarity among these recommendations, regarding maintenance requirements.

Solis et al. [26] also found distinct differences for maintenance requirements among beef and milk breeds. The NASEM [27] and BR-CORTE [4] also suggest different requirements for *Bos taurus* and *Bos indicus* breeds. A possible explanation to these lower results obtained for non-pregnant cows may be related to the size of internal organs and amount of visceral fat [11, 22, 26], which may be smaller for crossbred Hostein × Gyr cows when compared to pure breed *Bos taurus* cows. Even though a small proportion of body weight is composed by internal organs, they contribute to high metabolic rates [22], accounting for 40 to 50% of energy requirements in ruminants [25], which results in an increase in heat production.

Furthermore, during pregnancy, the gravid uterus and the mammary gland accounts for a greater increase in heat production. Considering the increase in NE_m_ and ME_m_ for pregnant cows, it suggests an increase in heat production produced by the gravid uterus and/or developing mammary gland. Our calculations account exclusively the increase in gravid uterus and mammary gland due to pregnancy; and according to our results and observations, we speculate that there may be a greater increase in heat production caused by the gravid uterus than caused by the mammary gland, especially because the fetus uses a large quantity of amino acids as source of energy [28], which is linked to a greater heat production when compared with carbohydrates metabolism [29].

### Net and metabolizable energy for gain

Energy requirements for gain were estimated using data from non-pregnant (Eq 14) and pregnant cows (Eq 15).

$$Net\;energy\;for\;gain=0.0624\pm 0.0016\times empty\;body\;weight^{0.75}\times empty\;body\;gain^{0.7241\pm 0.0473}$$ (14)

(R^2^ = 0.9766; RMSE = 0.1404)

Where: net energy for gain = Mcal/day, empty body weight^0.75^ = kg, empty body gain = kg/day.

$$Retained\;energy\;in\;maternal\;tissue\;of\;pregnant\;cows=0.0600\pm 0.0022\times maternal\;tissue^{0.75}\times gain\;of\;maternal\;tissue^{0.6562\pm 0.0725}$$ (15)

(R^2^ = 0.879; RMSE = 0.8192)

Where: retained energy in maternal tissue of pregnant cows = Mcal/day, metabolic maternal tissue = kg, gain of maternal tissue = kg/day.

**Fig 5:**
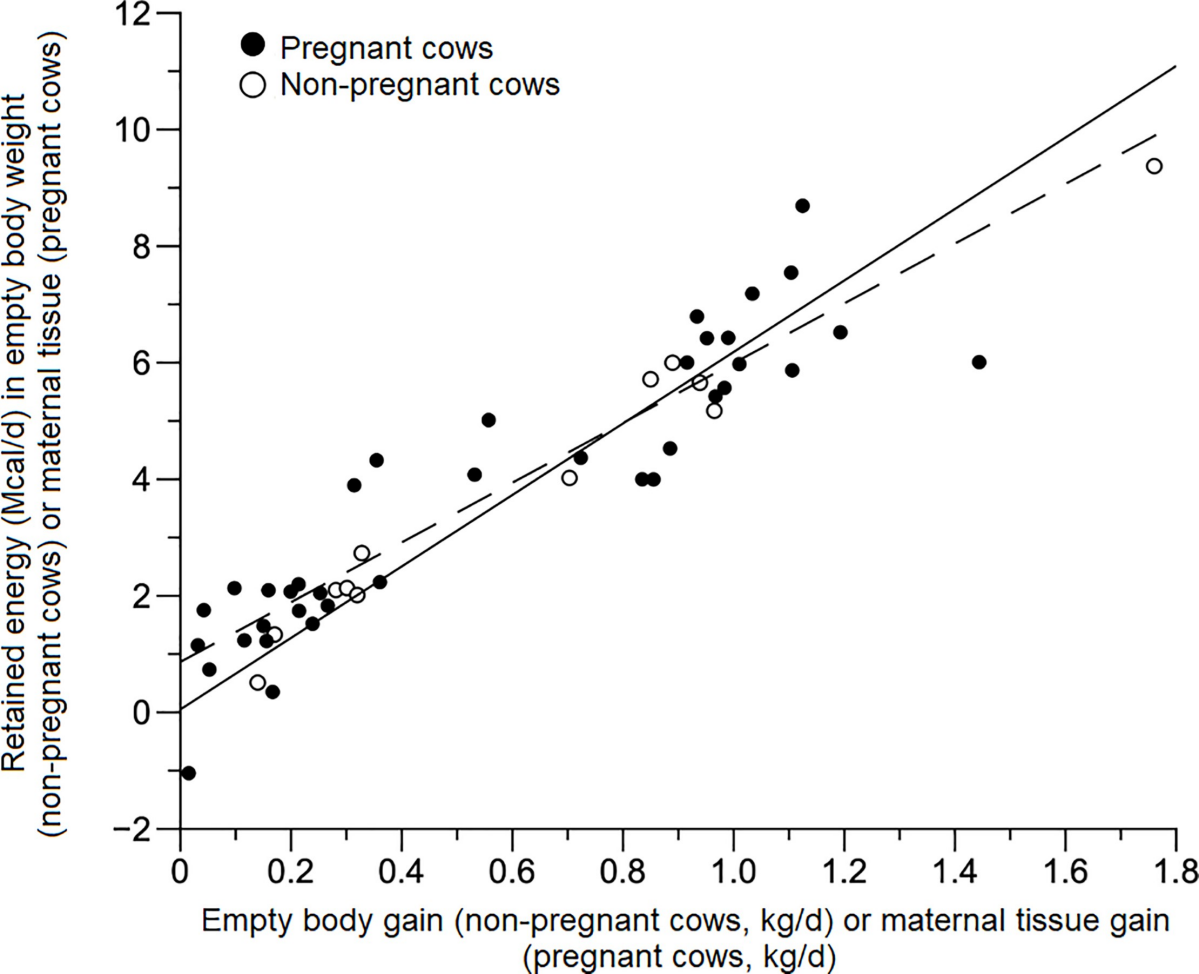
Retained energy in empty body weight or maternal tissue. Relation between retained energy in empty body weight (Mcal/day) for non-pregnant cows or in maternal tissue (Mcal/day) for pregnant cows and empty body gain (kg/day) for non-pregnant or maternal tissue gain (kg/day) for pregnant cows. Close circles refer to pregnant cows and open circles refer to non-pregnant cows.

The pattern of energy deposition in empty body gain and maternal tissue among non-pregnant and pregnant cows is similar (Fig 5).

The β_1_ parameter obtained in Eq 14 represents the variation on gain composition according to the amount of retained energy per day. Therefore, according to the estimated value in this study, for greater rates of gain (at the same body weight) of mature animals, a greater proportion of protein will be deposited, instead of energy as fat. The *β*_*1*_ coefficient of empty body gain (0.7241; Eq 14) for non-pregnant and pregnant cows (0.6562) (Eq 15) suggests a high proportion of protein in total gain for these animals. Moreover, cows from both physiological stages may have had greater rates of protein turnover, indicating the difference observed for the β_1_ parameter. Based on the *β*_*1*_ coefficient obtained for non-pregnant animals when compared to that obtained for pregnant cows (Eq 15) we suggest the use of Eq 14 for both pregnant and non-pregnant cows when estimating NE_g_.

We speculated that another explanation for this difference in the *β*_*1*_ parameter, especially for pregnant cows, would be related to the extensive use of amino acids by the fetus. According to Bell [28], the fetus uses a considerable amount of amino acids to meet growth requirements and for its own metabolism; however, the mechanism of how this uptake of nutrients may affect the composition of gain of pregnant mature cows is not known. Therefore, future studies should focus efforts on body composition changes during pregnancy to elucidate this hypothesis. Additionally, in this study, pregnancy did not affect crude protein or ether extract in both carcass and non-carcass components [30].

The efficiency of use of metabolizable energy for gain was estimated according to Marcondes et al. [24] (Eq 10), using only data from non-pregnant cows. The *β*_1_ was considered as the k_g_, which was 41.9%.

Retainedenergy=‐0.0558±0.0127+0.4189±0.0542×metabolizableenergyintakeforgain (16)

(R^2^ = 0.869; RMSE = 0.0001)

Where: retained energy = Mcal/kg empty body weight^0.75^/day and metabolizable energy intake for gain = Mcal/kg empty body weight^0.75^/day.

The efficiency of use of metabolizable energy for gain estimated in this study is lower than that considered by the NRC [6]. In the present study, k_g_ was 41.9%, approximately 30% lower than the value used by NRC [6], which is 60% for non-lactating cows [31], while the NRC [6] suggests a k_g_ of 75% [3] for lactating cows. According to NRC [6], lactating animals are substantially more efficient than growing animals, and a higher k_g_ is suggested for animals that are not considerably changing the body composition [32]. The BR-CORTE [4] also found greater k_g_ for mature beef *Bos indicus* cows (53%) than the one we observed for Holstein × Gyr cows. However, our data does not support a k_g_ ranging from 53 to 60%. It is possible that pregnant animals, especially those close to parturition, might have their body composition altered [33], which does not support the k_g_ suggested by NRC [6]. According to the ARC [17], a high metabolizability (metabolizable energy/gross energy ratio) coincides with a greater k_g_; however, in our study the metabolizability was 0.57 ± 0.01, which might explain the lower k_g_ observed.

Nonetheless, Oss et al. [34] and Silva et al. [35] evaluating energy requirements of Holstein × Gyr bulls and heifers, reported a k_g_ of 30.5% (average body weight of 235 kg) and 40.8% (average body weight of 218 ± 36.5 kg), respectively. Therefore, our k_g_ was closer to values reported for growing animals, which have greater rates of energy deposited as protein than as fat. Moreover, the efficiency of gain proposed by BR-CORTE [4] demonstrated a gain composition with greater proportion of energy deposited as fat in adult animals, as discussed above. Nonetheless, we recommend a k_g_ of 41.9% for Holstein × Gyr adult cows. We also suggest, based on our results and previous literature [35, 36, 37] that the k_g_ reported in NRC [6] should be reviewed, because it might overestimate k_g_ for transition cows. The efficiency of utilization of body store reserves for milk production in early lactation is markedly higher than the efficiency of utilizing dietary metabolizable energy for tissue energy gain [32, 38]. This event, associated with the replenishment of body lipids mobilized at early lactation, leads to a higher k_g_ than that of gain of body protein.

### Net and metabolizable energy requirements for pregnancy

To calculate pregnancy requirements, the balance of pregnancy components (GEST) was used to estimate the retained energy related only to pregnancy. The same methodology was used by Ferrell et al. [1], Bell et al. [3], BR-CORTE [4] and Lage [39], however there were some particularities among them. The idea of GEST utilized in this study is like the one adopted by BR-CORTE [4]. The GEST component is the accretion in udder, uterus, and all the other components of the gravid uterus due to pregnancy. Following the establishment of GEST, a non-linear regression was fit to estimate GEST in Mcal as a function of days of pregnancy (Eq 8). The first derivate of Eq 8, adding a correction factor for expected calf body weight [6], was considered as NE_preg_.

$$NEpreg=0.02105\pm 0.6475\times exp^{\left(0.0141\pm 0.0017\times days\;of\;pregnancy\right)}\times \left(expected\;CBW/35\right)$$ (17)

(R^2^ = 0.741; RMSE = 136.3)

Where: NE_preg_ = net energy for pregnancy (Mcal/day; representing energy retained in the gravid uterus and mammary gland), CBW = calf body weight (kg).

Calf body weight (35 kg) was obtained according to average weight of Holstein × Gyr calves from Silva et al. [40] and Azevedo et al. [41]. The NRC [6] uses a linear regression equation to estimate NE_preg_ considering days of pregnancy and calf body weight, and the fetus accounts for approximately 80% of uterine dry weight [3]. However, non-linear regressions have been used to estimate animal’s development allowing a greater representation of biological growth [42].

**Fig 6:**
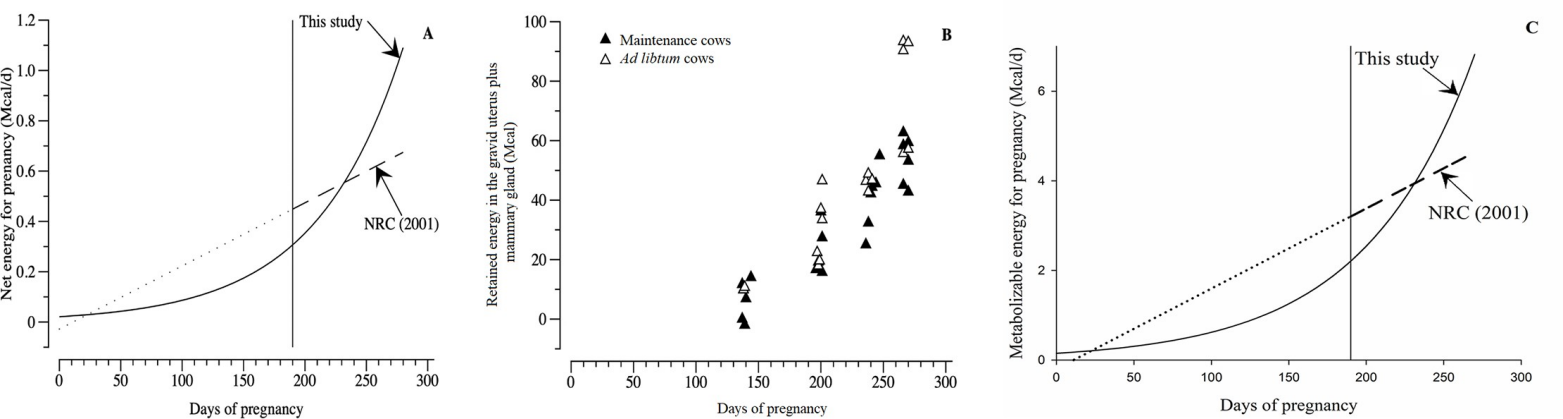
Net energy for pregnancy and retained energy in the gravid uterus. A–Estimation of net energy required for pregnancy (i.e., energy retained) by the NRC (2001) equation and the non-linear equation estimated in this study. The NRC (2001) equation for net energy retained (Mcal/day) is: (0.00318 × days of pregnancy– 0.0352) × (calf body weight (kg)/45). B–Relation between retained energy in the gravid uterus plus mammary gland and days of pregnancy. Open symbols refer to cows fed ad libitum and closed symbols refer to cows fed at maintenance level. Metabolizable energy for pregnancy. C—Estimation of metabolizable energy required for pregnancy (i.e., energy retained) by the NRC (2001) equation and the non-linear equation estimated in this study. The NRC (2001) equation for net energy retained (Mcal/day) is: [(0.00318 × days of pregnancy– 0.0352) × (calf body weight (kg)/45)]/0.14.

Estimations of energy requirements for pregnant cows by a linear [6] and non-linear regression (our study) are illustrated in Fig 6. According to the results presented in Fig 6A, NE_preg_ for crossbred Holstein × Gyr cows is lower than for purebred Holstein cows [6] until 230 days of pregnancy, approximately. After this period, estimated NE_preg_ surpass the requirements predicted by NRC [6]. Moreover, Fig 6B shows the pattern of retained energy in the gravid uterus and mammary gland, with an equal pattern to NE_preg_ and the accretion, illustrated in Fig 6B, allows a greater representation of energy requirements for pregnancy.

It is important to highlight that the greatest amount of retained energy in the gravid uterus and mammary gland is observed for three cows fed ad libtum on day 270 (Fig 6B). Although these observations with high retained energy present a greater distance from the observed points of the group (day 270), they have a considerable impact on the exponential nature of the chosen model. However, as feed regimen was accounted in the model as a random effect, that variation was controlled in this study. Nevertheless, we encourage future studies evaluating the effect of feed regimen in pregnant cows and its impact on pregnancy requirements.

Our model shows greater similarity among biological growth models [38] than do linear models [42]. In addition, the NASEM [27] and INRA [8] also considers the use of a non-linear regression to estimate pregnancy requirements. Their values are much closer to ours when compared to NRC [6]. Fig 6C shows metabolizable energy for pregnancy obtained from NRC [6] equation and the equation from this study. As in NE_preg_ for crossbred Holstein × Gyr cows, metabolizable energy for pregnancy is lower than for purebred Holstein cows [6] until 235 days of pregnancy, approximately. After this period, estimated NE_preg_ surpass the requirements predicted by NRC [6], indicating greater increase in requirements for pregnancy after 230 days.

Lage [40] found NE_preg_, represented by retained energy in gravid uterus and mammary gland for Holstein × Gyr cows of 2.70, 2.71, and 2.88 Mcal/day at 180, 210 and 240 days of pregnancy, respectively. Net energy requirements obtained by Lage [39] are closer to NRC [6] when compared to our values. However, observing NE_preg_ obtained in our study, we found similar results to Ferrell et al. [1] as they obtained retained energy for pregnancy (Table 1). This proximity of values for Holstein × Gyr cows and Hereford heifers could be principally due to the exponential models used to estimate energy content in the gravid uterus. Net energy for pregnancy for beef cattle [27] is estimated by a similar non-linear equation from Ferrell et al. [1]. Although values are close for NE_preg_ and for the efficiency of use of ME_preg_, the estimated required energy is different between this and Ferrell et al [1] work. There is a substantial increase in metabolizable energy requirements for pregnancy from day 220 to the end of pregnancy in Ferrell et al. [1] estimations which accounts for approximately an additional 500 kcal/day when compared to this study.

**Table 1:** Metabolizable and net energy requirements for pregnant Holstein × Gyr cows.

Days of pregnancy	Net energy for pregnancy (kcal/day)		Metabolizable energy for pregnancy (kcal/day)	
	This study^1^	Ferrell et al. (1976)^2^	This study^1^	Ferrell et al. (1976)^2^
100	87	36	620	257
130	133	74	947	527
160	204	143	1445	1021
190	311	263	2206	1879
220	475	457	3368	3264
250	725	752	5141	5371
280	1107	1167	7848	8336

^1^ Energy requirements for pregnancy calculated according to the estimated equation in this study. Efficiency of utilization of metabolizable energy for pregnancy = 14.1%. Net energy for pregnancy denotes energy retained in gravid uterus and mammary gland.

^2^ Energy requirements for pregnancy adapted from Ferrell et al. [1]. Efficiency of utilization of metabolizable energy for pregnancy = 14%. Net energy for pregnancy denotes energy retained in gravid uterus and mammary gland.

**Fig 7:**
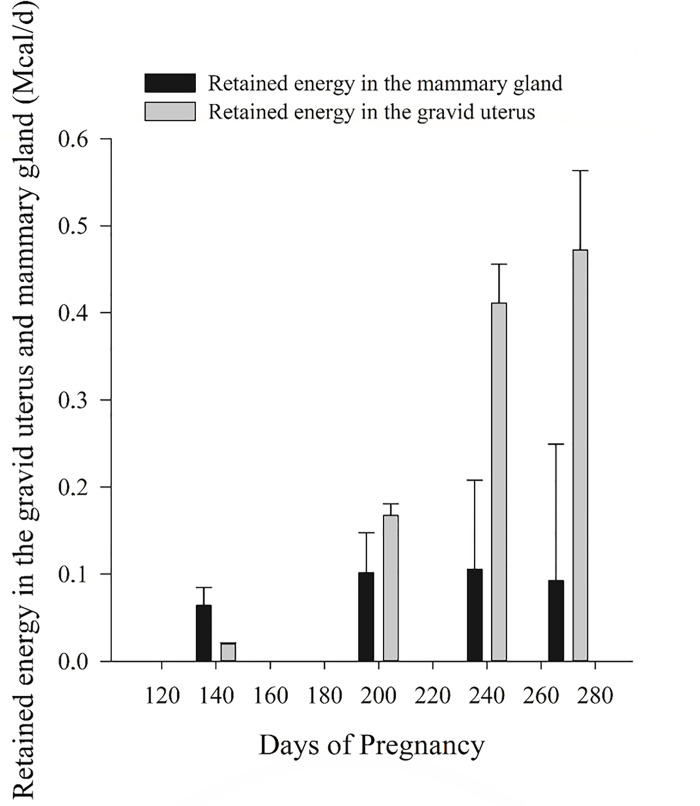
Retained energy in the gravid uterus and mammary gland. Pattern of retained energy in the gravid uterus and mammary gland according to days of pregnancy.

The retained energy in the gravid uterus and mammary gland through days of pregnancy may be observed in Fig 7. There is a greater increase of retained energy in the gravid uterus than in mammary gland. From 140 to 240 days the amount of energy retained in the gravid uterus is greater than from 240 to 270 days. It may be an indicative of uterus growth to support fetus development during the final period of pregnancy. Changes in mammary gland also account for pregnancy requirements. However, changes are smaller when compared to the gravid uterus. This pattern of energy deposition may occur because the involution of the mammary gland itself, after lactation, is smaller in proportion when compared to uterus involution. The uterus grows to a size enough to support the size of a calf and after birth, it recovers its initial size.

Efficiency of use of metabolizable energy for pregnancy was estimated by the iterative method. The obtained value for k_preg_ was 14.1 ± 0.41%, which is very close to values obtained by Ferrell et al. [1], 14%; Lage [40], 12.5% and BR-CORTE [4], 12%. The NRC [6] uses k_preg_ suggested by Ferrell et al. [1]. Metabolizable energy for pregnancy estimated in this study follows the same pattern of accretion as NE_preg_, surpassing the NRC [6] values at the end of the gestational period.

The estimated k_preg_ is lower than any other efficiencies of energy utilization (k_m_ and k_g_). This inefficiency is probably because of the energetic cost associated with maintenance of pregnancy products (gravid uterus and mammary gland), which may be related to oxidative metabolism [43]. A great part of the energy available for pregnancy is expended as heat production [43], or with greater rates of muscular turnover, to offer amino acids as energy source to the fetus [39]. Protein is the most abundant organic constituent of conceptus tissues [1]. Therefore, according to Hammond [44] the homeorhetic effect in cows, mainly pregnant, is a mechanism able to direct nutrients to tissues with high metabolic rates, as the gravid uterus, improving the energy parturition for fetal development.

**Fig 8:**
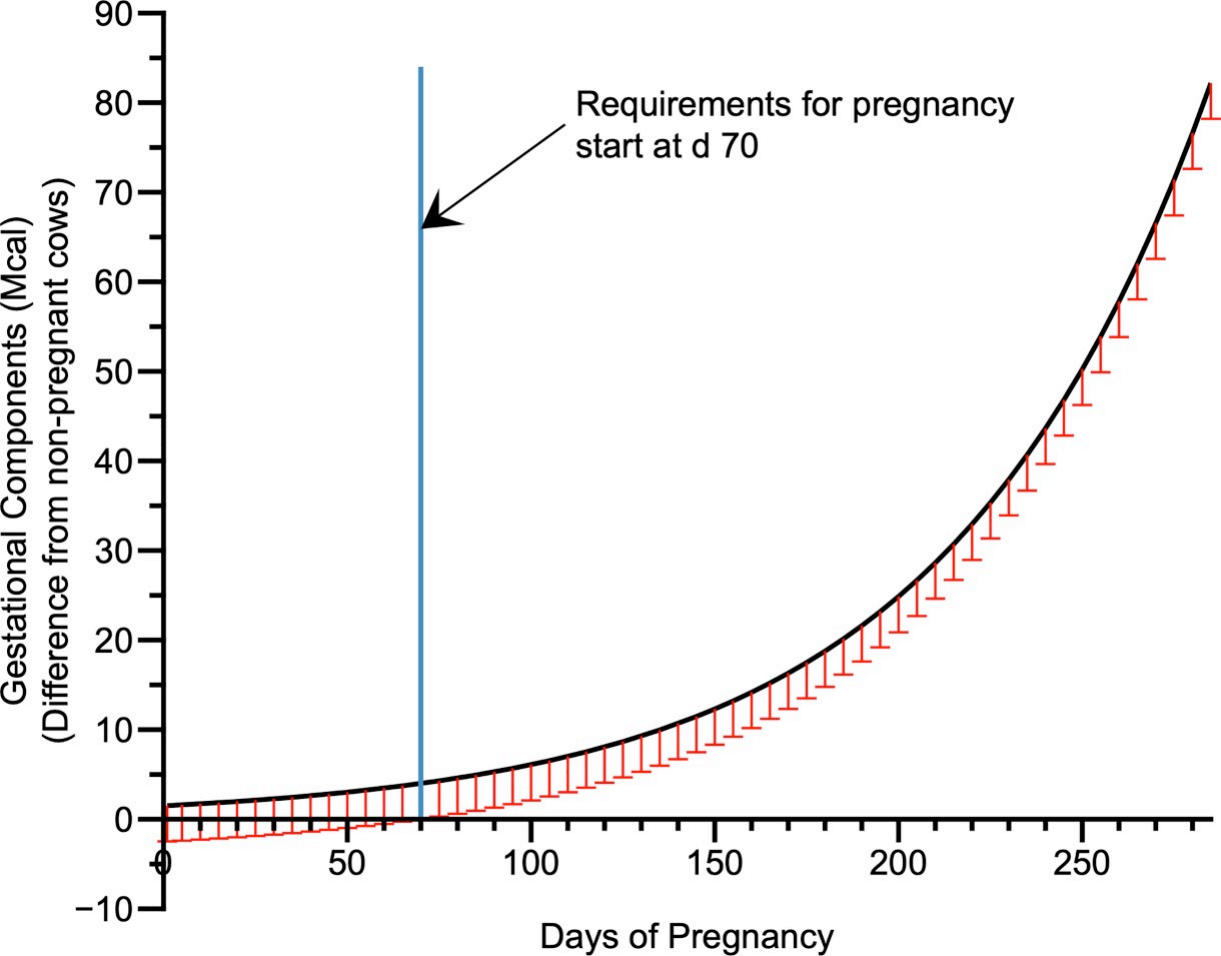
Initial point of energy requirement for pregnancy.

Another important point to consider is the precise moment when pregnancy requirements should be added to dietary requirements. After estimating energy requirements for pregnancy, we determined the day of pregnancy when pregnancy requirements were statistically different from non-pregnant cows using the lower confidence limit of the retained energy in the gravid uterus (P < 0.05). Our data indicate that energy requirements for pregnancy should be accounted from 70 days of pregnancy onwards (Fig 8). The NRC [6] suggests that pregnancy requirements begin only at day 190 of pregnancy. However, it is well documented in the literature that fetus development begins before 190 days of pregnancy, with essential processes as organogenesis and myogenesis [45,46]. Therefore, we suggest considering pregnancy requirements from 70 days of pregnancy onwards, because accounting pregnancy requirement only from day 190 of pregnacy, may result in undernourishment of both the cow and the fetus.

## Conclusion

In conclusion, maintenance requirements for non-pregnant Holstein × Gyr cows are 5% lower than for pregnant cows. The efficiency of use of metabolizable energy for maintenance for Holstein × Gyr cows is lower than the recommendations of NRC [6]. Additionally, we recommend using data from non-pregnant animals to estimate energy requirements for gain. Furthermore, we believe that the proposed non-linear equation to estimate net energy requirements for pregnancy are more adequate than current NRC equation, and should be recommended for Holstein × Gyr. Lastly, our data suggests the beginning of pregnancy requirements from 70 days of pregnancy, thus we assumed this point as the beginning of biological need for nutrients from the fetus.

## Supporting information

S1 File (XLSX)

S2 File (PDF)

S3 File (PDF)

S4 File (PDF)

S5 File (PDF)

## References

[1] Ferrell CL, Garrett WN, Hinman N, Grichting G. Energy utilization by pregnant and non-pregnant heifers. J Anim Sci. 1976;42: 937–950. 10.2527/jas1976.424937x 1262293

[2] Moe PW, Tyrrell HF. Metabolizable energy requirements of pregnant dairy cows. J Dairy Sci. 1972;55: 480–483. 10.3168/jds.S0022-0302(72)85519-X 5063018

[3] Bell AW, Slepetis R, Ehrhardt UA. Growth and accretion of energy and protein in the gravid uterus during late pregnancy in Holstein cows. J Dairy Sci. 1995;78: 1954–1961. 10.3168/jds.s0022-0302(95)76821-7 8550905

[4] BR-CORTE. ‘Nutrient requirements of Zebu and crossbred.’ (3^rd^ ed). (Suprema Gráfica e Editora: Visconde do Rio Branco. MG, Brazil); 2016.

[5] AFRC ‘Energy and protein requirements of ruminants.’ (1st ed). (CAB International: Wallingford, UK); 1993.

[6] NRC ‘Nutrient requirements of dairy cattle (7th ed).’ (National Academic. Press: Washington DC); 2001.

[7] CSIRO; `Nutrient requirements of domesticated ruminants.' (Australia Agricultural Council: Victoria); 1965.

[8] INRA. ‘Alimentation des bovins, ovins et caprins. Besoins des animaux. Valeurs des aliments.’ (Agabriel J., ed. Editions Quae, Versailles, France); 2007.

[9] Ruas JRM, Menezes AC, Queiroz DS, Silva EA da, Costa MD da. Cruzamentos para a produção sustentável de leite. EMBRAPA—Pesquisa, desenvolvimento e inovação para a sustentabilidade da bovinocultura leiteira 10th ed 2011: 189–190.

[10] Carvalho PHA, Borges ALCC, Silva RR, Lage HF, Vivenza PAD, Ruas JRM, et al Energy metabolism and partition of lactating Zebu and crossbred Zebu cows in different planes of nutrition. Loor JJ, editor. PLoS One. 2018;13: 1–10. 10.1371/journal.pone.0202088 30118491 PMC6097685

[11] Oliveira AS. Meta-analysis of feeding trials to estimate energy requirements of dairy cows under tropical condition. Anim Feed Sci Technol. 2015;210: 94–103. 10.1016/j.anifeedsci.2015.10.006

[12] Rotta PP, Valadares Filho SC, Gionbelli TRS, Costa e Silva LF, Engle TE, Marcondes MI, et al Effects of day of gestation and feeding regimen in Holstein × Gyr cows: II. Maternal and fetal visceral organ mass. J Dairy Sci. 2015;98: 1–13. 10.3168/jds.2014-8433 25726105

[13] Rotta PP, Valadares Filho SC, Gionbelli TRS, Costa e Silva LF, Engle TE, Marcondes MI, et al Effects of day of gestation and feeding regimen in Holstein × Gyr cows: III. Placental adaptations and placentome gene expression. J Dairy Sci. 2015;98: 3224–3235. 10.3168/jds.2014-8283 25747832

[14] Rotta PP, Valadares Filho SC, Gionbelli TRS, Costa e Silva LF, Engle TE, Marcondes MI, et al Effects of day of gestation and feeding regimen in Holstein × Gyr cows: I. Apparent total-tract digestibility, nitrogen balance, and fat deposition. J Dairy Sci. 2015;98: 3197–3210. 10.3168/jds.2014-8280 25726112

[15] AOAC International. ‘Official Methods of Analysis.’ (17th ed). (AOAC International, Arlington, VA); 2000.

[16] AOAC International. ‘Official Methods of Analysis.’ (18th ed). (AOAC International, Gaithersburg, MD); 2006.

[17] ARC. ‘The Nutrient Requirements of Ruminants Livestock’ Agricultural Research Council. (CAB International: London); 1980.

[18] Lippke H, Ellis WC, Jacobs BF. Recovery of indigestible fiber from feces of sheep and cattle on forage diets. J Dairy Sci. 1986;69: 403–412. 10.3168/jds.S0022-0302(86)80418-0

[19] Valente TNP, Detmann E, Queiroz AC, Valadares Filho S C, Gomes DI, Figueiras JF. Evaluation of ruminal degradation profiles of forages using bags made from different textiles. Rev Bras Zootec. 2011;40: 2565–2573. 10.1590/S1516-35982011001100039

[20] Detmann E, Valadares Filho SC. On the estimation of non-fibrous carbohydrates in feeds and diets. Braz J Vet Anim Sci. 2011; 62:980–984. 10.1590/S0102-09352010000400030

[21] NRC. ‘Nutrient Requirements of Beef Cattle’. (7th ed). (National Academy Press, Washington, DC); 2000.

[22] Ferrell CL, Jenkins TG. Body composition and energy utilization by steers of diverse genotypes fed a high-concentrate diet during the finishing period: I. Angus, Belgian Blue, Hereford, and Piemontese sires. J Anim Sci. 1998; 76:637–646. 10.2527/1998.762637x 9498375

[23] Garrett WN. Energy utilization by growing cattle as determined in 72 comparative slaughter experiments. In: Proc 8th Symposium of Energy Metabolism. London, UK. University of Cambridge, Cambridge, UK; 1980. Pp. 3−7.

[24] Marcondes MI, Tedeschi LO, Valadares Filho SC, Gionbelli MP. Predicting efficiency of use of metabolizable energy to net energy for gain and maintenance of Nellore cattle. J Anim Sci. 2013; 4887–4898. 10.2527/jas.2011-4051 23978609

[25] Lage HF. ‘Partição da energia e exigência de energia líquida para mantença de novilhas Gir e F1 Holandês × Gir’. MSc Thesis. Federal University of Minas Gerais, Brazil. 2011. http://hdl.handle.net/1843/BUOS-9MYLCN.

[26] Solis JC, Byers FM, Schelling GT, Long CR, Greene LW. Maintenance requirements and energetic efficiency of cows of different breed types. J Anim Sci. 1988;66: 764–773. 10.2527/jas1988.663764x 3378932

[27] NASEM. ‘National Academies of Sciences, Engineering, and Medicine’. Nutrient Requirements of Beef Cattle. (8th revised ed). (The National Academies Press, Washington, DC); 2016.

[28] Bell AW. Regulation of organic nutrient metabolism during transition from late pregnancy to early lactation. J Anim Sci. 1995;73: 2804–2819. 10.2527/1995.7392804x 8582872

[29] Musharaf NA, Latshaw JD. Heat increment as affected by protein and amino acid nutrition. Worlds Poult Sci J. 1999;55: 233–240. 10.1079/wps19990017

[30] Sguizzato ALL, Marcondes MI, Valadares Filho SC, Caton J, Neville TL, Machado FS, et al Body composition changes of crossbred Holstein × Gyr cows and conceptus during pregnancy. J Dairy Sci. 2020 10.3168/jds.2019-17490 31954558

[31] Moe PW, Tyrrell HF, Flatt WP. Partial efficiency of energy use for maintenance, lactation, body gain and gestation in the dairy cow. In: Proc. 5th Symposium on Energy Metabolism. Vitznau, Switzerland; 1970.

[32] Moe PW, Tyrrell HF, Flatt WP. Energetics of body tissue mobilization. J. Dairy Sci. 1971;54: 548–553. 10.3168/jds.S0022-0302(71)85886-1 5106186

[33] Kenéz Á, Kulcsár A, Kluge F, Benbelkacem I, Hansen K, Locher L, et al Changes of adipose tissue morphology and composition during late pregnancy and early lactation in dairy cows. PLoS One. 2015;10: 1–11. 10.1371/journal.pone.0127208 25978720 PMC4433245

[34] Oss DB, Machado FS, Tomich TR, Pereira LGR, Campos MM, Castro MMD, et al Energy and protein requirements of crossbred (Holstein × Gyr) growing bulls. J Dairy Sci. 2017;100: 2603–2613. 10.3168/jds.2016-11414 28161164

[35] Silva FAS, Valadares Filho SC, Rennó LN, Zanetti D, Costa e Silva LF, Godoi LA, et al Energy and protein requirements for growth of Holstein × Gyr heifers. J Anim Physiol Anim Nutr (Berl). 2018;102: 82–93. 10.1111/jpn.12661 28299852

[36] Rotta PP, Valadares Filho SC, Detmann E, Costa e Silva LF, Villadiego FAC, Burgos EMG, et al Nutrient requirements of energy and protein for Holstein × Zebu bulls finished in feedlot. Semin Ciências Agrárias. 2013;34: 2523–2534. 10.5433/1679-0359.2013v34n5p2523

[37] Gionbelli MP, Duarte MS, Valadares Filho SC, Detmann E, Gionbelli TRS, Zanetti D, et al Energy requirements for pregnant and nonpregnant Nellore cows. J Anim Sci. 2015; 93:547.

[38] Moraes LE, Kebreab E, Strathe AB, Dijkstra J, France J, Casper DP, et al Multivariate and univariate analysis of energy balance data from lactating dairy cows. J Dairy Sci. 2015;98: 4012–4029. 10.3168/jds.2014-8995 25892698

[39] Lage HF. ‘Partição da energia e exigências nutricionais no terço final da gestação e avaliação do perfil metabólico durante o período de transição de vacas Gir e F1 Holandês × Gir’. D. Sc. Dissertation. Federal University of Minas Gerais, Brazil. 2015.

[40] Silva AL, Marcondes MI, Detmann E, Campos MM, Machado FS, Filho SCV, et al Determination of energy and protein requirements for crossbred Holstein × Gyr preweaned dairy calves. J Dairy Sci. 2017;100: 1170–1178. 10.3168/jds.2016-11197 27939536

[41] Azevedo RA, Martins LF, Tiveron PM, Teixeira AM, Bittar CMM, Antunes LCMS, et al Perguntas e Respostas Alta CRIA. Perguntas e Respostas Alta CRIA. 2019 10.26626/978-85-5953-053-7.2019b0001

[42] Tedeschi LO, Boin C, Nardon RF, Leme PR. Estudo da curva de crescimento de animais da raça guzerá e seus cruzamentos alimentados a pasto, com e sem suplementação. 1. Análise e seleção das funções não-lineares. Rev Bras Zootec. 2000;29: 630–637. 10.1590/S1516-35982002000700012

[43] Bauman DE, Currie WB. Partitioning of nutrients during pregnancy and lactation: A review of mechanisms involving homeostasis and homeorhesis. J Dairy Sci. 1980;63: 1514–1529. 10.3168/jds.s0022-0302(80)83111-0 7000867

[44] Hammond J. Animal breeding in relation to nutrition and environmental conditions. Biol Rev. 1947;22: 195–213. 10.1111/j.1469-185x.1947.tb00330.x 20253192

[45] Du M, Tong J, Zhao J, Underwood KR, Zhu M, Ford SP, et al Fetal programming of skeletal muscle development in ruminant animals. J Anim Sci. 2010; 88. 10.2527/jas.2009-2311 19717774

[46] Funston RN, Larson DM, Vonnahme KA. Effects of maternal nutrition on conceptus growth and offspring performance: implications for beef cattle production. J Anim Sci. 2010; 88 10.2527/jas.2009-2351 19820049

