# Supplementary material for: Energy requirements for pregnant dairy cows
Source: PLoS One. 2020 Jul 7;15(7):e0235619. doi: 10.1371/journal.pone.0235619 (PMC7340303; doi:10.1371/journal.pone.0235619)
Supplement: S2 File — (PDF) [file pone.0235619.s002.pdf]

TODAS PRENHES TESTANDO DIETAS

The NLMIXED Procedure

Specifications	
**Data Set**	WORK.IMPORT
**Dependent Variable**	CTRET
**Distribution for Dependent Variable**	Normal
**Optimization Technique**	Dual Quasi-Newton
**Integration Method**	None

Dimensions	
**Observations Used**	42
**Observations Not Used**	14
**Total Observations**	56
**Parameters**	5

Initial Parameters					
**a1**	**b1**	**a2**	**b2**	**S2**	**Negative Log Likelihood**
0.05	1.05	0.05	1.05	0.21	180.192729

Iteration History					
**Iteration**	**Calls**	**Negative Log Likelihood**	**Difference**	**Maximum Gradient**	**Slope**
**1**	7	137.0943	43.09844	1318.90	-726430
**2**	11	98.0169500	39.07734	294.345	-21044.7
**3**	13	81.6919290	16.32502	2487.06	-1122.40
**4**	17	61.3178859	20.37404	426.172	-20.1378
**5**	19	59.0393349	2.278551	507.269	-6.39925
**6**	22	57.5562425	1.483092	155.263	-1.51583
**7**	24	55.8671933	1.689049	107.243	-0.85808
**8**	27	55.7263640	0.140829	45.8212	-0.43716
**9**	29	55.5777096	0.148654	37.9049	-0.19478
**10**	32	55.5074674	0.070242	23.6671	-0.06518
**11**	34	55.4289013	0.078566	35.8151	-0.04980
**12**	37	55.4109136	0.017988	16.6072	-0.01709
**13**	40	55.4084834	0.00243	14.5478	-0.00342
**14**	43	55.4079725	0.000511	3.47603	-0.00052
**15**	46	55.4078084	0.000164	0.92525	-0.00020
**16**	49	55.4077975	0.000011	0.038687	-0.00001
**17**	52	55.4077973	1.807E-7	0.019494	-2.74E-7

TODAS PRENHES TESTANDO DIETAS

The NLMIXED Procedure

NOTE: GCONV convergence criterion satisfied.

Fit Statistics	
**-2 Log Likelihood**	110.8
**AIC (smaller is better)**	120.8
**AICC (smaller is better)**	122.5
**BIC (smaller is better)**	129.5

Parameter Estimates

Parameter	Estimate	Standard Error	DF	t Value	Pr > |t|	95% Confidence Limits		Gradient
**a1**	0.06100	0.02599	42	2.35	0.0237	0.008550	0.1134	0.002597
**b1**	0.6818	0.2842	42	2.40	0.0210	0.1082	1.2553	-0.00016
**a2**	0.06006	0.002131	42	28.18	<.0001	0.05576	0.06436	0.019494
**b2**	0.6017	0.1438	42	4.18	0.0001	0.3115	0.8919	0.000057
**S2**	0.8192	0.1788	42	4.58	<.0001	0.4584	1.1800	0.000094

Correlation Matrix of Parameter Estimates

	a1	b1	a2	b2	S2
**a1**	1.0000	0.9606	0.0000	0.0000	0.0002
**b1**	0.9606	1.0000	0.0000	0.0000	0.0002
**a2**	0.0000	0.0000	1.0000	0.0752	0.0002
**b2**	0.0000	0.0000	0.0752	1.0000	0.0000
**S2**	0.0002	0.0002	0.0002	0.0000	1.0000

Additional Estimates

Label	Estimate	Standard Error	DF	t Value	Pr > |t|	Alpha	Lower	Upper
**Va1-Pa2**	0.000935	0.02608	42	0.04	0.9716	0.05	-0.05169	0.05356
**Vb1-Pb2**	0.08007	0.3185	42	0.25	0.8027	0.05	-0.5627	0.7229

Correlation Matrix of Additional Estimates

Label	Corr1	Corr2
**Va1-Pa2**	1.0000	0.8570
**Vb1-Pb2**	0.8570	1.0000

GANHO APENAS PRENHES

The NLIN Procedure
Dependent Variable CTRET
Method: Gauss-Newton

Iterative Phase			
**Iter**	**a**	**b**	**Sum of Squares**
**0**	0.0860	1.0500	169.1
**1**	0.0604	0.6979	34.8308
**2**	0.0600	0.6544	34.5556
**3**	0.0600	0.6562	34.5551
**4**	0.0600	0.6562	34.5551

Note: Missing values were generated as a result of performing an operation on missing values. Each place is given by (number of times) AT (statement)/(line):(column).

**14 AT**	1/96:25

NOTE: Convergence criterion met.

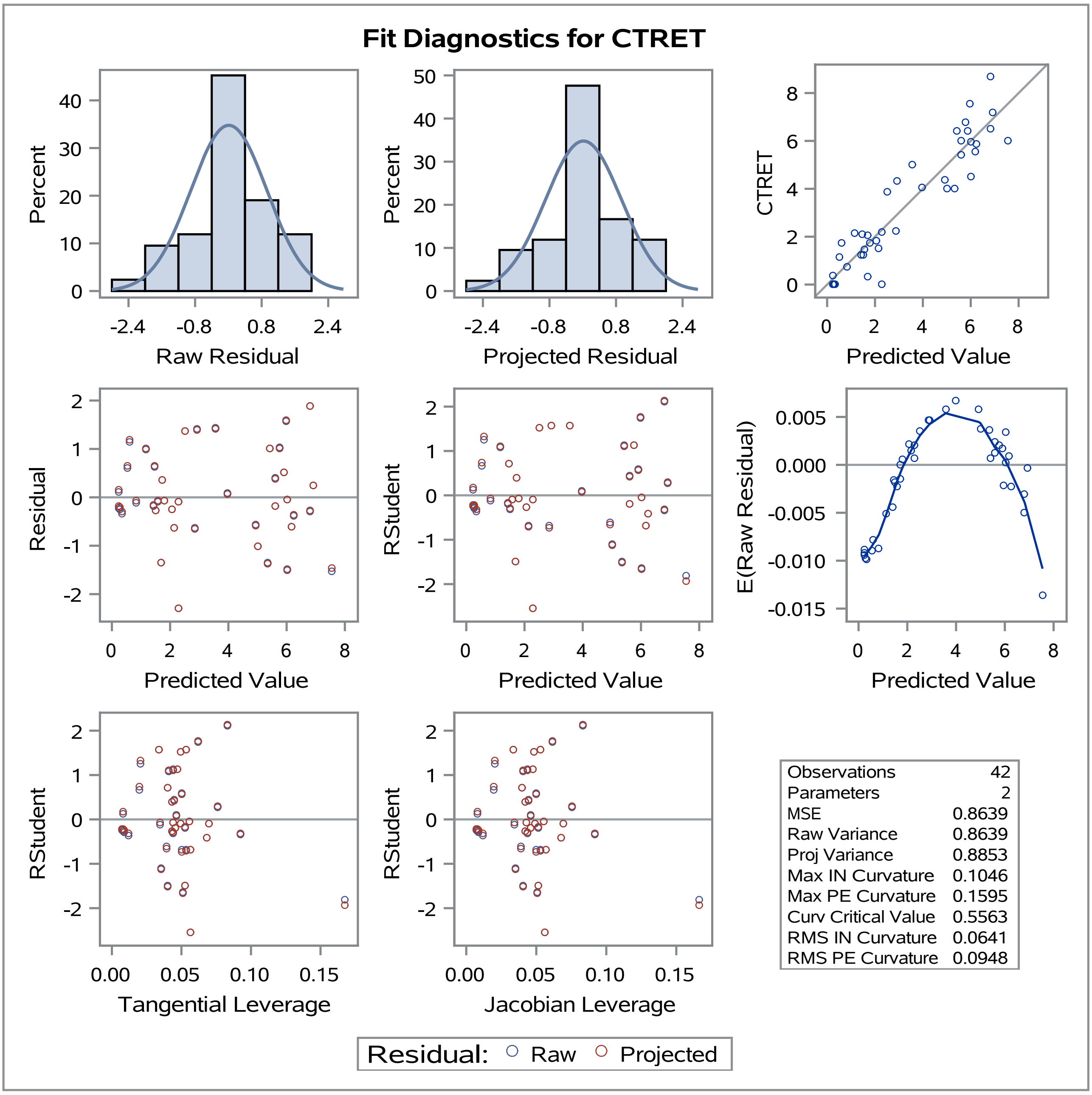

GANHO APENAS PRENHES

The NLIN Procedure
Dependent Variable CTRET
Method: Gauss-Newton

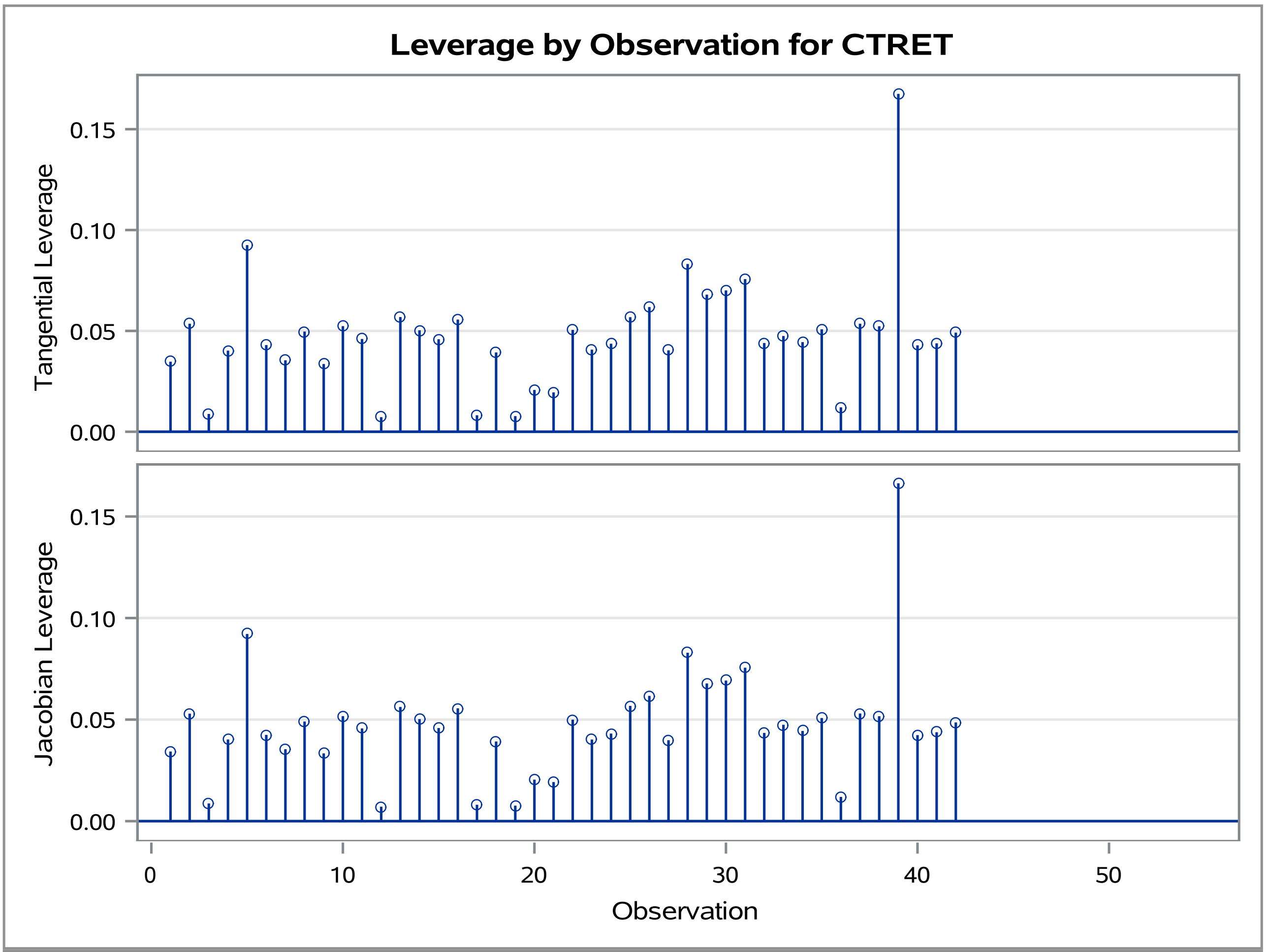

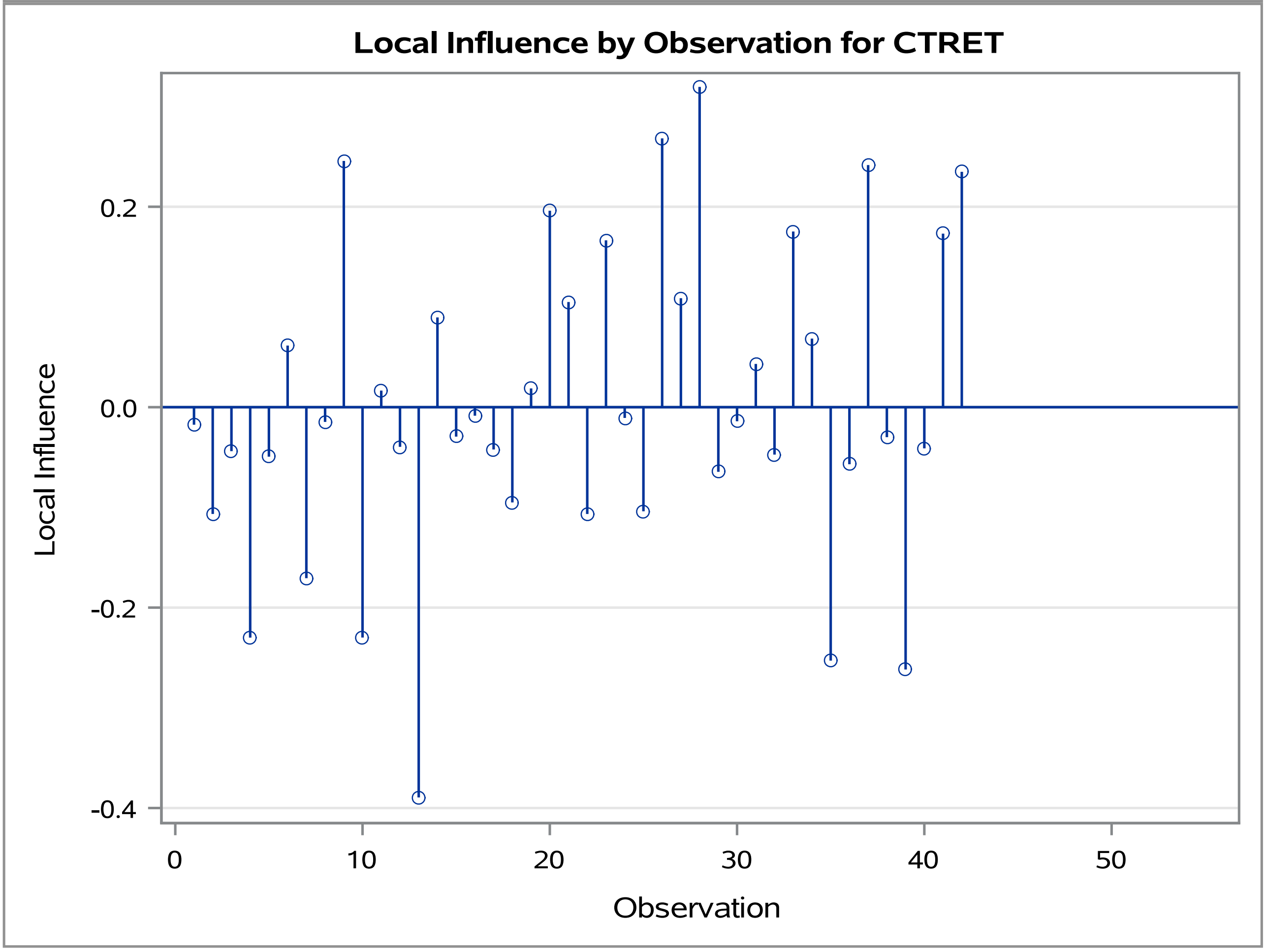

GANHO APENAS PRENHES

The NLIN Procedure

Estimation Summary	
**Method**	Gauss-Newton
**Iterations**	4
**R**	6.051E-7
**PPC(b)**	4.228E-7
**RPC(b)**	0.000032
**Object**	2.03E-9
**Objective**	34.55506
**Observations Read**	56
**Observations Used**	42
**Observations Missing**	14

Note: An intercept was not specified for this model.

Source	DF	Sum of Squares	Mean Square	F Value	Approx Pr > F
**Model**	2	712.7	356.4	412.52	<.0001
**Error**	40	34.5551	0.8639		
**Uncorrected Total**	42	747.3			

Parameter	Estimate	Approx Std Error	Approximate 95% Confidence Limits	
**a**	0.0600	0.00217	0.0556	0.0644
**b**	0.6562	0.0725	0.5097	0.8027

Approximate Correlation Matrix		
	**a**	**b**
**a**	1.0000000	0.2712055
**b**	0.2712055	1.0000000

GANHO APENAS PRENHES

The REG Procedure
Model: MODEL1
Dependent Variable: Pred Predicted Value

**Number of Observations Read**	56
**Number of Observations Used**	42
**Number of Observations with Missing Values**	14

Analysis of Variance					
Source	DF	Sum of Squares	Mean Square	F Value	Pr > F
**Model**	1	202.07130	202.07130	264.76	<.0001
**Error**	40	30.52947	0.76324		
**Corrected Total**	41	232.60077			

**Root MSE**	0.87363	**R-Square**	0.8687
**Dependent Mean**	3.38163	**Adj R-Sq**	0.8655
**Coeff Var**	25.83474		

Parameter Estimates						
Variable	Label	DF	Parameter Estimate	Standard Error	t Value	Pr > |t|
**Intercept**	Intercept	1	0.39479	0.22775	1.73	0.0907
**CTRET**	CTRET	1	0.87852	0.05399	16.27	<.0001

GANHO APENAS PRENHES

The REG Procedure
Model: MODEL1
Dependent Variable: Pred Predicted Value

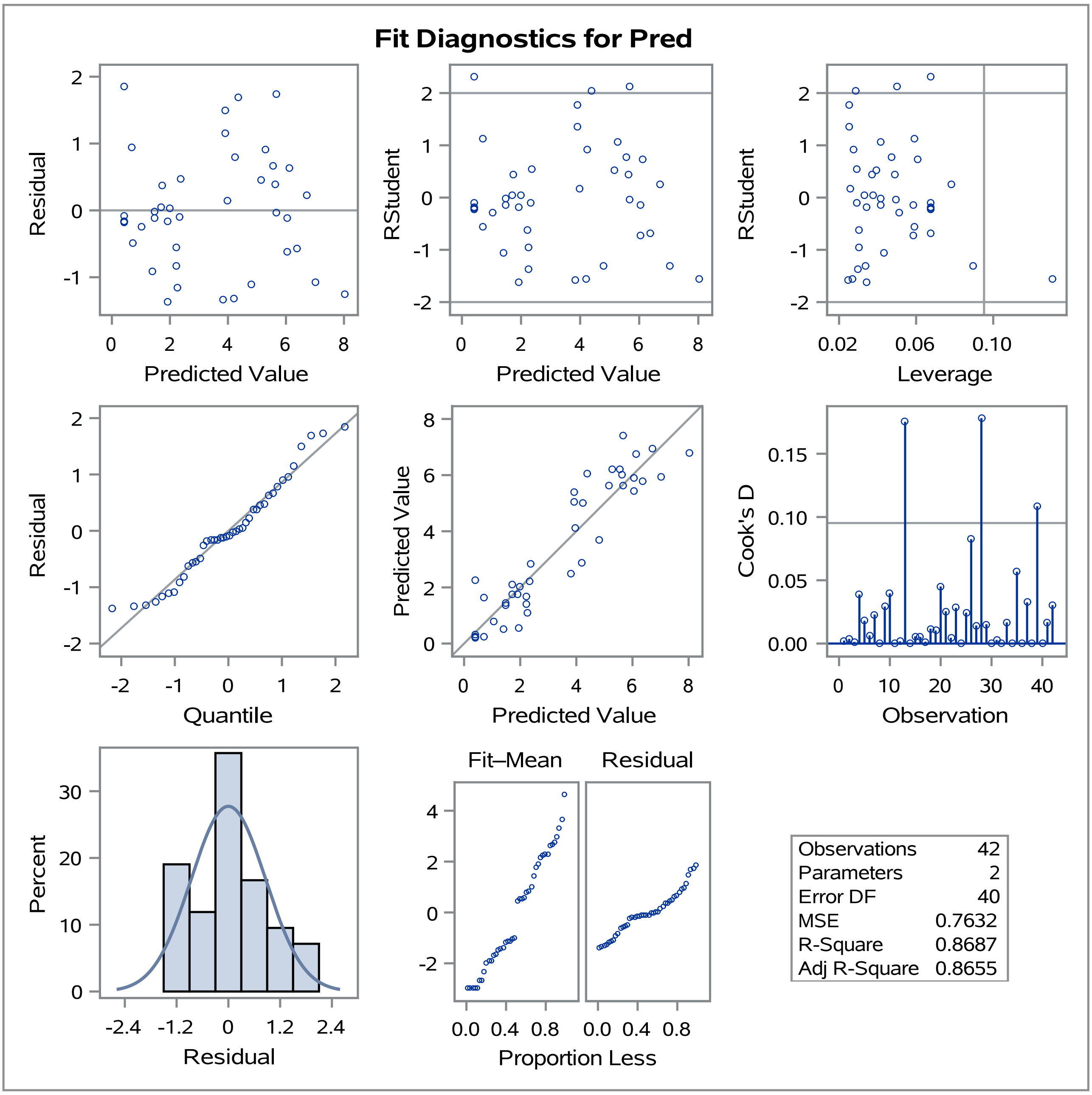

GANHO APENAS PRENHES

The REG Procedure
Model: MODEL1
Dependent Variable: Pred Predicted Value

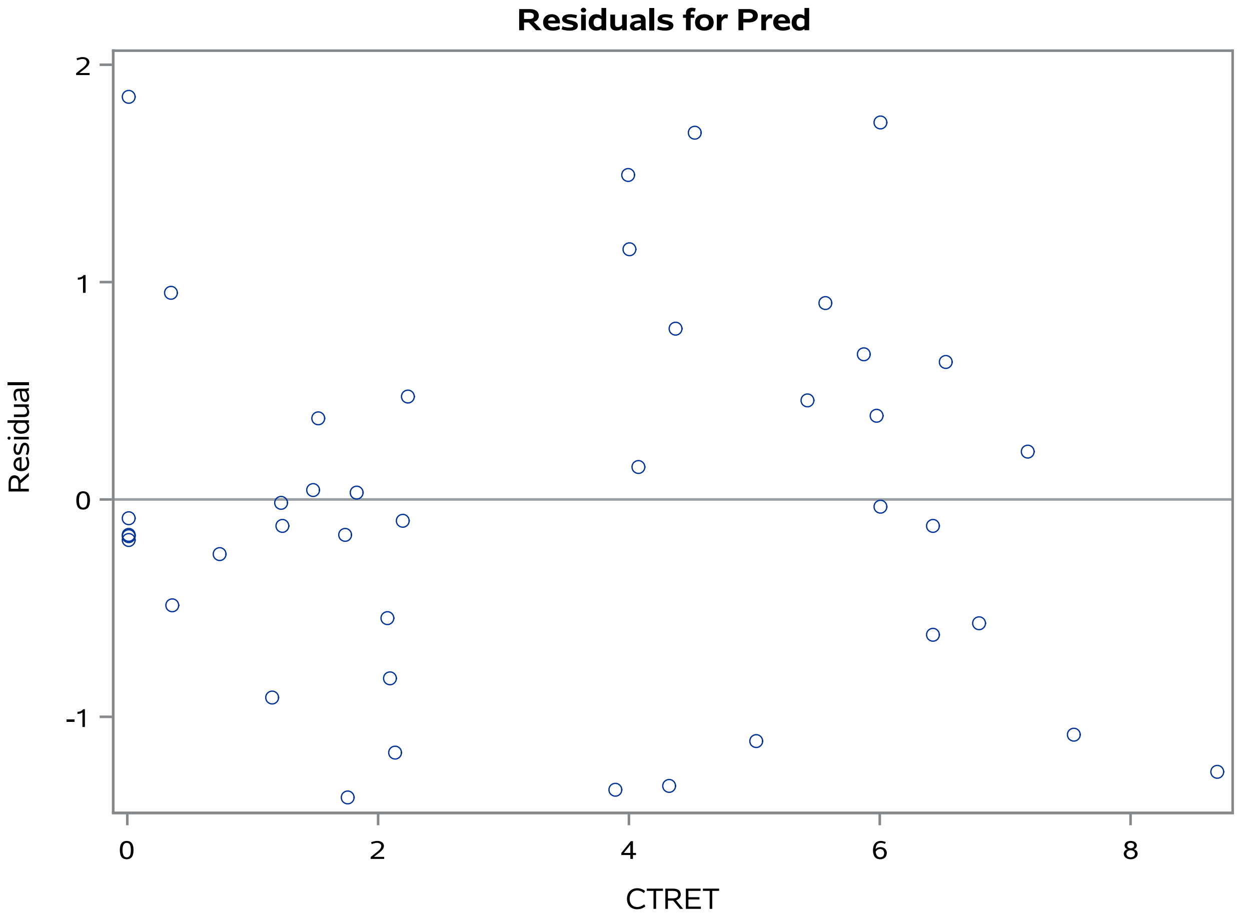

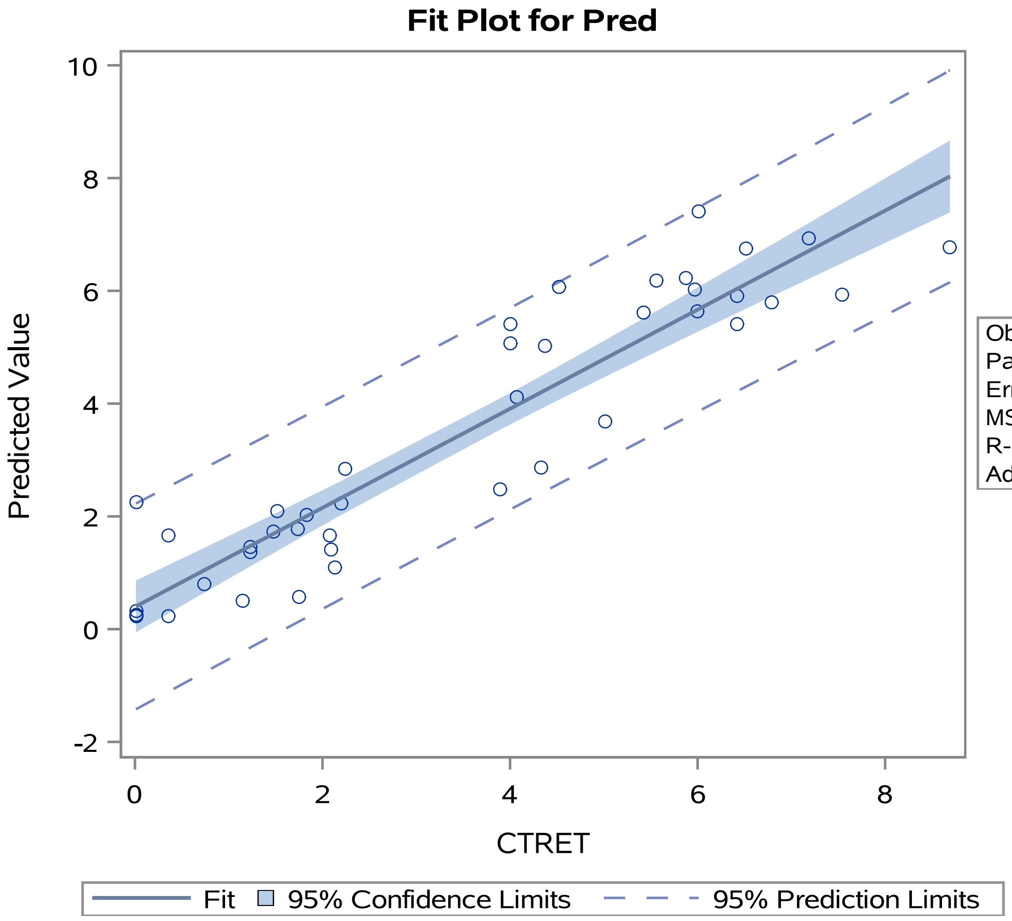

Observations	42
Parameters	2
Error DF	40
MSE	0.7632
R-Square	0.8687
Adj R-Square	0.8655

GANHO APENAS PRENHES

The MEANS Procedure

Analysis Variable : MSE				
N	Mean	Std Dev	Minimum	Maximum
42	0.8192135	1.1312515	0.000499209	5.0486955
