# Supplementary material for: Energy requirements for pregnant dairy cows
Source: PLoS One. 2020 Jul 7;15(7):e0235619. doi: 10.1371/journal.pone.0235619 (PMC7340303; doi:10.1371/journal.pone.0235619)
Supplement: S3 File — (PDF) [file pone.0235619.s003.pdf]

The Mixed Procedure

Model Information	
**Data Set**	WORK.NEW
**Dependent Variable**	ER_UTM_dia
**Covariance Structure**	Diagonal
**Estimation Method**	REML
**Residual Variance Method**	Profile
**Fixed Effects SE Method**	Model-Based
**Degrees of Freedom Method**	Residual

Dimensions	
**Covariance Parameters**	1
**Columns in X**	2
**Columns in Z**	0
**Subjects**	1
**Max Obs per Subject**	11

Number of Observations	
**Number of Observations Read**	26
**Number of Observations Used**	11
**Number of Observations Not Used**	15

Covariance Parameter Estimates

Cov Parm	Estimate	Standard Error	Z Value	Pr > Z
**Residual**	0.000095	0.000045	2.12	0.0169

Fit Statistics	
**-2 Res Log Likelihood**	-58.9
**AIC (Smaller is Better)**	-56.9
**AICC (Smaller is Better)**	-56.3
**BIC (Smaller is Better)**	-56.7

Solution for Fixed Effects

Effect	Estimate	Standard Error	DF	t Value	Pr > |t|	Alpha	Lower	Upper
**Intercept**	-0.05574	0.01267	9	-4.40	0.0017	0.05	-0.08440	-0.02708
**CEM_UTM**	0.4189	0.05420	9	7.73	<.0001	0.05	0.2963	0.5415

The Mixed Procedure

Type 3 Tests of Fixed Effects				
Effect	Num DF	Den DF	F Value	Pr > F
CEM_UTM	1	9	59.74	<.0001

The REG Procedure
Model: MODEL1
Dependent Variable: Pred Predicted

**Number of Observations Read**	26
**Number of Observations Used**	11
**Number of Observations with Missing Values**	15

Analysis of Variance					
**Source**	**DF**	**Sum of Squares**	**Mean Square**	**F Value**	**Pr > F**
**Model**	1	0.00492	0.00492	59.74	<.0001
**Error**	9	0.00074100	0.00008233		
**Corrected Total**	10	0.00566			

**Root MSE**	0.00907	**R-Square**	0.8691
**Dependent Mean**	0.03952	**Adj R-Sq**	0.8545
**Coeff Var**	22.96071		

Parameter Estimates						
**Variable**	**Label**	**DF**	**Parameter Estimate**	**Standard Error**	**t Value**	**Pr > |t|**
**Intercept**	Intercept	1	0.00517	0.00522	0.99	0.3473
**ER_UTM_dia**	ER_UTM_dia	1	0.86907	0.11244	7.73	<.0001

The REG Procedure
Model: MODEL1
Dependent Variable: Pred Predicted

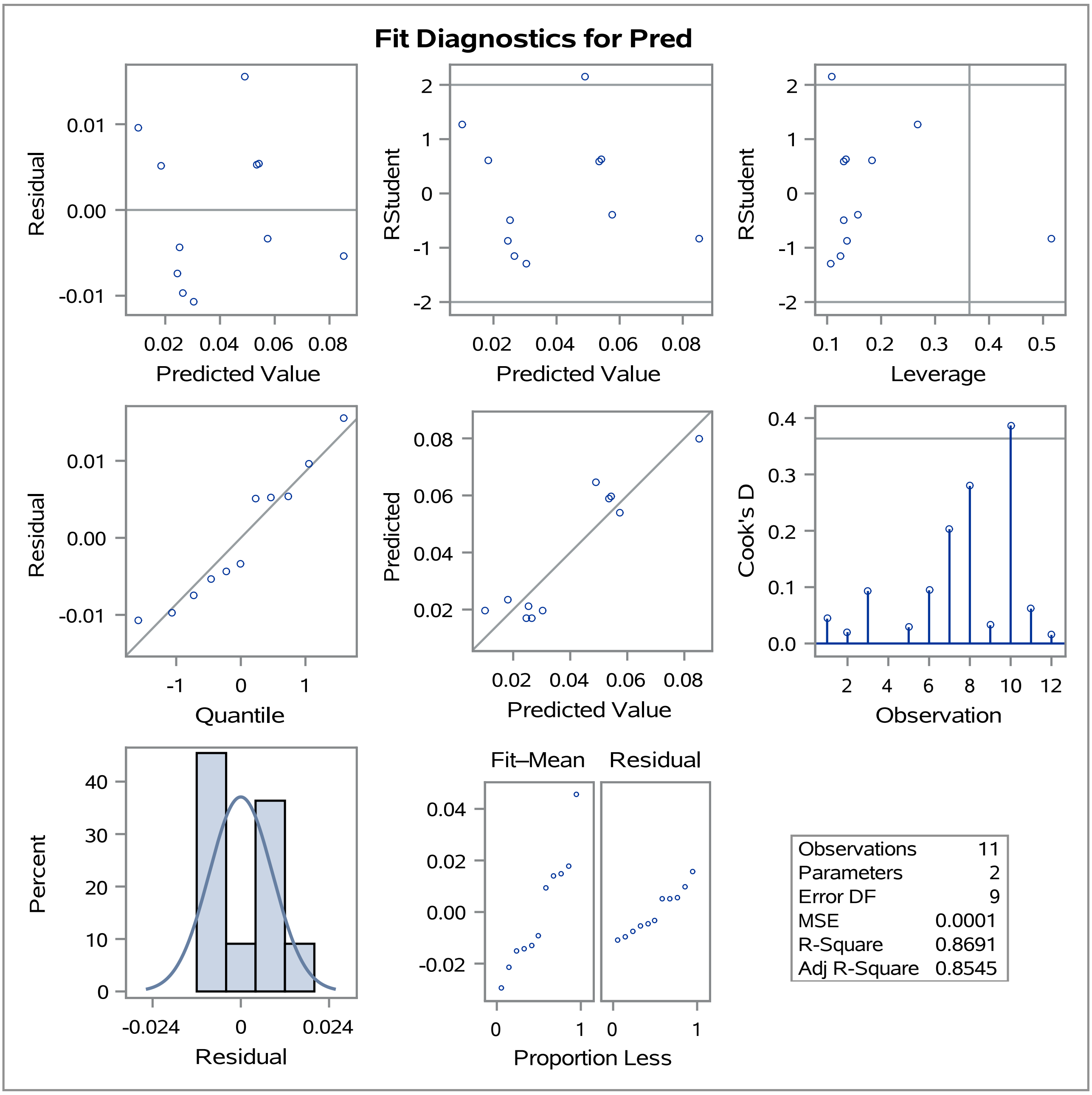

The REG Procedure
Model: MODEL1
Dependent Variable: Pred Predicted

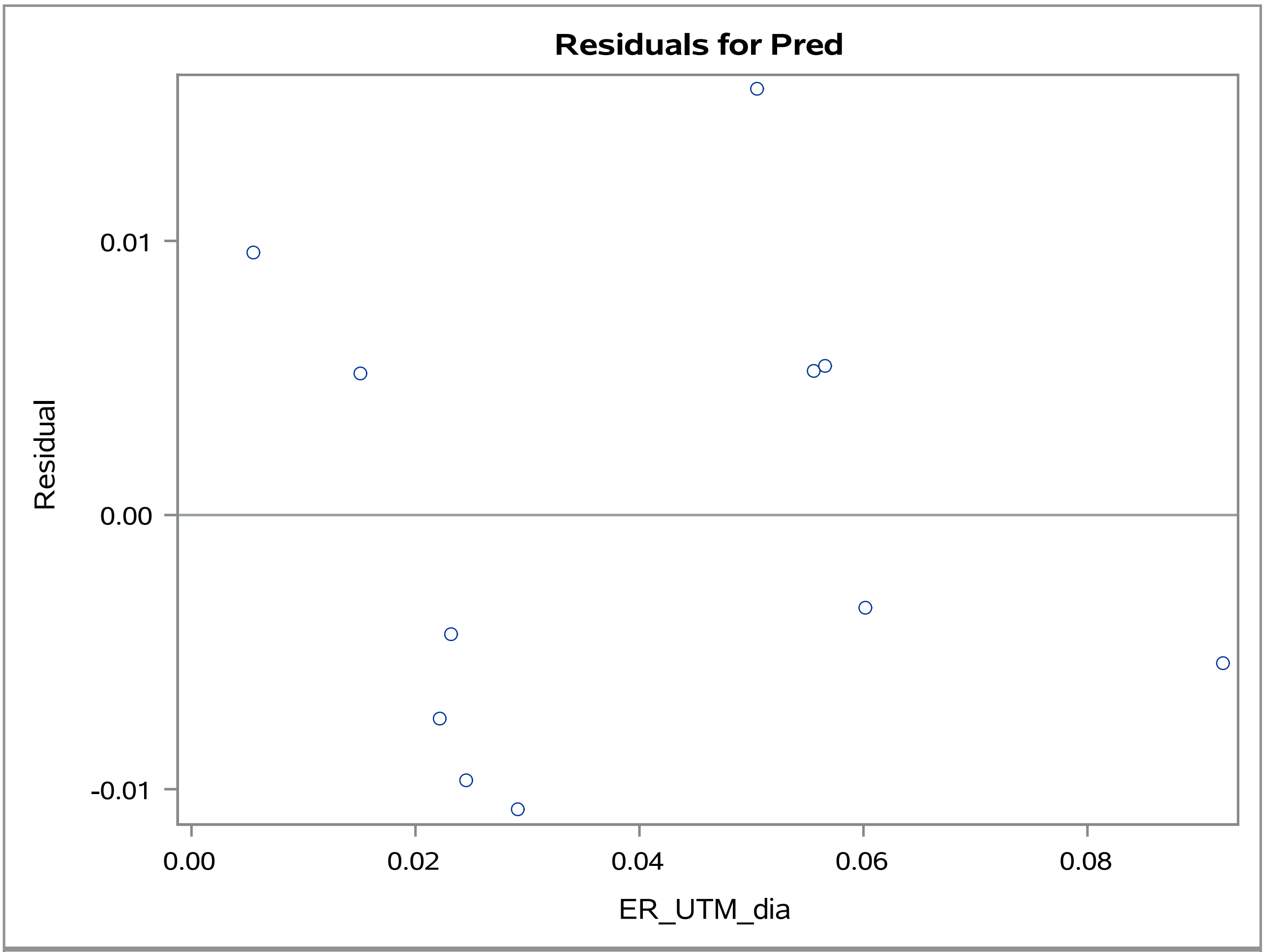

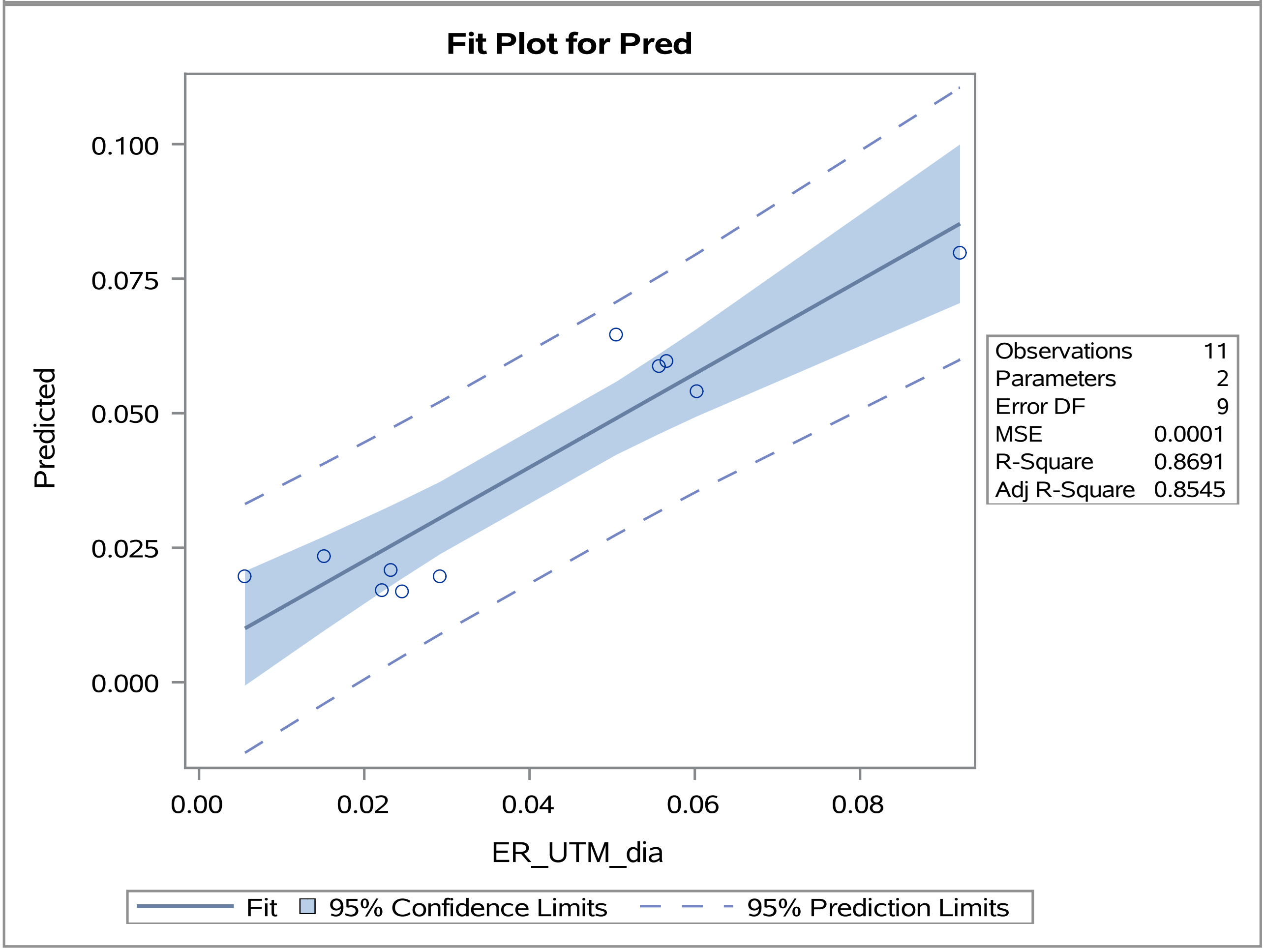

The MEANS Procedure

Analysis Variable : MSE				
N	Mean	Std Dev	Minimum	Maximum
11	0.000077513	0.000073161	4.9512496E-6	0.000199087
