# Supplementary material for: Energy requirements for pregnant dairy cows
Source: PLoS One. 2020 Jul 7;15(7):e0235619. doi: 10.1371/journal.pone.0235619 (PMC7340303; doi:10.1371/journal.pone.0235619)
Supplement: S4 File — (PDF) [file pone.0235619.s004.pdf]

The CONTENTS Procedure

**Data Set Name**	WORK.IMPORT	**Observations**	340
**Member Type**	DATA	**Variables**	132
**Engine**	V9	**Indexes**	0
**Created**	06/03/2020 17:45:54	**Observation Length**	1080
**Last Modified**	06/03/2020 17:45:54	**Deleted Observations**	0
**Protection**		**Compressed**	NO
**Data Set Type**		**Sorted**	NO
**Label**			
**Data Representation**	SOLARIS_X86_64, LINUX_X86_64, ALPHA_TRU64, LINUX_IA64		
**Encoding**	utf-8 Unicode (UTF-8)		

Engine/Host Dependent Information	
**Data Set Page Size**	90112
**Number of Data Set Pages**	5
**First Data Page**	1
**Max Obs per Page**	83
**Obs in First Data Page**	65
**Number of Data Set Repairs**	0
**Filename**	/tmp/SAS_work184800000A1A_localhost.localdomain/SAS_work148F00000A1A_localhost.localdomain/import.sas7bdat
**Release Created**	9.0401M6
**Host Created**	Linux
**Inode Number**	671913
**Access Permission**	rw-rw-r--
**Owner Name**	sasdemo
**File Size**	528KB
**File Size (bytes)**	540672

The CONTENTS Procedure

Alphabetic List of Variables and Attributes						
#	Variable	Type	Len	Format	Informat	Label
7	AL	Char	3	$3.	$3.	AL
104	ANIMAL	Num	8	BEST.		ANIMAL
19	CARi_EE	Num	8	15.2		CARi_EE
21	CARi_MCAL	Num	8	15.2		CARi_MCAL
13	CARi_MN	Num	8	BEST.		CARi_MN
15	CARi_MS	Num	8	15.2		CARi_MS
17	CARi_PB	Num	8	15.2		CARi_PB
113	CCVZf	Num	8	15.2		CCVZf
114	CCVZi	Num	8	15.2		CCVZi
106	CEM_UTM	Num	8	COMMA15.4		CEM_UTM
105	CEM_dia	Num	8	COMMA15.4		CEM_dia
35	CTMEDIO	Num	8	15.2		CTMEDIO
45	CTRET	Num	8	15.2		CTRET
42	CT_EE	Num	8	15.2		CT_EE
44	CT_MCAL	Num	8	15.2		CT_MCAL
34	CT_MN	Num	8	15.2		CT_MN
38	CT_MS	Num	8	15.2		CT_MS
40	CT_PB	Num	8	15.2		CT_PB
41	CTi_EE	Num	8	15.2		CTi_EE
43	CTi_MCAL	Num	8	15.2		CTi_MCAL
33	CTi_MN	Num	8	15.2		CTi_MN
37	CTi_MS	Num	8	15.2		CTi_MS
39	CTi_PB	Num	8	15.2		CTi_PB
20	Carca__a_EE	Num	8	15.2		Carcaça_EE
22	Carca__a_MCAL	Num	8	15.2		Carcaça_MCAL
14	Carca__a_MN	Num	8	15.2		Carcaça_MN
16	Carca__a_MS	Num	8	15.2		Carcaça_MS
18	Carca__a_PB	Num	8	15.2		Carcaça_PB
2	DC	Num	8	BEST.		DC
8	DIETA	Num	8	BEST.		DIETA
93	EBG	Num	8	15.2		EBG
95	EBGc	Num	8	15.2		EBGc
117	ELM	Num	8	COMMA15.3		ELM
129	ELM_nrc_	Num	8	15.2		ELM(nrc)
118	EMM	Num	8	COMMA15.3		EMM
120	EMM_1	Char	24	$24.	$24.	EMM_1

The CONTENTS Procedure

Alphabetic List of Variables and Attributes						
#	Variable	Type	Len	Format	Informat	Label
130	EMM_nrc_	Num	8	15.2		EMM(nrc)
108	ER	Num	8	15.2		ER
109	ERDIA	Num	8	15.2		ERDIA
110	ER_UTM	Num	8	COMMA15.3		ER UTM
111	ER_UTM_dia	Num	8	COMMA15.3		ER_UTM_dia
103	EXPD	Num	8	COMMA15.4		EXPD
102	EXPS	Num	8	COMMA15.4		EXPS
101	EXP_DUPLO	Num	8	15.2		EXP DUPLO
100	EXP_SIMPLES	Num	8	15.2		EXP SIMPLES
122	Elgest	Char	18	$18.	$18.	Elgest
127	Elm_1	Num	8	15.2		Elm_1
131	Elm_brcorte_	Num	8	BEST.		Elm(brcorte)
128	Emm_2	Num	8	15.2		Emm_2
132	Emm_brcorte_	Num	8	BEST.		Emm(brcorte)
83	Feto_EE	Num	8	15.2		Feto_EE
84	Feto_MCAL	Num	8	15.2		Feto_MCAL
80	Feto_MN	Num	8	15.2		Feto_MN
81	Feto_MS	Num	8	15.2		Feto_MS
82	Feto_PB	Num	8	15.2		Feto_PB
36	GCT	Num	8	15.2		GCT
90	GESTMCAL	Num	8	15.2		GESTMCAL
87	GESTMCALi	Num	8	15.2		GESTMCALi
55	GM_EE	Num	8	15.2		GM_EE
57	GM_MCAL	Num	8	15.2		GM_MCAL
49	GM_MN	Num	8	15.2		GM_MN
51	GM_MS	Num	8	15.2		GM_MS
53	GM_PB	Num	8	15.2		GM_PB
54	GMi_EE	Num	8	15.2		GMi_EE
56	GMi_MCAL	Num	8	15.2		GMi_MCAL
48	GMi_MN	Num	8	15.2		GMi_MN
50	GMi_MS	Num	8	15.2		GMi_MS
52	GMi_PB	Num	8	15.2		GMi_PB
9	GPCVZ	Num	8	BEST.		GPCVZ
126	GPCZ	Num	8	15.2		GPCZ
3	GT	Num	8	BEST.		GT
78	LA_EE	Num	8	15.2		LA_EE

The CONTENTS Procedure

Alphabetic List of Variables and Attributes						
#	Variable	Type	Len	Format	Informat	Label
79	LA_MCAL	Num	8	15.2		LA_MCAL
75	LA_MN	Num	8	15.2		LA_MN
76	LA_MS	Num	8	15.2		LA_MS
77	LA_PB	Num	8	15.2		LA_PB
10	LGPCVZ	Num	8	BEST.		LGPCVZ
46	LOGCTRET	Num	8	BEST.		LOGCTRET
94	LOGEBGUTM	Num	8	COMMA15.4		LOGEBGUTM
112	LOGER	Num	8	COMMA15.3		LOGER
47	LOGGCT	Num	8	15.2		LOGGCT
121	MEG	Char	18	$18.	$18.	MEG
123	MEGEST	Num	8	BEST.		MEGEST
91	McalGESTopen	Num	8	15.2		McalGESTopen
92	McalGESTpreg	Num	8	15.2		McalGESTpreg
29	NCARi_EE	Num	8	15.2		NCARi_EE
31	NCARi_MCAL	Num	8	15.2		NCARi_MCAL
23	NCARi_MN	Num	8	15.2		NCARi_MN
25	NCARi_MS	Num	8	15.2		NCARi_MS
27	NCARi_PB	Num	8	15.2		NCARi_PB
30	NCarca__a_EE	Num	8	15.2		NCarcaça_EE
32	NCarca__a_MCAL	Num	8	15.2		NCarcaça_MCAL
24	NCarca__a_MN	Num	8	15.2		NCarcaça_MN
26	NCarca__a_MS	Num	8	15.2		NCarcaça_MS
28	NCarca__a_PB	Num	8	15.2		NCarcaça_PB
116	PC	Num	8	COMMA15.3		PC
11	PCVZf	Num	8	15.2		PCVZf
12	PCVZi	Num	8	BEST.		PCVZi
115	PC_UTM	Num	8	COMMA15.3		PC_UTM
73	Placenta_EE	Num	8	15.2		Placenta_EE
74	Placenta_MCAL	Num	8	15.2		Placenta_MCAL
70	Placenta_MN	Num	8	15.2		Placenta_MN
71	Placenta_MS	Num	8	15.2		Placenta_MS
72	Placenta_PB	Num	8	15.2		Placenta_PB
97	SALDO	Num	8	15.2		SALDO
98	SALDO_D	Num	8	15.2		SALDO/D
58	SALDO_GM	Num	8	15.2		SALDO GM
99	SALDO_UG	Num	8	15.2		SALDO UG

The CONTENTS Procedure

Alphabetic List of Variables and Attributes						
#	Variable	Type	Len	Format	Informat	Label
69	SALDO_UTERO	Num	8	15.2		SALDO UTERO
4	ST	Char	1	$1.	$1.	ST
5	STM	Num	8	BEST.		STM
6	TG	Num	8	BEST.		TG
65	UTEROi_EE	Num	8	15.2		UTEROi_EE
67	UTEROi_MCAL	Num	8	15.2		UTEROi_MCAL
59	UTEROi_MN	Num	8	15.2		UTEROi_MN
61	UTEROi_MS	Num	8	15.2		UTEROi_MS
63	UTEROi_PB	Num	8	15.2		UTEROi_PB
107	UTM	Num	8	15.2		UTM
125	UTM_1	Num	8	15.2		UTM_1
96	UTMc	Num	8	COMMA15.4		UTMc
66	Utero_EE	Num	8	15.2		Utero_EE
68	Utero_MCAL	Num	8	15.2		Utero_MCAL
60	Utero_MN	Num	8	15.2		Utero_MN
62	Utero_MS	Num	8	15.2		Utero_MS
64	Utero_PB	Num	8	15.2		Utero_PB
1	anim	Num	8	BEST.		anim
88	fEBWopen	Num	8	15.2		fEBWopen
86	kgGEST	Num	8	15.2		kgGEST
85	kgGESTopen	Num	8	15.2		kgGESTopen
89	kgGESTpreg	Num	8	15.2		kgGESTpreg
119	km	Num	8	15.2		km
124	kpreg	Num	8	COMMA15.4		kpreg

The NLIN Procedure
Dependent Variable GESTMCAL
Method: Gauss-Newton

Iterative Phase			
Iter	a	b	Sum of Squares
0	0.000040	2.0200	1396.0
1	0.000026	2.1095	1093.9
2	0.000018	2.2271	465.2
3	0.000021	2.2166	399.7
4	0.000022	2.2175	398.8
5	0.000022	2.2174	398.8

NOTE: Convergence criterion met.

Estimation Summary	
Method	Gauss-Newton
Iterations	5
Subiterations	2
Average Subiterations	0.4
R	1.688E-7
PPC(a)	5.249E-8
RPC(a)	0.000072
Object	1.382E-8
Objective	398.8041
Observations Read	18
Observations Used	17
Observations Missing	1

Note: An intercept was not specified for this model.

Source	DF	Sum of Squares	Mean Square	F Value	Approx Pr > F
Model	2	4879.5	2439.8	91.77	<.0001
Error	15	398.8	26.5869		
Uncorrected Total	17	5278.3			

Parameter	Estimate	Approx Std Error	Approximate 95% Confidence Limits	
a	0.000022	0.000066	-0.00012	0.000162
b	2.2174	0.4939	1.1648	3.2701

The NLIN Procedure

Approximate Correlation Matrix		
	a	b
a	1.0000000	-0.9997071
b	-0.9997071	1.0000000

The REG Procedure
Model: MODEL1
Dependent Variable: pred

Analysis of Variance					
Source	DF	Sum of Squares	Mean Square	F Value	Pr > F
**Model**	1	313.73335	313.73335	20.44	0.0004
**Error**	15	230.23247	15.34883		
**Corrected Total**	16	543.96582			

**Root MSE**	3.91776	**R-Square**	0.5768
**Dependent Mean**	15.96975	**Adj R-Sq**	0.5485
**Coeff Var**	24.53238		

Parameter Estimates						
Variable	Label	DF	Parameter Estimate	Standard Error	t Value	Pr > |t|
**Intercept**	Intercept	1	6.75417	2.24895	3.00	0.0089
**GESTMCAL**	GESTMCAL	1	0.57703	0.12763	4.52	0.0004

The REG Procedure
Model: MODEL1
Dependent Variable: pred

Fit Diagnostics for pred

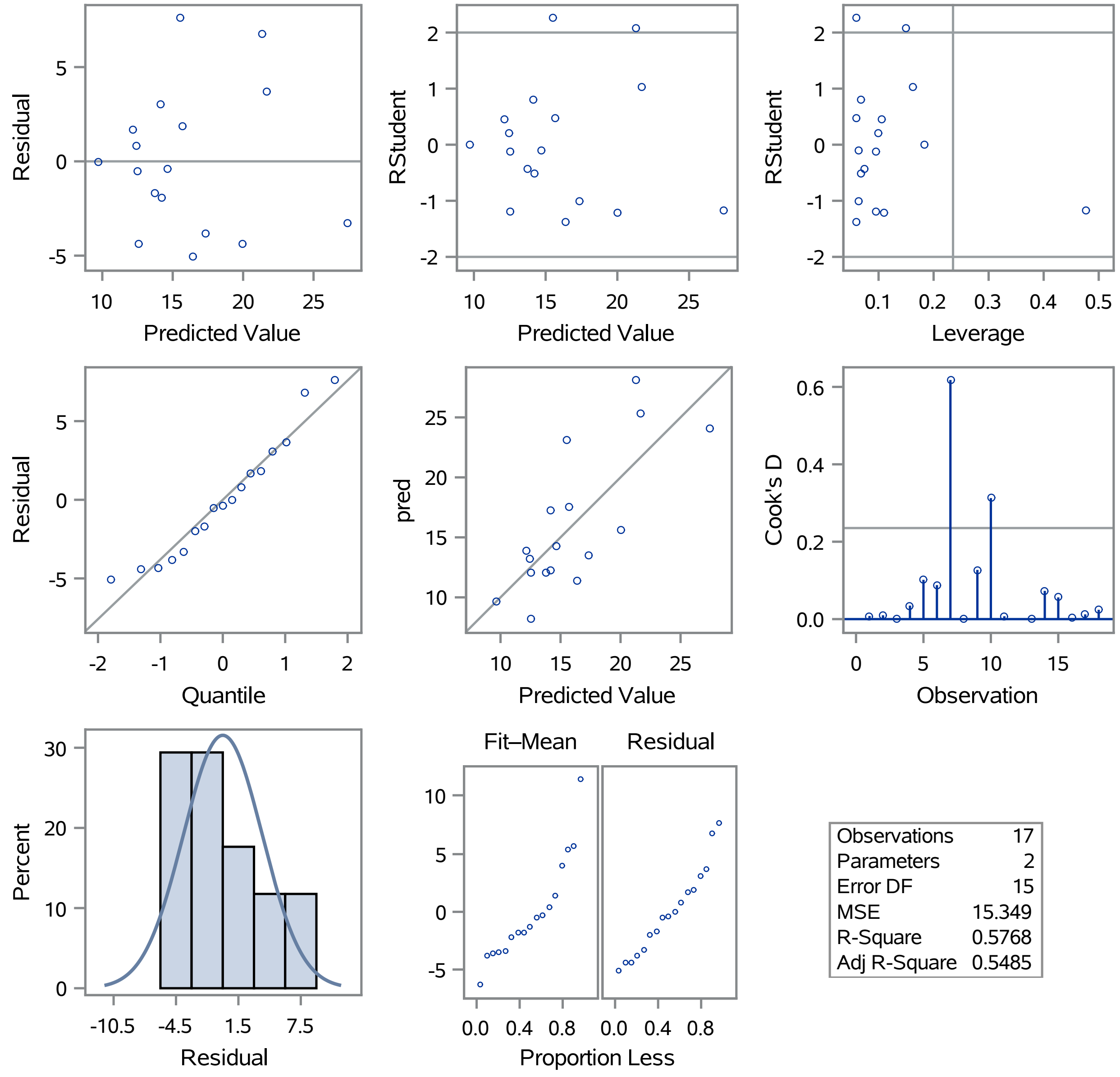

Observations	17
Parameters	2
Error DF	15
MSE	15.349
R-Square	0.5768
Adj R-Square	0.5485

The REG Procedure
Model: MODEL1
Dependent Variable: pred

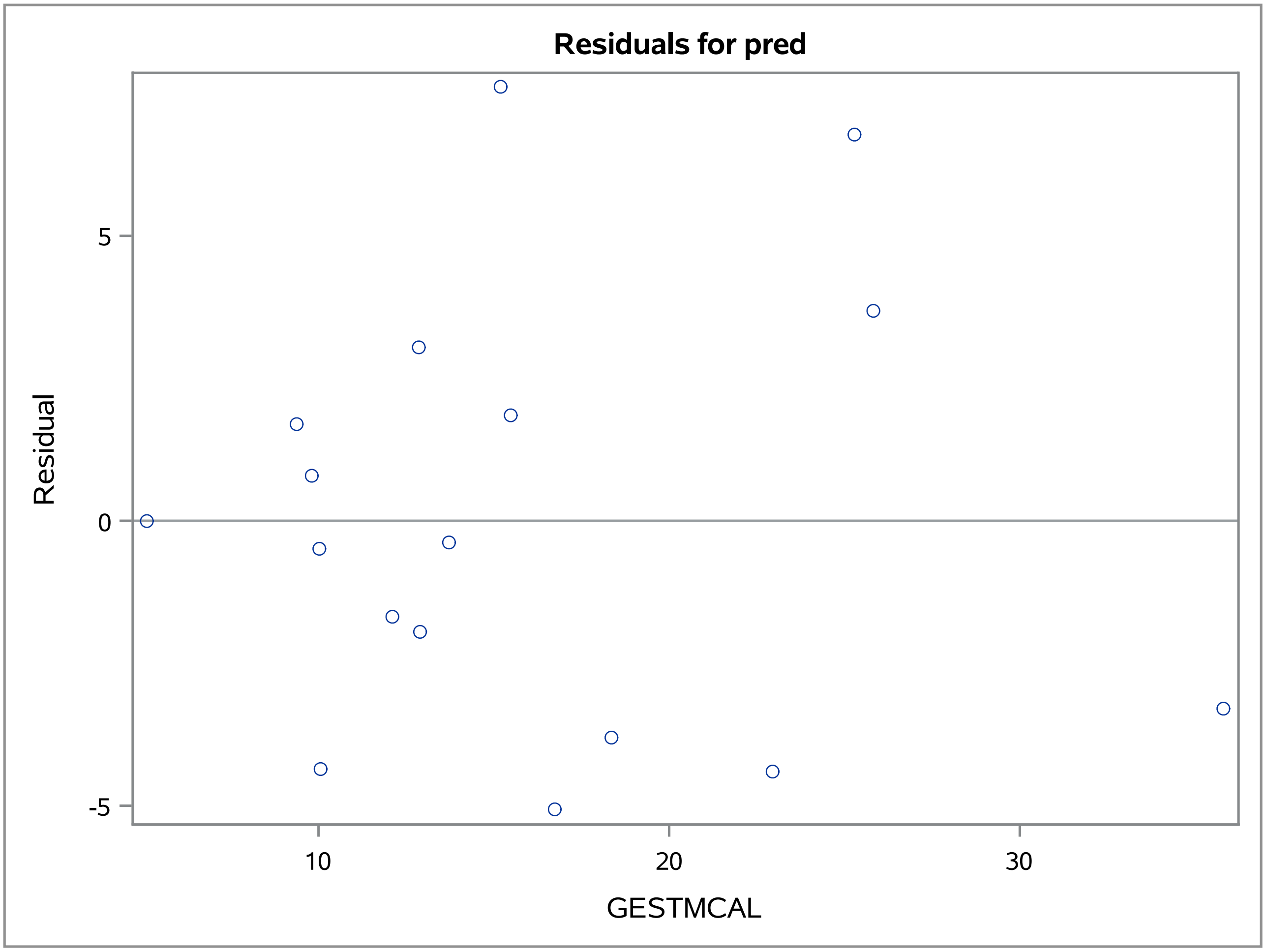

The REG Procedure
Model: MODEL1
Dependent Variable: pred

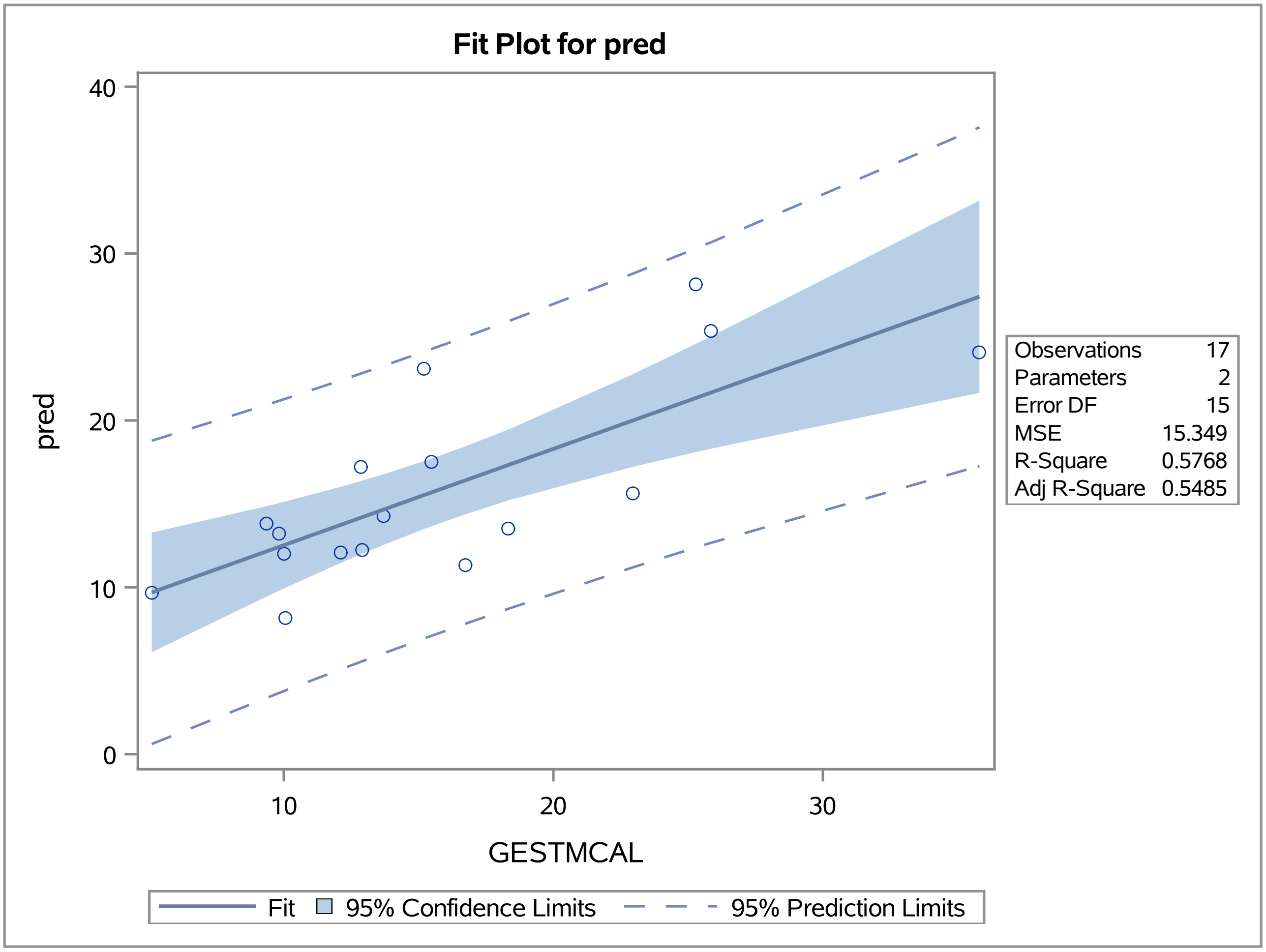

The MEANS Procedure

Analysis Variable : MSE				
N	Mean	Std Dev	Minimum	Maximum
17	23.4590627	34.5457785	0.0031314	136.7694281

The NLIN Procedure
Dependent Variable kgGEST
Method: Gauss-Newton

Iterative Phase			
Iter	a	b	Sum of Squares
0	0.000040	2.0200	359.6
1	0.000073	1.8169	59.5803
2	0.000083	1.7956	59.3133
3	0.000101	1.7650	59.2297
4	0.000123	1.7325	58.8651
5	0.000171	1.6797	58.8512
6	0.000207	1.6586	55.6763
7	0.000213	1.6578	55.3856
8	0.000213	1.6577	55.3854

NOTE: Convergence criterion met.

Estimation Summary	
Method	Gauss-Newton
Iterations	8
Subiterations	5
Average Subiterations	0.625
R	6.793E-6
PPC(a)	6.159E-6
RPC(a)	0.000687
Object	3.056E-6
Objective	55.38539
Observations Read	18
Observations Used	18
Observations Missing	0

Note: An intercept was not specified for this model.

Source	DF	Sum of Squares	Mean Square	F Value	Approx Pr > F
Model	2	528.5	264.3	76.34	<.0001
Error	16	55.3854	3.4616		
Uncorrected Total	18	583.9			

The NLIN Procedure

Parameter	Estimate	Approx Std Error	Approximate 95% Confidence Limits	
a	0.000213	0.000703	-0.00128	0.00170
b	1.6577	0.5359	0.5216	2.7939

Approximate Correlation Matrix		
	a	b
a	1.0000000	-0.9996996
b	-0.9996996	1.0000000

The REG Procedure
Model: MODEL1
Dependent Variable: pred

Analysis of Variance					
**Source**	**DF**	**Sum of Squares**	**Mean Square**	**F Value**	**Pr > F**
**Model**	1	12.64862	12.64862	9.50	0.0071
**Error**	16	21.29429	1.33089		
**Corrected Total**	17	33.94291			

**Root MSE**	1.15364	**R-Square**	0.3726
**Dependent Mean**	5.24186	**Adj R-Sq**	0.3334
**Coeff Var**	22.00827		

Parameter Estimates						
**Variable**	**Label**	**DF**	**Parameter Estimate**	**Standard Error**	**t Value**	**Pr > |t|**
**Intercept**	Intercept	1	3.25546	0.69937	4.65	0.0003
**kgGEST**	kgGEST	1	0.37854	0.12279	3.08	0.0071

The REG Procedure
Model: MODEL1
Dependent Variable: pred

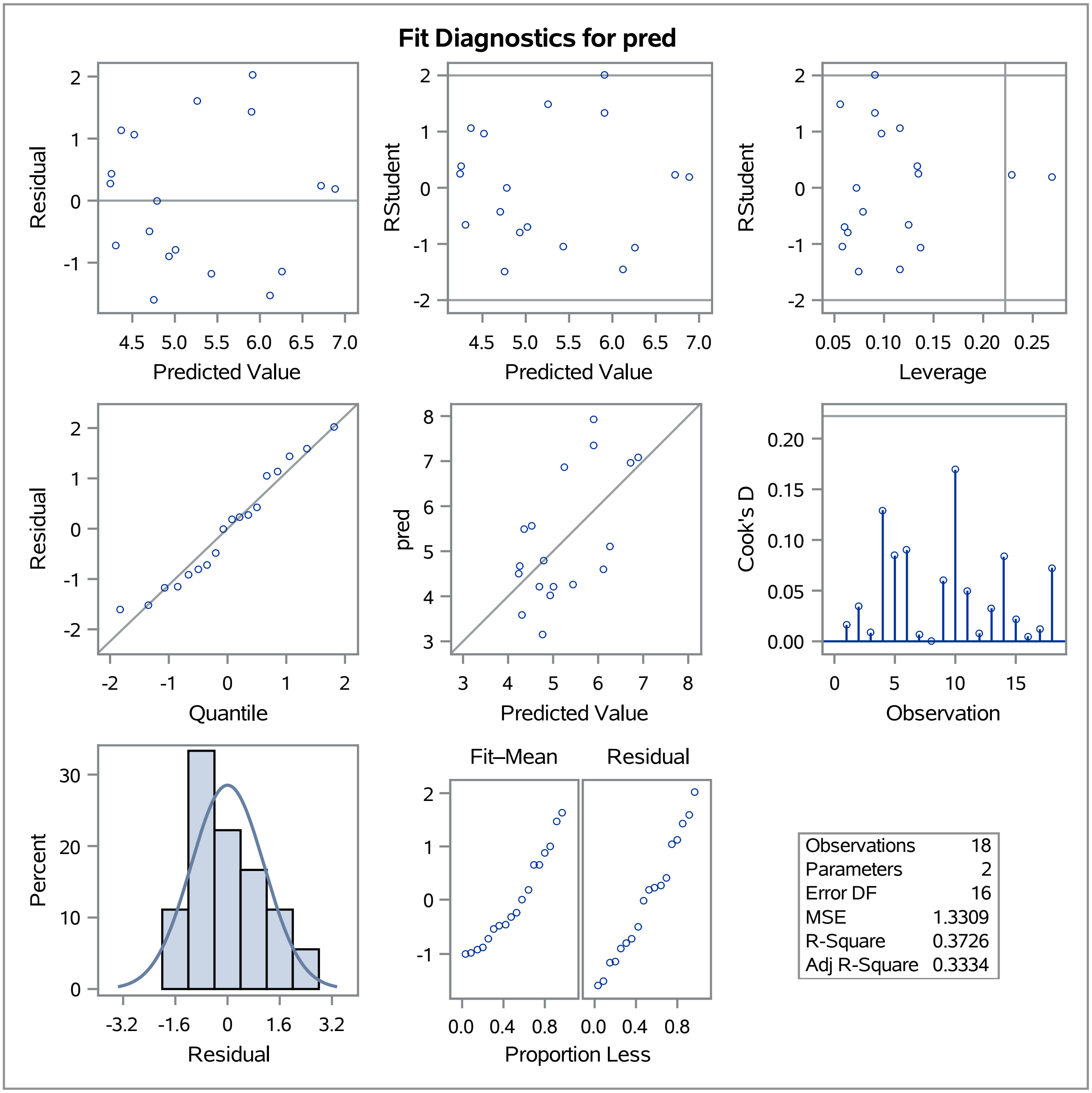

The REG Procedure
Model: MODEL1
Dependent Variable: pred

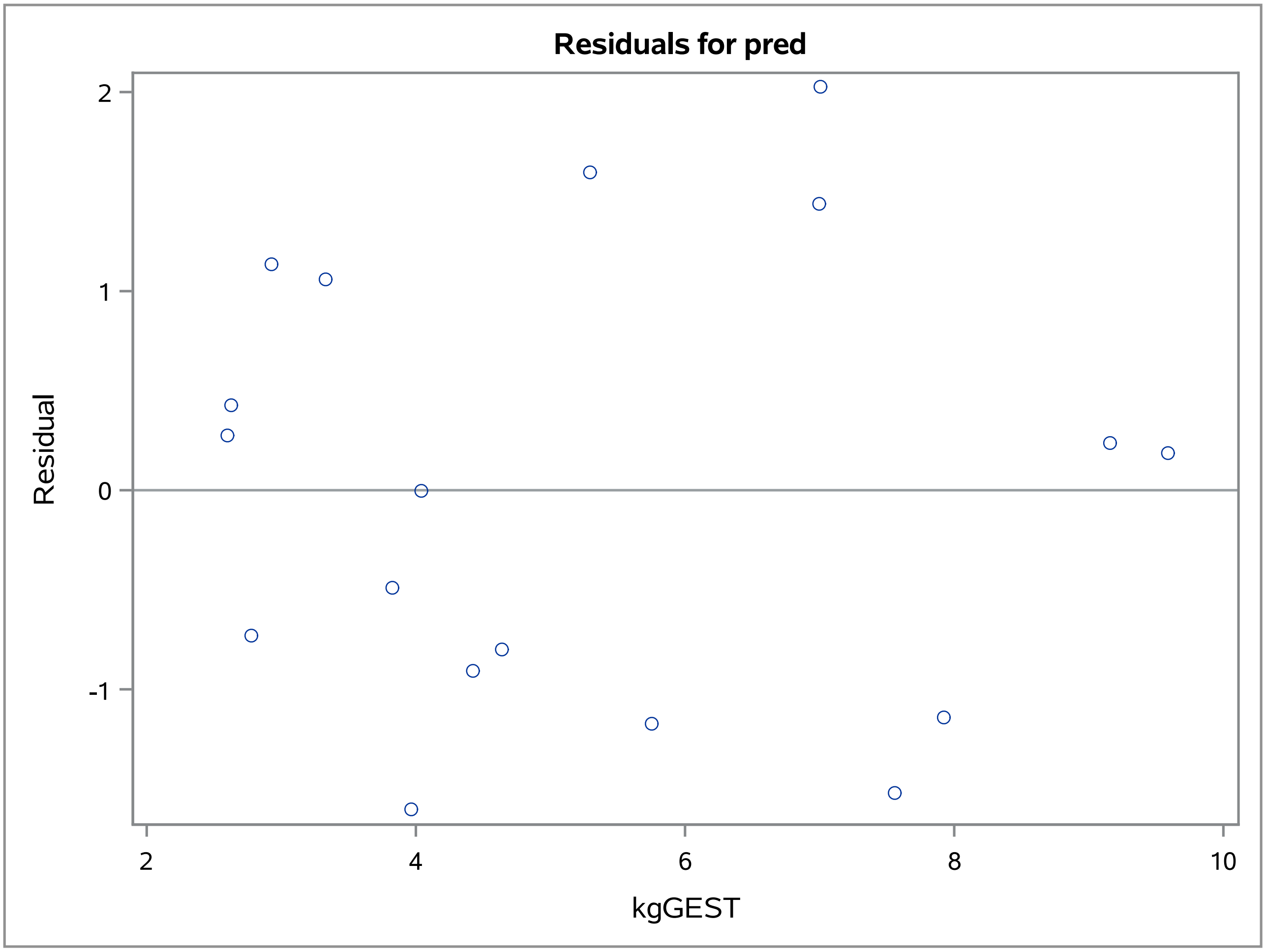

The REG Procedure
Model: MODEL1
Dependent Variable: pred

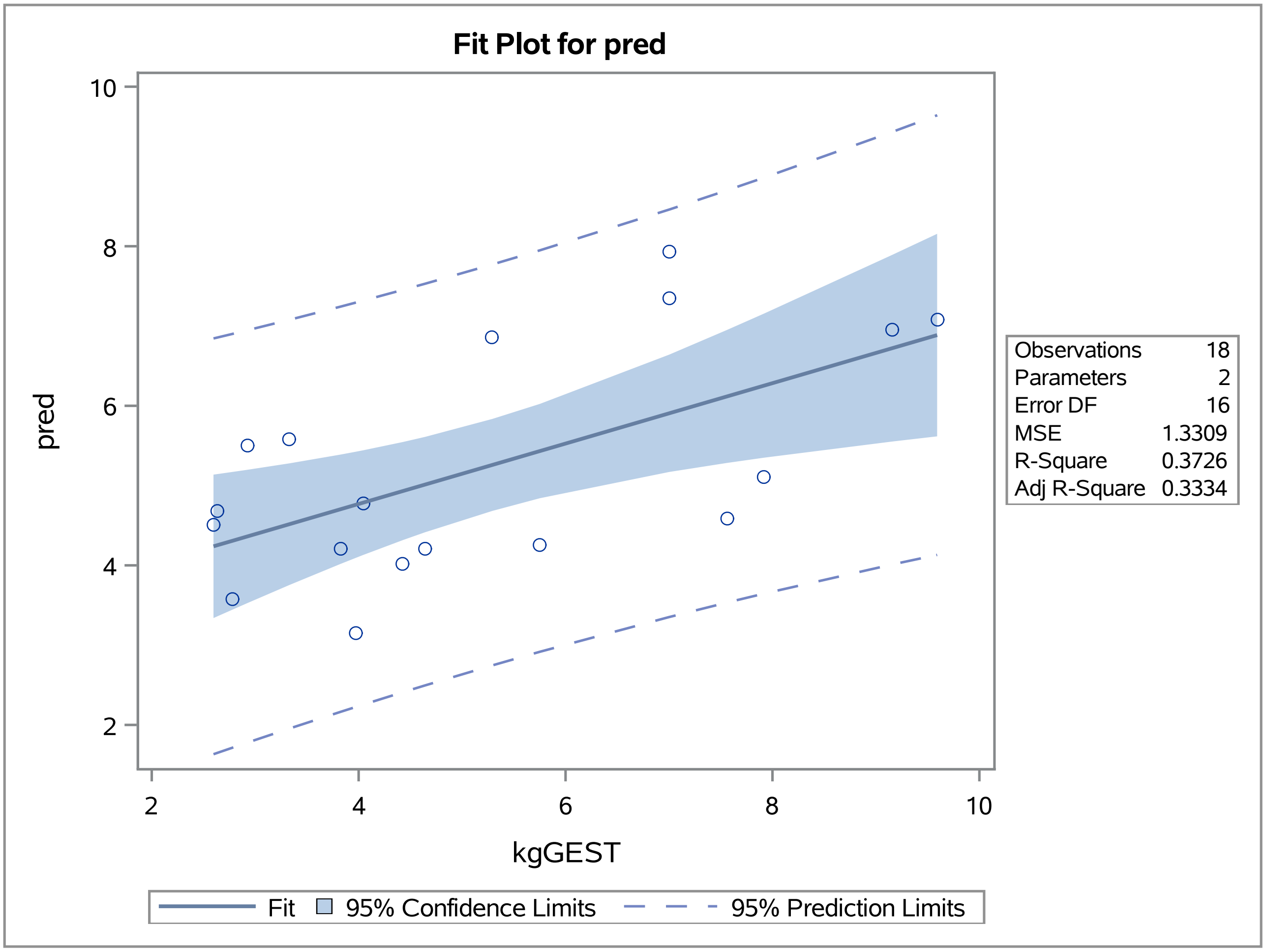

The MEANS Procedure

Analysis Variable : MSE				
N	Mean	Std Dev	Minimum	Maximum
18	3.0769662	2.9315228	0.1158400	8.7880868

The NLIN Procedure
Dependent Variable PC_UTM
Method: Gauss-Newton

Iterative Phase			
**Iter**	**a**	**b**	**Sum of Squares**
**0**	0.0810	3.6000	0.000365
**1**	0.0822	3.5821	0.000331
**2**	0.0822	3.5822	0.000331

Note: Missing values were generated as a result of performing an operation on missing values. Each place is given by (number of times) AT (statement)/(line):(column).

**6 AT**	1/143:17

NOTE: Convergence criterion met.

Estimation Summary	
**Method**	Gauss-Newton
**Iterations**	2
**R**	4.581E-7
**PPC(b)**	8.677E-8
**RPC(b)**	0.000041
**Object**	2.681E-6
**Objective**	0.000331
**Observations Read**	18
**Observations Used**	9
**Observations Missing**	9

Note: An intercept was not specified for this model.

Source	DF	Sum of Squares	Mean Square	F Value	Approx Pr > F
**Model**	2	0.3196	0.1598	3374.87	<.0001
**Error**	7	0.000331	0.000047		
**Uncorrected Total**	9	0.3199			

Parameter	Estimate	Approx Std Error	Approximate 95% Confidence Limits	
**a**	0.0822	0.00517	0.0700	0.0944
**b**	3.5822	0.2574	2.9736	4.1908

The NLIN Procedure

Approximate Correlation Matrix		
	a	b
a	1.0000000	-0.9811168
b	-0.9811168	1.0000000

The REG Procedure
Model: MODEL1
Dependent Variable: pred

Analysis of Variance					
Source	DF	Sum of Squares	Mean Square	F Value	Pr > F
**Model**	1	0.00907	0.00907	198.04	<.0001
**Error**	7	0.00032061	0.00004580		
**Corrected Total**	8	0.00939			

**Root MSE**	0.00677	**R-Square**	0.9659
**Dependent Mean**	0.18564	**Adj R-Sq**	0.9610
**Coeff Var**	3.64557		

Parameter Estimates						
Variable	Label	DF	Parameter Estimate	Standard Error	t Value	Pr > |t|
**Intercept**	Intercept	1	0.00619	0.01295	0.48	0.6474
**PC_UTM**	PC_UTM	1	0.96665	0.06869	14.07	<.0001

The REG Procedure
Model: MODEL1
Dependent Variable: pred

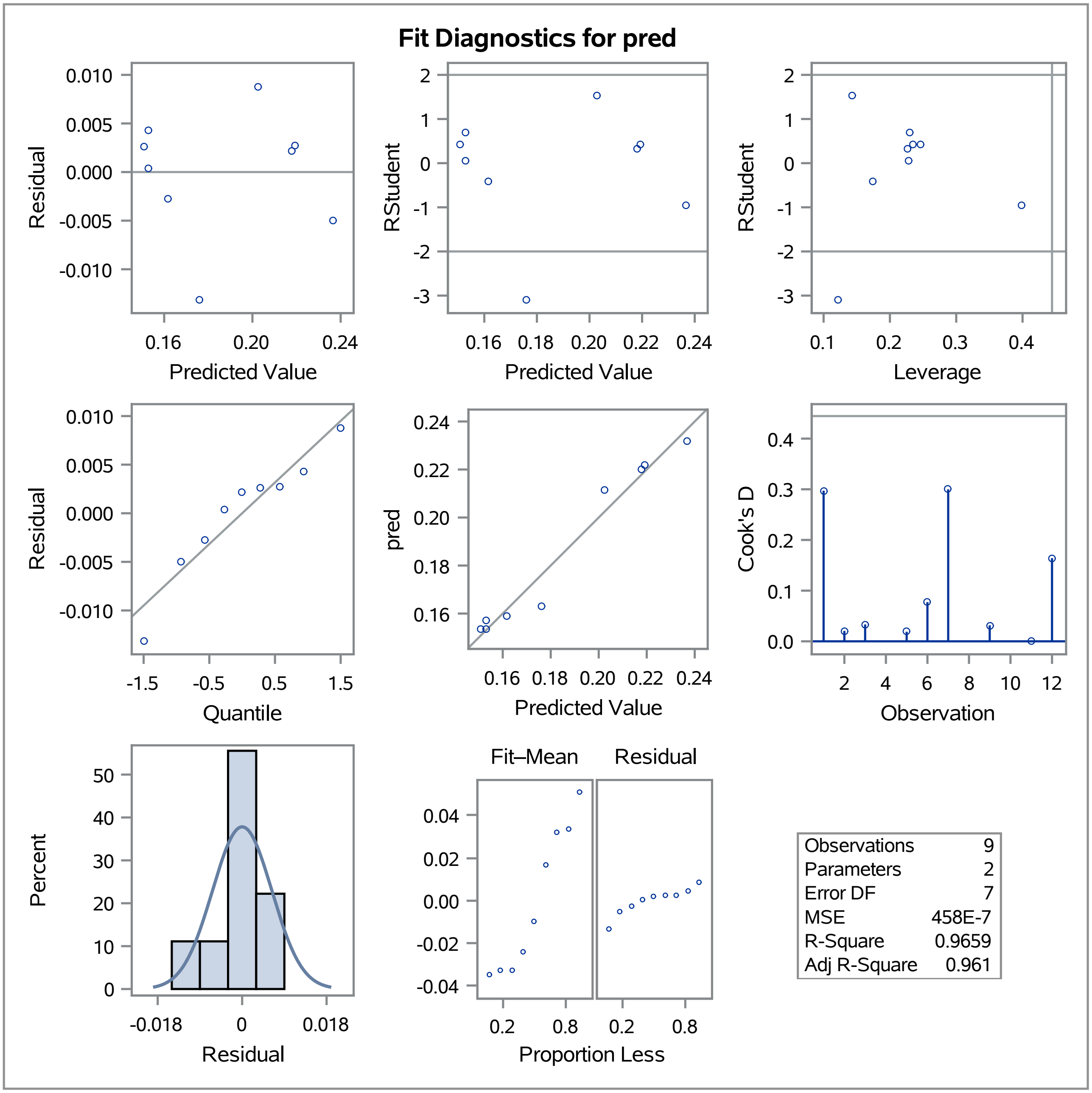

The REG Procedure
Model: MODEL1
Dependent Variable: pred

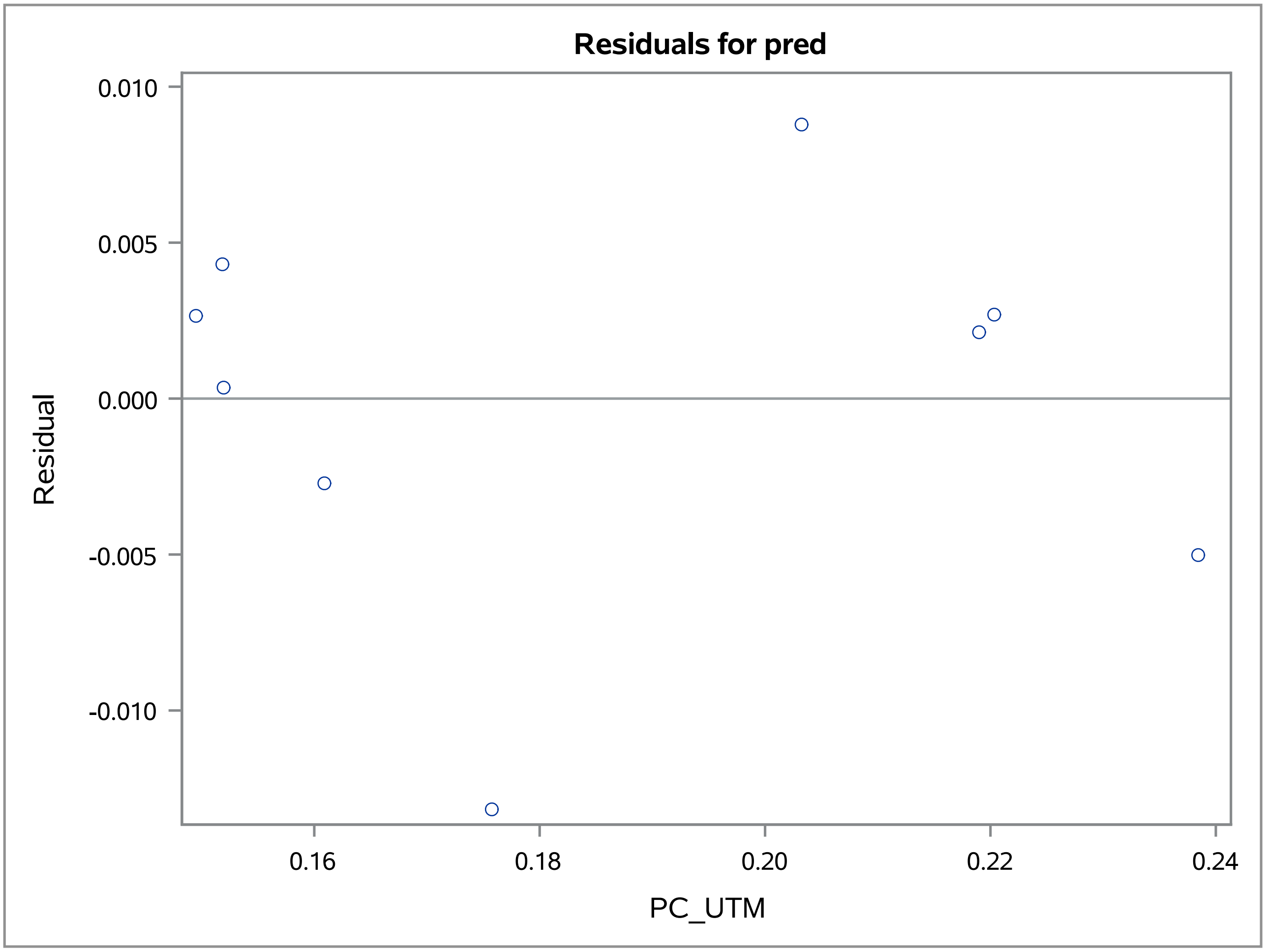

The REG Procedure
Model: MODEL1
Dependent Variable: pred

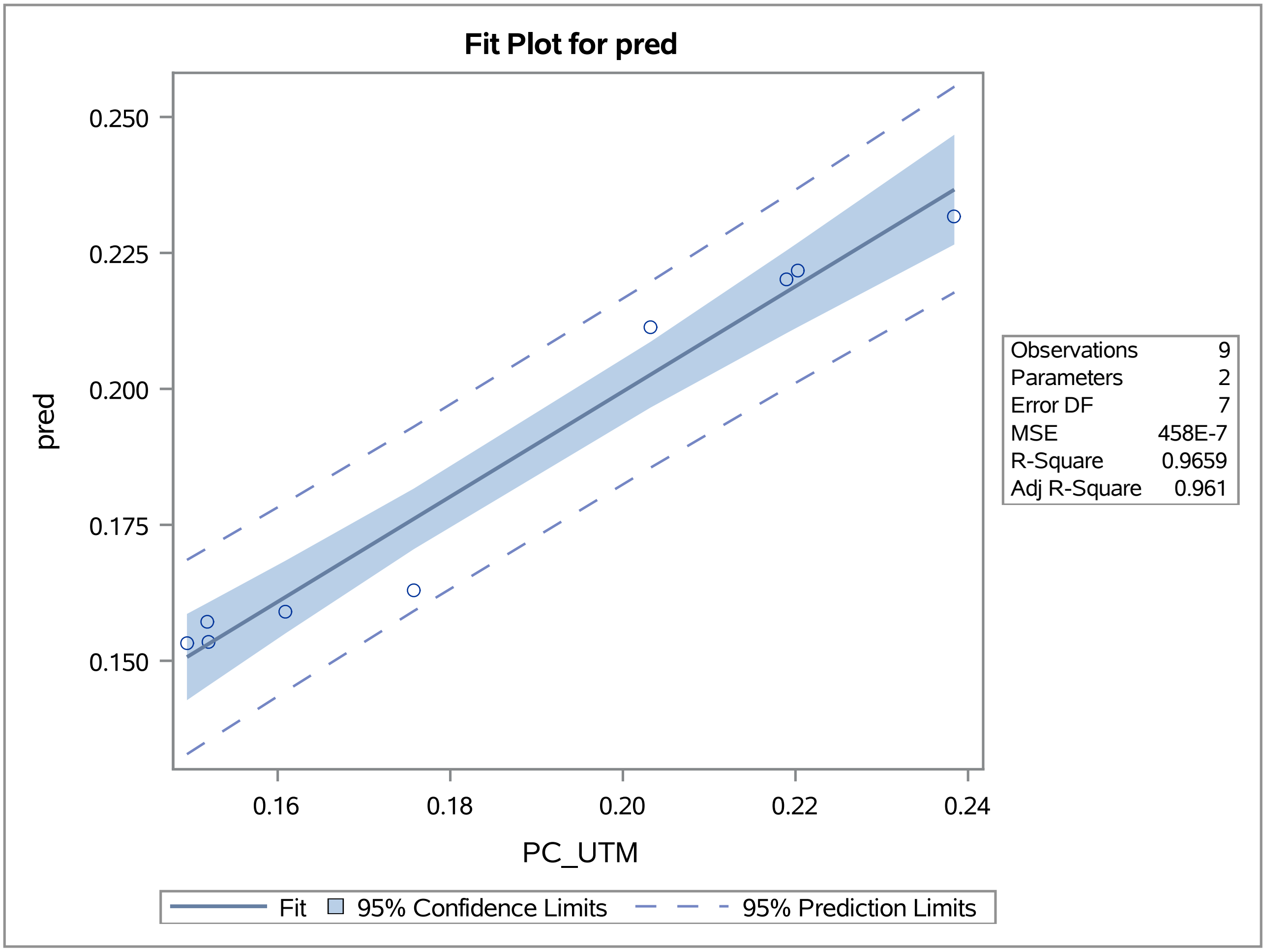

The MEANS Procedure

Analysis Variable : MSE				
N	Mean	Std Dev	Minimum	Maximum
9	0.000036823	0.000053372	1.0531557E-6	0.000165138

The NLIN Procedure
Dependent Variable PC_UTM
Method: Gauss-Newton

Iterative Phase			
**Iter**	**a**	**b**	**Sum of Squares**
**0**	0.0810	3.6000	0.00655
**1**	0.0856	3.4218	0.00616
**2**	0.0857	3.4259	0.00616
**3**	0.0857	3.4258	0.00616

Note: Missing values were generated as a result of performing an operation on missing values. Each place is given by (number of times) AT (statement)/(line):(column).

**280 AT**	1/164:17

NOTE: Convergence criterion met.

Estimation Summary	
**Method**	Gauss-Newton
**Iterations**	3
**R**	3.745E-7
**PPC(b)**	1.102E-7
**RPC(b)**	0.000012
**Object**	1.972E-9
**Objective**	0.00616
**Observations Read**	322
**Observations Used**	42
**Observations Missing**	280

Note: An intercept was not specified for this model.

Source	DF	Sum of Squares	Mean Square	F Value	Approx Pr > F
**Model**	2	1.6113	0.8057	5231.51	<.0001
**Error**	40	0.00616	0.000154		
**Uncorrected Total**	42	1.6175			

Parameter	Estimate	Approx Std Error	Approximate 95% Confidence Limits	
**a**	0.0857	0.00358	0.0784	0.0929
**b**	3.4258	0.1593	3.1038	3.7479

The NLIN Procedure

Approximate Correlation Matrix		
	a	b
a	1.0000000	-0.9722117
b	-0.9722117	1.0000000

The REG Procedure
Model: MODEL1
Dependent Variable: pred

Analysis of Variance					
Source	DF	Sum of Squares	Mean Square	F Value	Pr > F
**Model**	1	0.06719	0.06719	477.13	<.0001
**Error**	40	0.00563	0.00014082		
**Corrected Total**	41	0.07282			

**Root MSE**	0.01187	**R-Square**	0.9226
**Dependent Mean**	0.19139	**Adj R-Sq**	0.9207
**Coeff Var**	6.20026		

Parameter Estimates						
Variable	Label	DF	Parameter Estimate	Standard Error	t Value	Pr > |t|
**Intercept**	Intercept	1	0.01561	0.00825	1.89	0.0658
**PC_UTM**	PC_UTM	1	0.91863	0.04206	21.84	<.0001

The REG Procedure
Model: MODEL1
Dependent Variable: pred

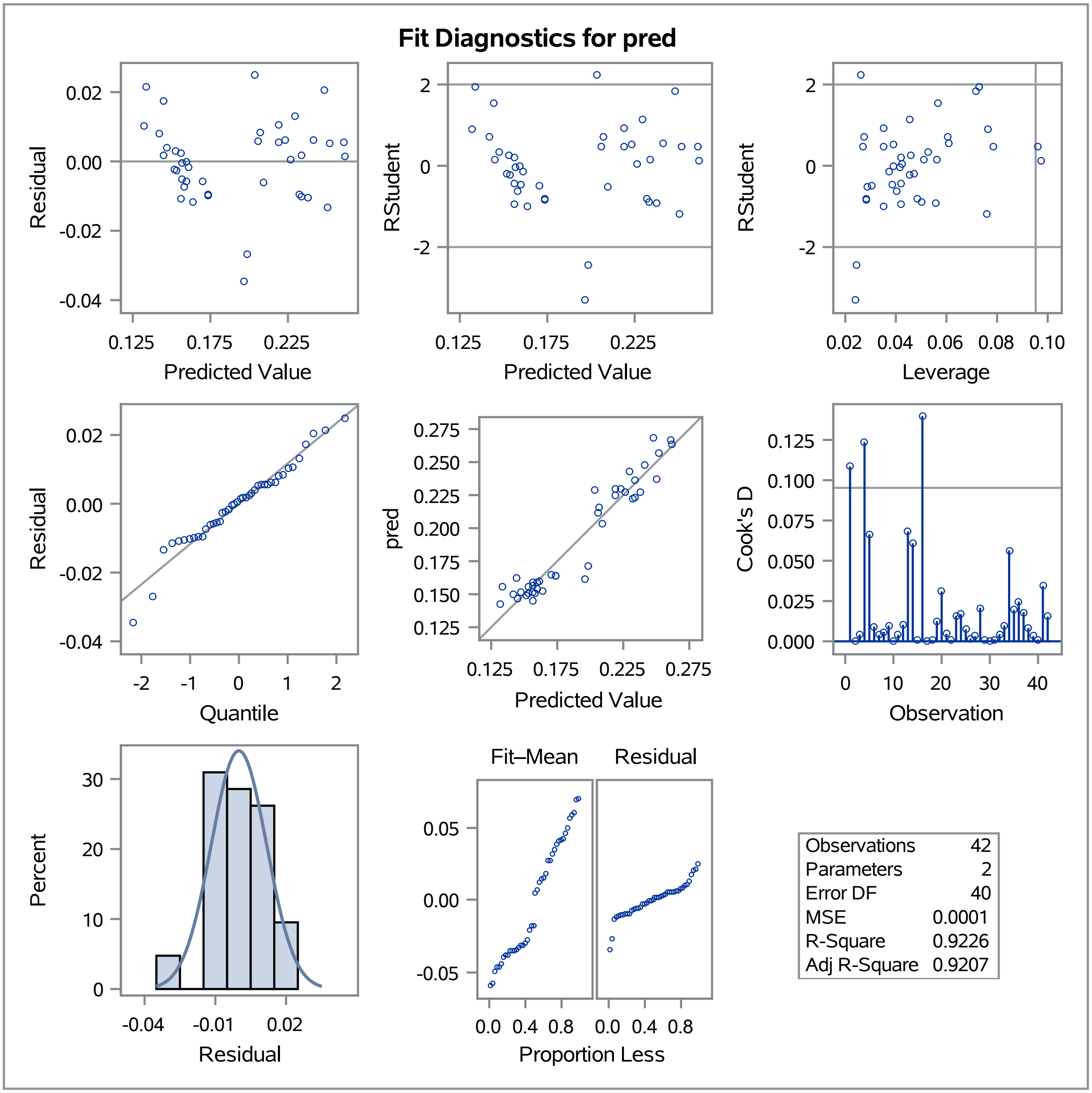

The REG Procedure
Model: MODEL1
Dependent Variable: pred

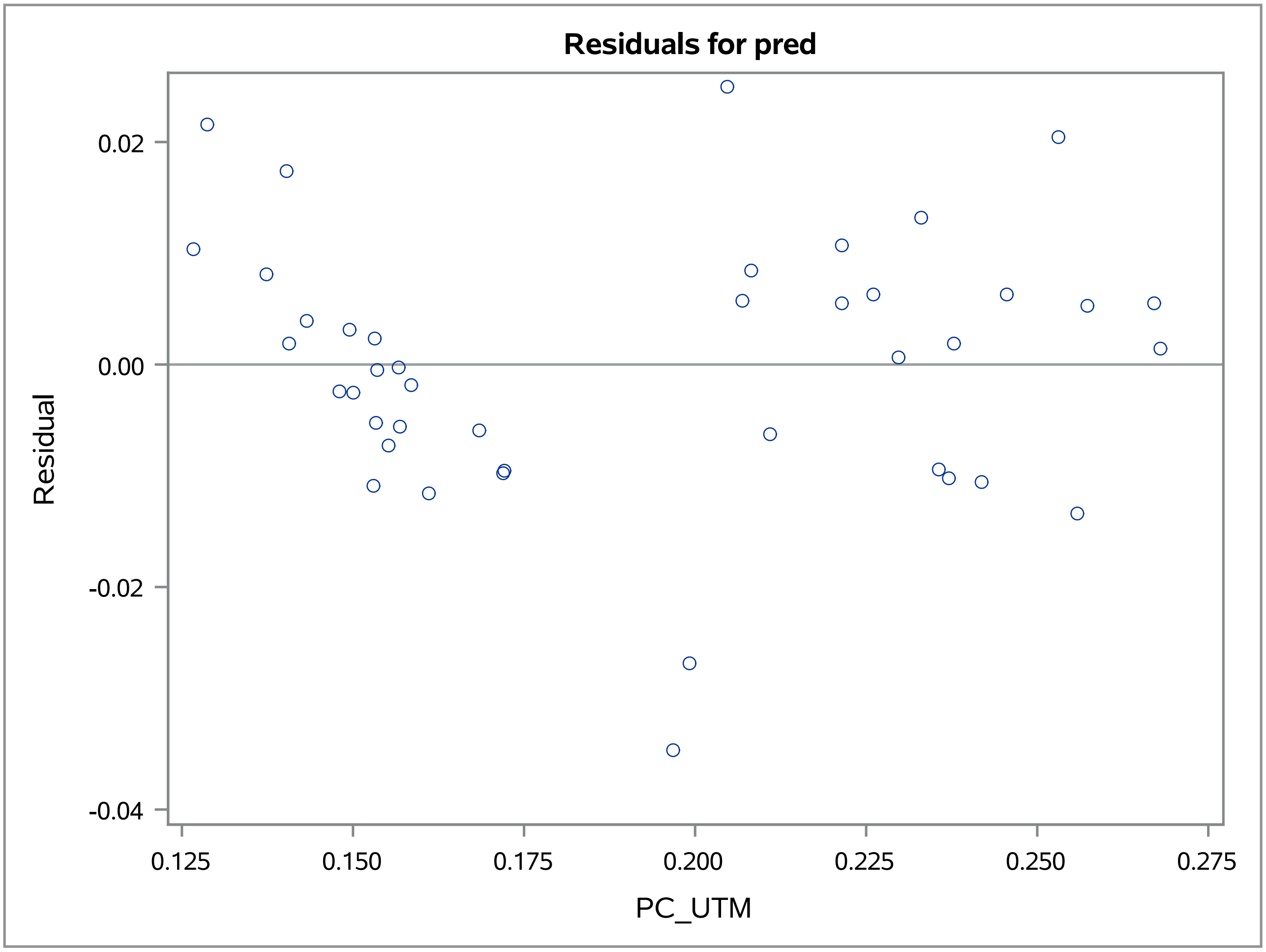

The REG Procedure
Model: MODEL1
Dependent Variable: pred

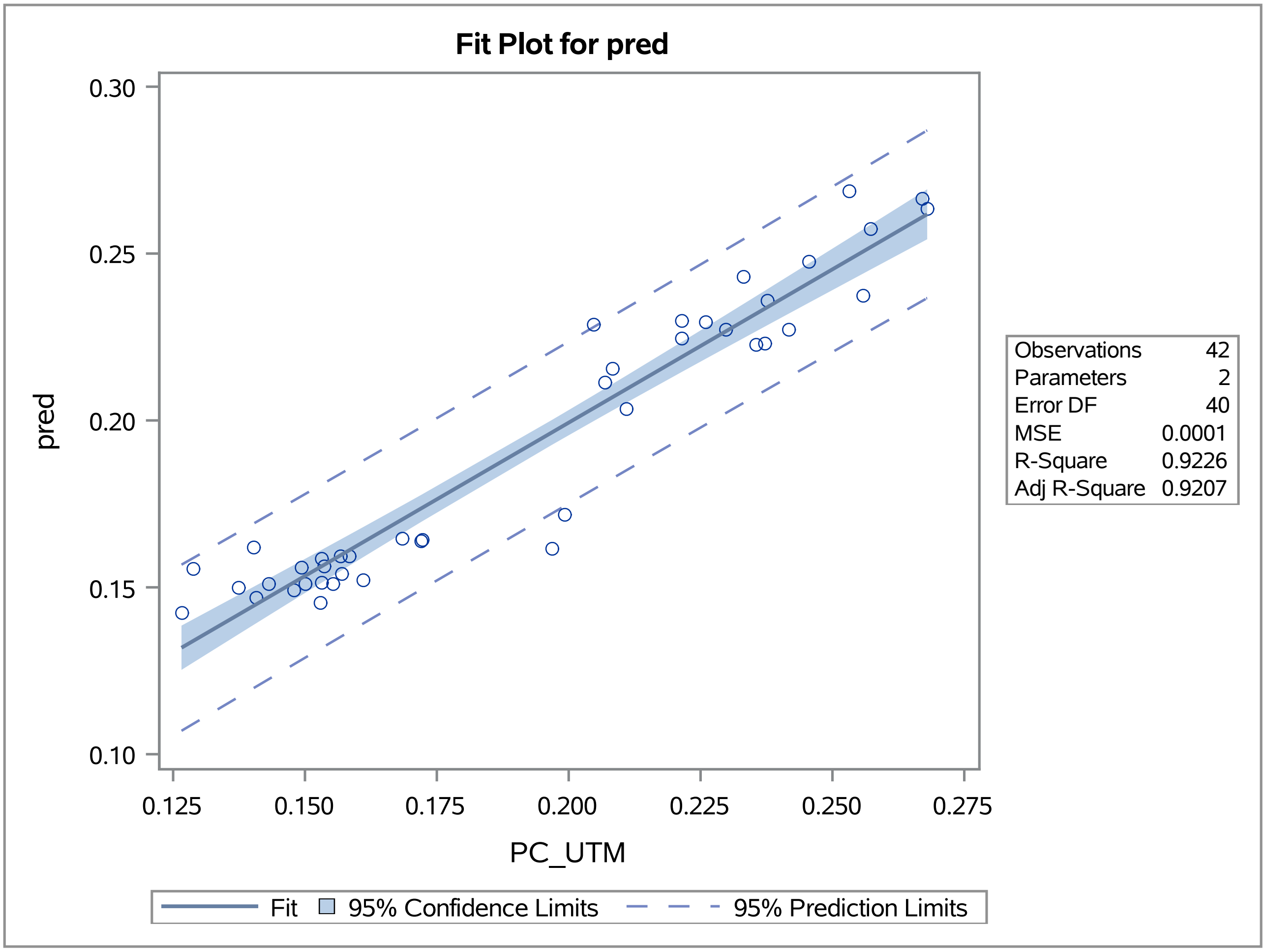

The MEANS Procedure

Analysis Variable : MSE				
N	Mean	Std Dev	Minimum	Maximum
42	0.000146667	0.000254507	1.5479249E-9	0.0012314

The NLIN Procedure
Dependent Variable ERDIA
Method: Gauss-Newton

Iterative Phase			
Iter	a	b	Sum of Squares
0	0.0600	1.0900	9.6828
1	0.0635	0.6848	1.9067
2	0.0624	0.7231	1.6852
3	0.0624	0.7241	1.6852
4	0.0624	0.7241	1.6852

Note: Missing values were generated as a result of performing an operation on missing values. Each place is given by (number of times) AT (statement)/(line):(column).

**6 AT**	1/186:20

NOTE: Convergence criterion met.

Estimation Summary	
**Method**	Gauss-Newton
**Iterations**	4
**R**	3.198E-7
**PPC(b)**	6.608E-8
**RPC(b)**	9.204E-6
**Object**	1.974E-9
**Objective**	1.685157
**Observations Read**	18
**Observations Used**	12
**Observations Missing**	6

Note: An intercept was not specified for this model.

Source	DF	Sum of Squares	Mean Square	F Value	Approx Pr > F
**Model**	2	252.5	126.3	749.33	<.0001
**Error**	10	1.6852	0.1685		
**Uncorrected Total**	12	254.2			

Parameter	Estimate	Approx Std Error	Approximate 95% Confidence Limits	
**a**	0.0624	0.00161	0.0588	0.0660
**b**	0.7241	0.0473	0.6187	0.8295

The NLIN Procedure

Approximate Correlation Matrix		
	a	b
a	1.0000000	0.0605022
b	0.0605022	1.0000000

The REG Procedure
Model: MODEL1
Dependent Variable: pred

Analysis of Variance					
**Source**	**DF**	**Sum of Squares**	**Mean Square**	**F Value**	**Pr > F**
**Model**	1	68.38751	68.38751	415.50	<.0001
**Error**	10	1.64591	0.16459		
**Corrected Total**	11	70.03342			

**Root MSE**	0.40570	**R-Square**	0.9765
**Dependent Mean**	3.89992	**Adj R-Sq**	0.9741
**Coeff Var**	10.40271		

Parameter Estimates						
**Variable**	**Label**	**DF**	**Parameter Estimate**	**Standard Error**	**t Value**	**Pr > |t|**
**Intercept**	Intercept	1	0.09109	0.22052	0.41	0.6883
**ERDIA**	ERDIA	1	0.97660	0.04791	20.38	<.0001

The REG Procedure
Model: MODEL1
Dependent Variable: pred

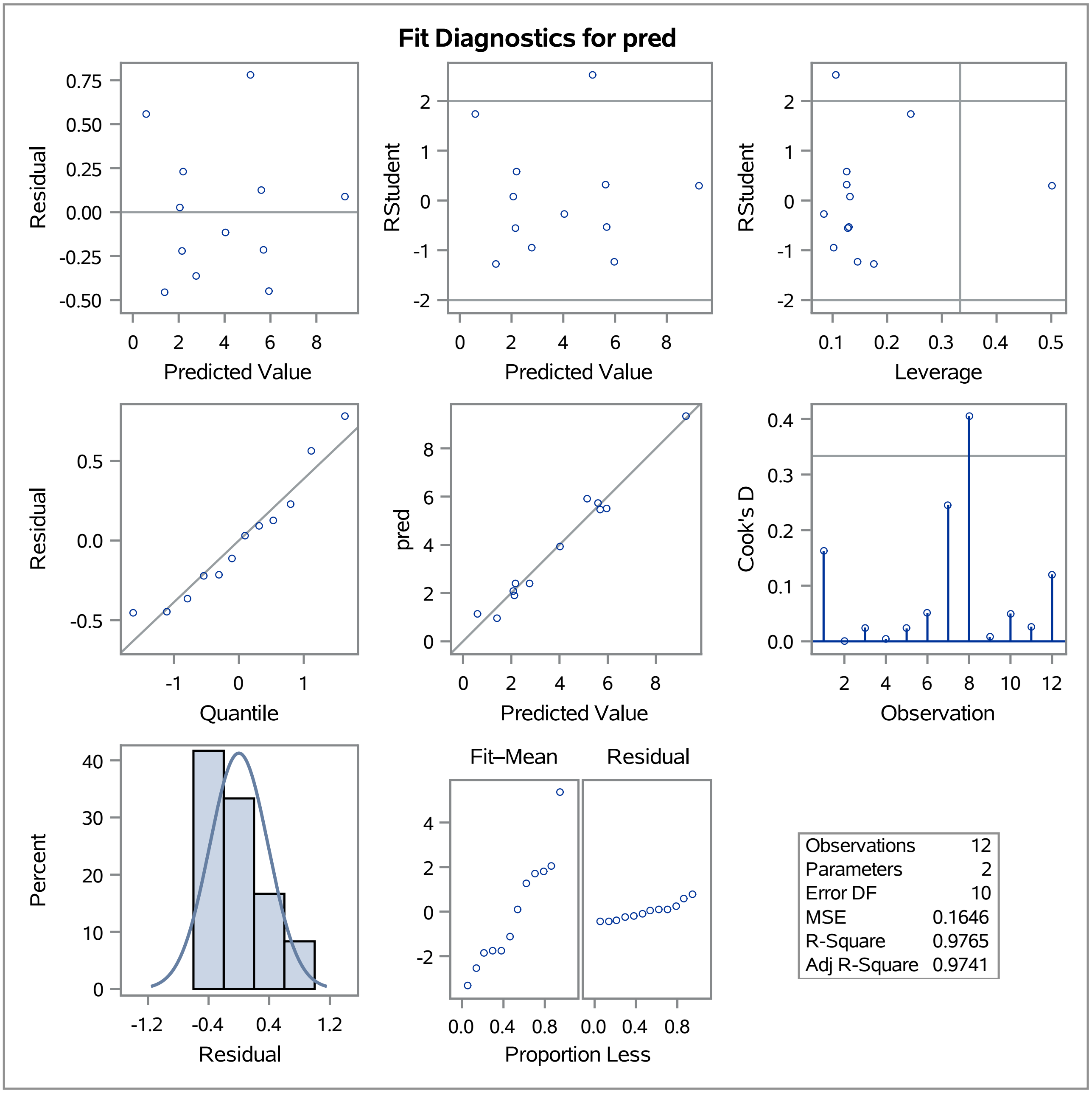

The REG Procedure
Model: MODEL1
Dependent Variable: pred

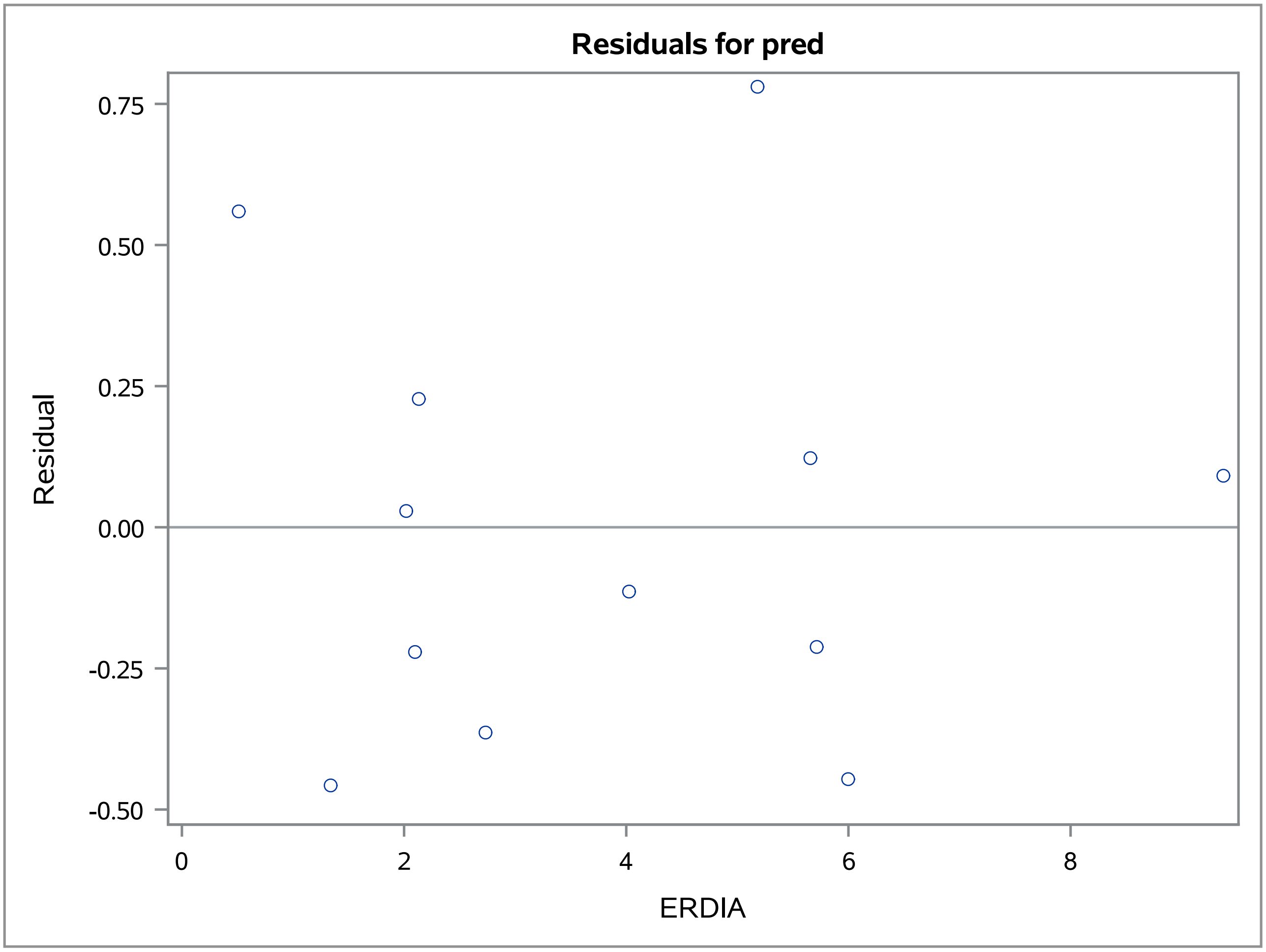

The REG Procedure
Model: MODEL1
Dependent Variable: pred

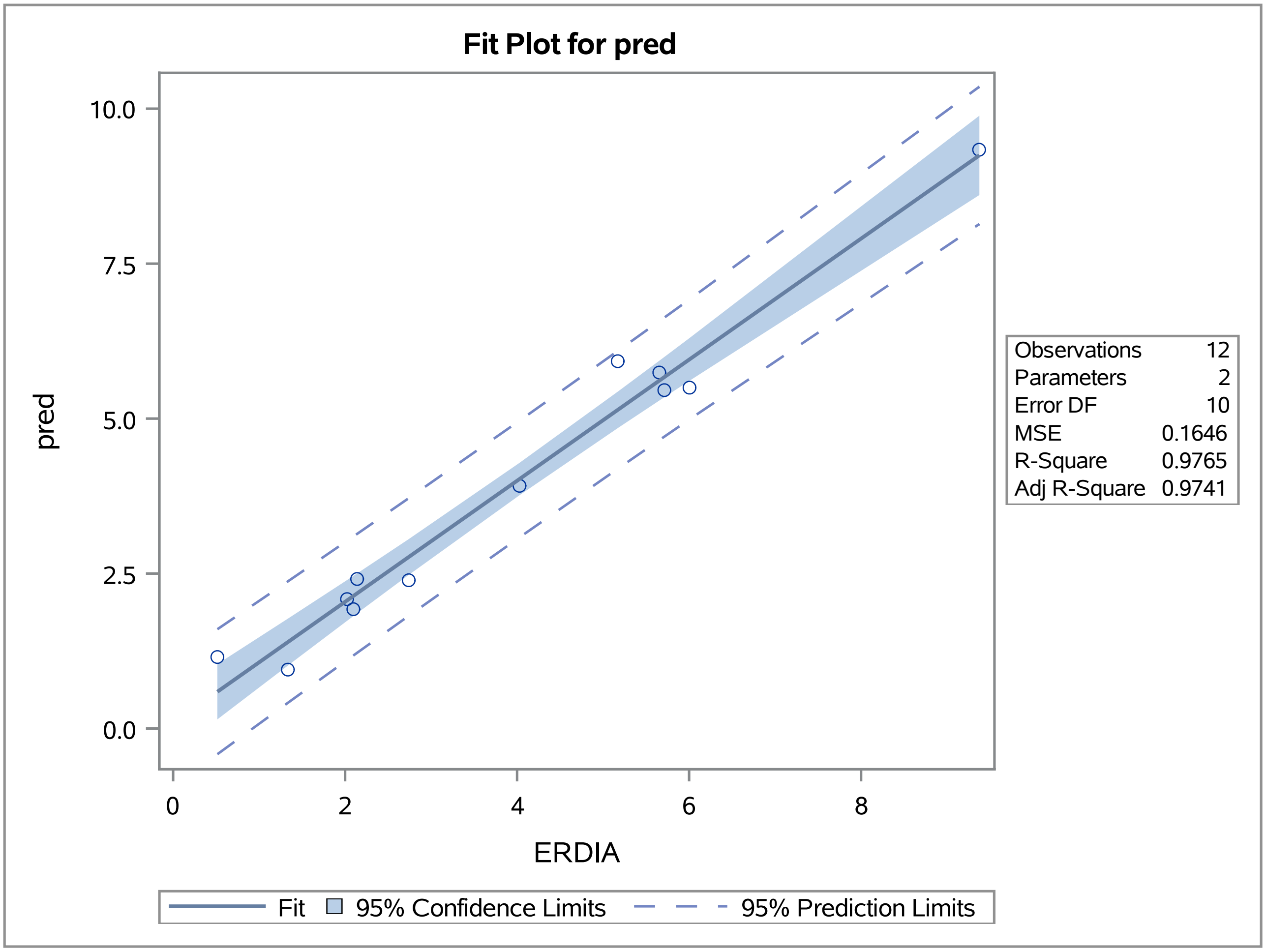

The MEANS Procedure

Analysis Variable : MSE				
N	Mean	Std Dev	Minimum	Maximum
12	0.1404297	0.1800978	0.0014393	0.5631554
