# Supplementary material for: Energy requirements for pregnant dairy cows
Source: PLoS One. 2020 Jul 7;15(7):e0235619. doi: 10.1371/journal.pone.0235619 (PMC7340303; doi:10.1371/journal.pone.0235619)
Supplement: S5 File — (PDF) [file pone.0235619.s005.pdf]

The Mixed Procedure

Model Information	
**Data Set**	WORK.IMPORT
**Dependent Variable**	SALDO
**Covariance Structure**	Diagonal
**Estimation Method**	REML
**Residual Variance Method**	Profile
**Fixed Effects SE Method**	Model-Based
**Degrees of Freedom Method**	Residual

Class Level Information		
**Class**	**Levels**	**Values**
**DIETA**	2	1 2

Dimensions	
**Covariance Parameters**	1
**Columns in X**	9
**Columns in Z**	0
**Subjects**	1
**Max Obs per Subject**	40

Number of Observations	
**Number of Observations Read**	67
**Number of Observations Used**	40
**Number of Observations Not Used**	27

Covariance Parameter Estimates				
**Cov Parm**	**Estimate**	**Standard Error**	**Z Value**	**Pr > Z**
**Residual**	104.61	25.3717	4.12	<.0001

Fit Statistics	
**-2 Res Log Likelihood**	317.9
**AIC (Smaller is Better)**	319.9
**AICC (Smaller is Better)**	320.0
**BIC (Smaller is Better)**	321.4

The Mixed Procedure

Type 3 Tests of Fixed Effects				
Effect	Num DF	Den DF	F Value	Pr > F
DIETA	1	34	0.86	0.3607
GT	1	34	1.93	0.1734
GT*DIETA	1	34	1.03	0.3165
GT*GT	1	34	6.85	0.0131
GT*GT*DIETA	1	34	1.46	0.2345

The NLIN Procedure
Dependent Variable SALDO
Method: Gauss-Newton

Iterative Phase			
**Iter**	**a**	**b**	**Sum of Squares**
**0**	1.7000	0.00530	60774.3
**1**	1.0047	0.00870	54898.0
**2**	0.4291	0.0161	24971.5
**3**	1.2068	0.0124	21757.8
**4**	1.3037	0.0155	10015.1
**5**	1.4500	0.0143	5477.8
**6**	1.4949	0.0141	5452.1
**7**	1.4927	0.0141	5452.1
**8**	1.4928	0.0141	5452.1

Note: Missing values were generated as a result of performing an operation on missing values. Each place is given by (number of times) AT (statement)/(line):(column).

**13 AT**	1/82:16

NOTE: Convergence criterion met.

The NLIN Procedure
Dependent Variable SALDO
Method: Gauss-Newton

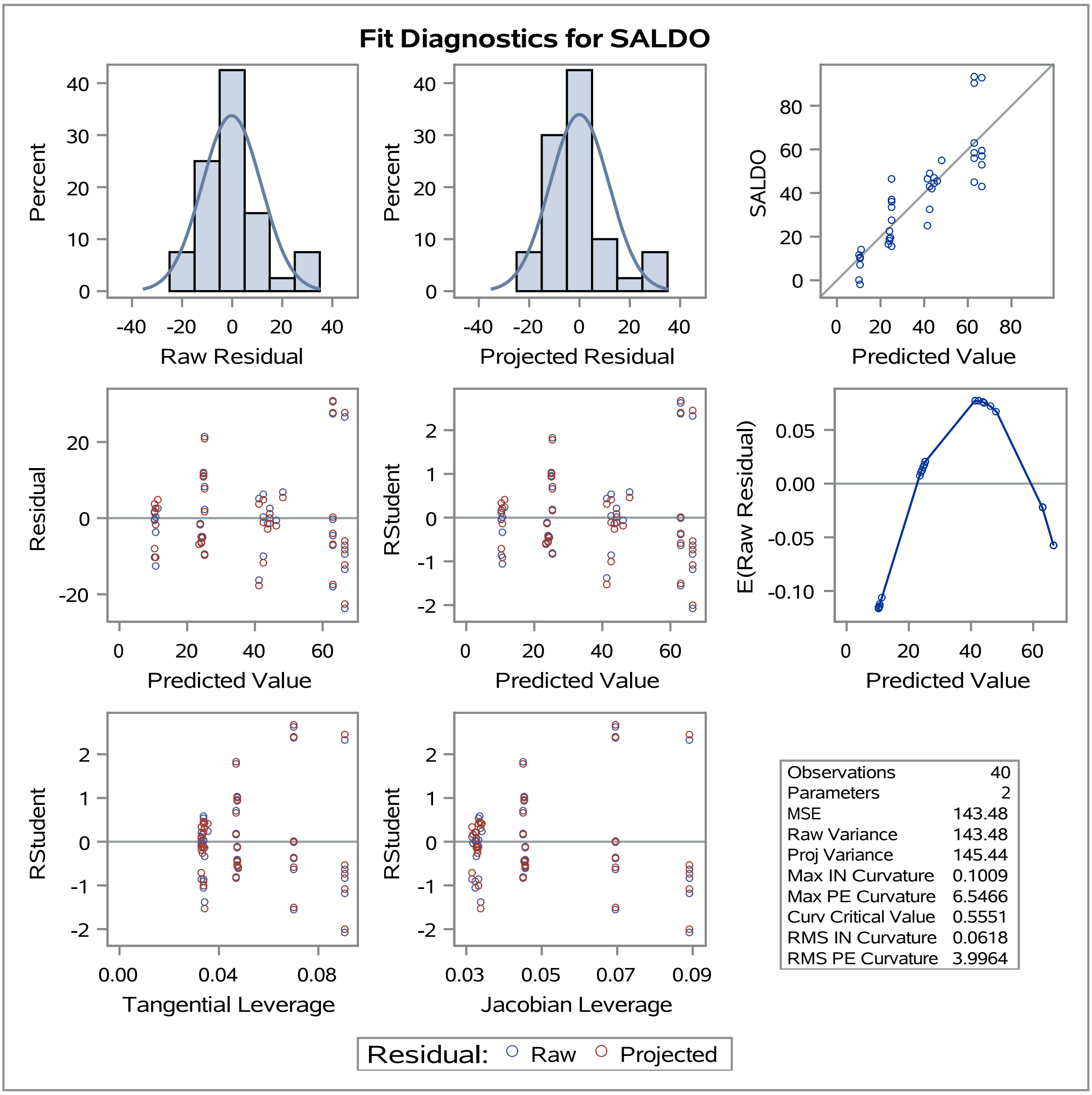

Observations	40
Parameters	2
MSE	143.48
Raw Variance	143.48
Proj Variance	145.44
Max IN Curvature	0.1009
Max PE Curvature	6.5466
Curv Critical Value	0.5551
RMS IN Curvature	0.0618
RMS PE Curvature	3.9964

The NLIN Procedure
Dependent Variable SALDO
Method: Gauss-Newton

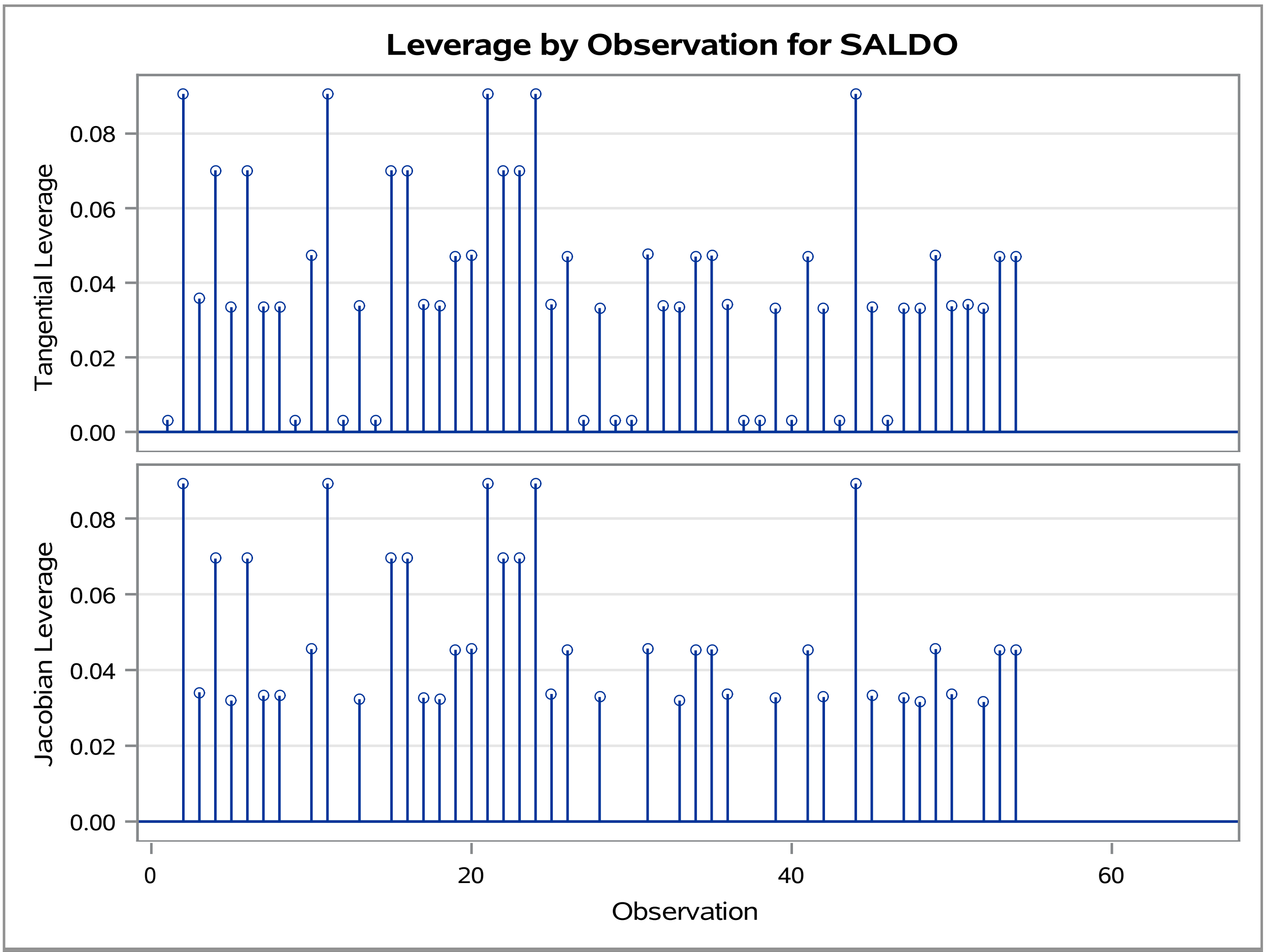

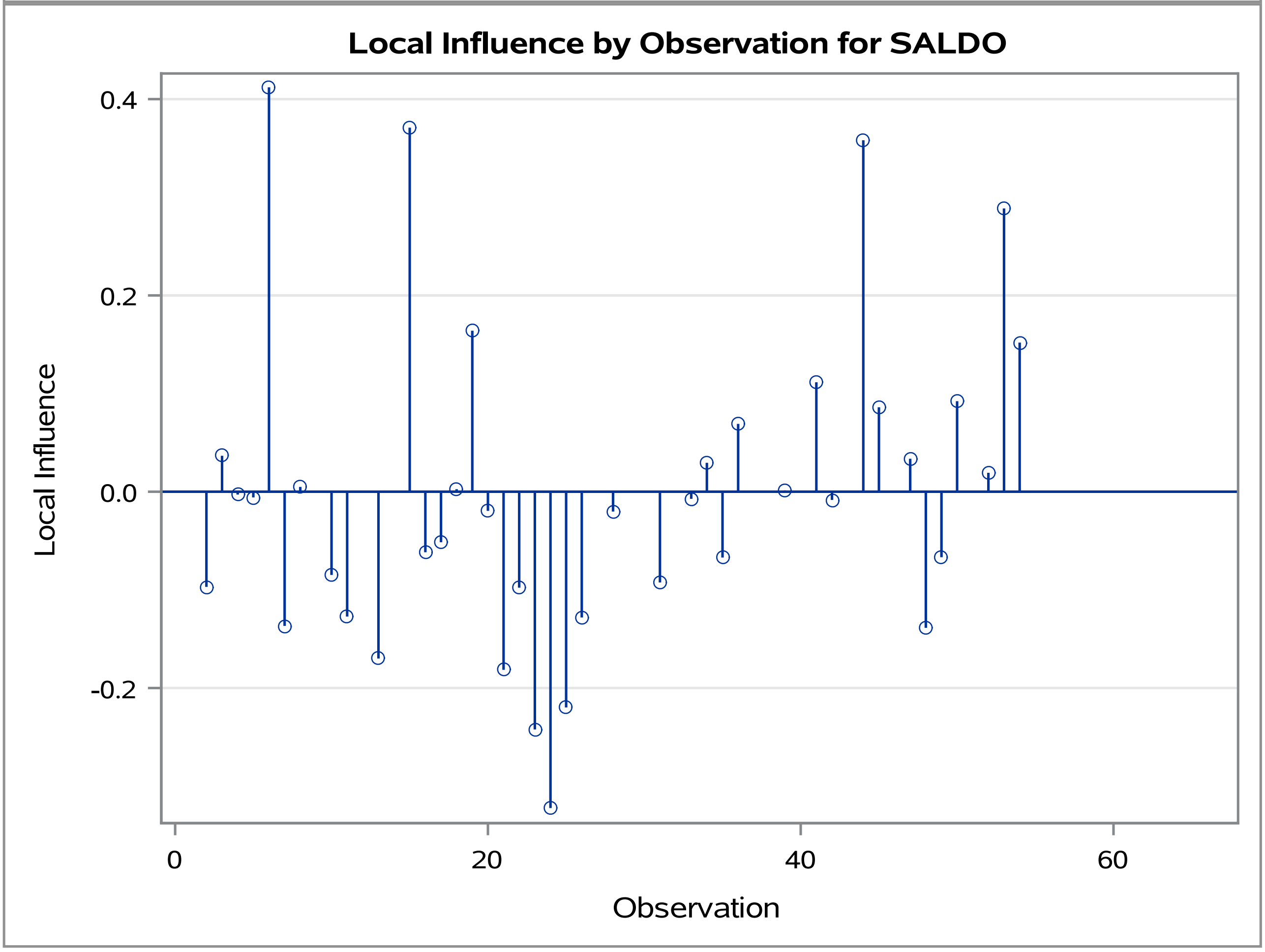

The NLIN Procedure

Estimation Summary	
Method	Gauss-Newton
Iterations	8
Subiterations	4
Average Subiterations	0.5
R	1.585E-6
PPC(a)	4.43E-6
RPC(a)	0.00009
Object	9.67E-10
Objective	5452.056
Observations Read	67
Observations Used	40
Observations Missing	27

Note: An intercept was not specified for this model.

Source	DF	Sum of Squares	Mean Square	F Value	Approx Pr > F
Model	2	72547.4	36273.7	252.82	<.0001
Error	38	5452.1	143.5		
Uncorrected Total	40	77999.4			

Parameter	Estimate	Approx Std Error	Approximate 95% Confidence Limits	
a	1.4928	0.6805	0.1152	2.8704
b	0.0141	0.00179	0.0104	0.0177

Approximate Correlation Matrix		
	a	b
a	1.0000000	-0.9952300
b	-0.9952300	1.0000000

The NLIN Procedure
Dependent Variable SALDO
Method: Gauss-Newton

Iterative Phase			
Iter	a	b	Sum of Squares
0	1.7000	0.00530	28245.4
1	1.7816	0.00490	6587.4
2	1.8849	0.00473	5678.3
3	1.8799	0.00472	5674.4
4	1.8792	0.00472	5674.4
5	1.8793	0.00472	5674.4

Note: Missing values were generated as a result of performing an operation on missing values. Each place is given by (number of times) AT (statement)/(line):(column).

13 AT	1/88:16

NOTE: Convergence criterion met.

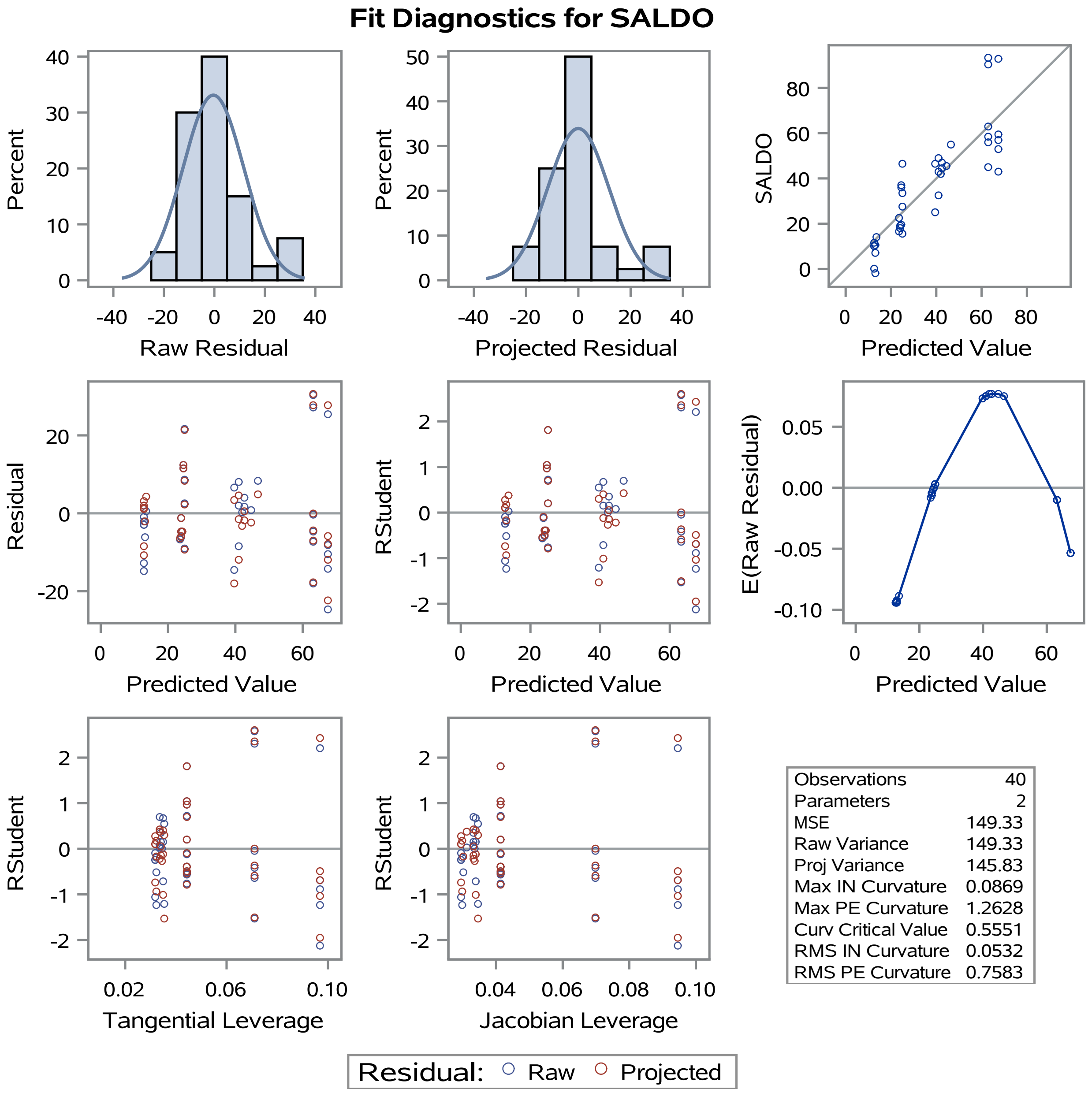

Observations	40
Parameters	2
MSE	149.33
Raw Variance	149.33
Proj Variance	145.83
Max IN Curvature	0.0869
Max PE Curvature	1.2628
Curv Critical Value	0.5551
RMS IN Curvature	0.0532
RMS PE Curvature	0.7583

The NLIN Procedure
Dependent Variable SALDO
Method: Gauss-Newton

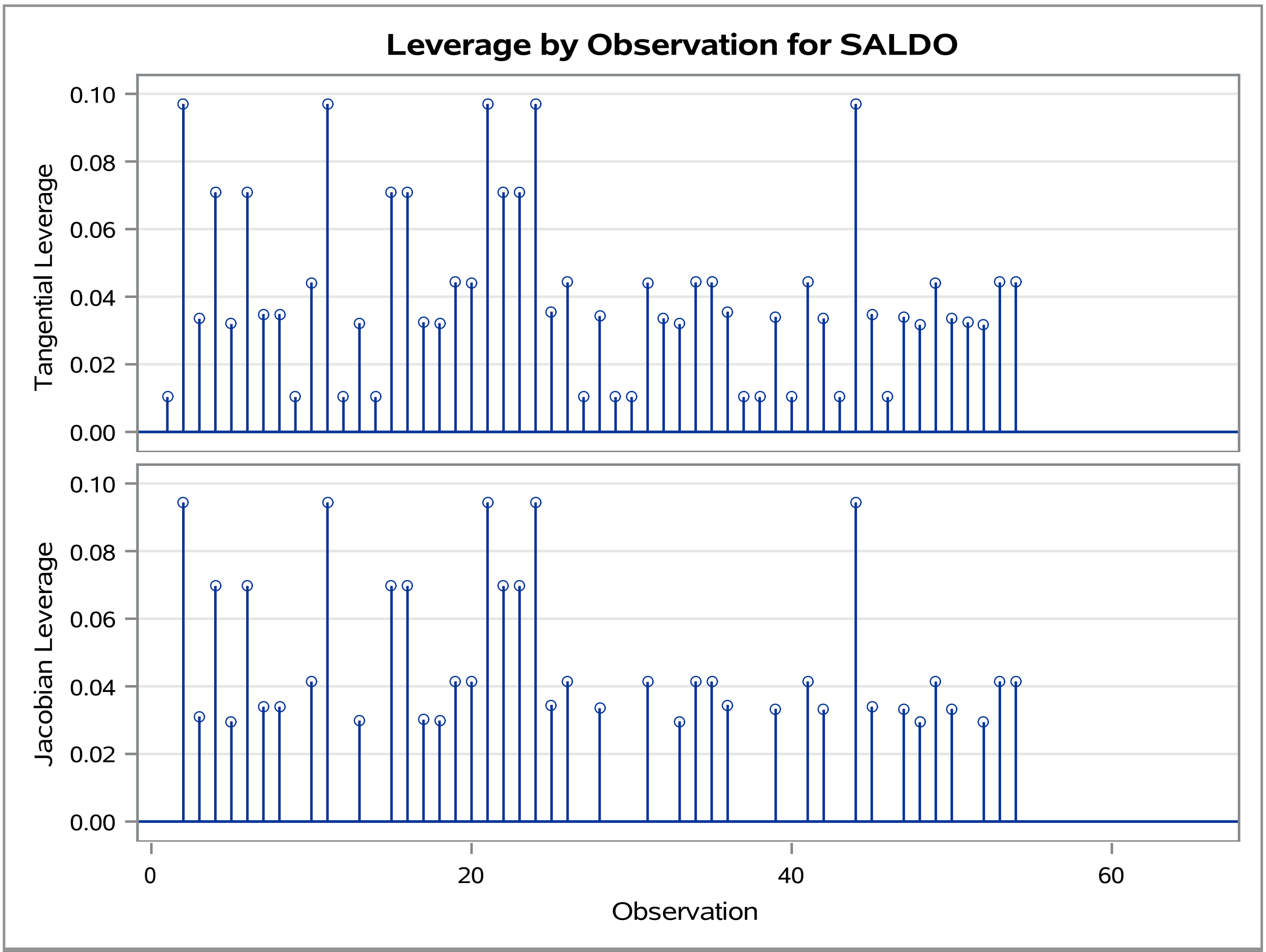

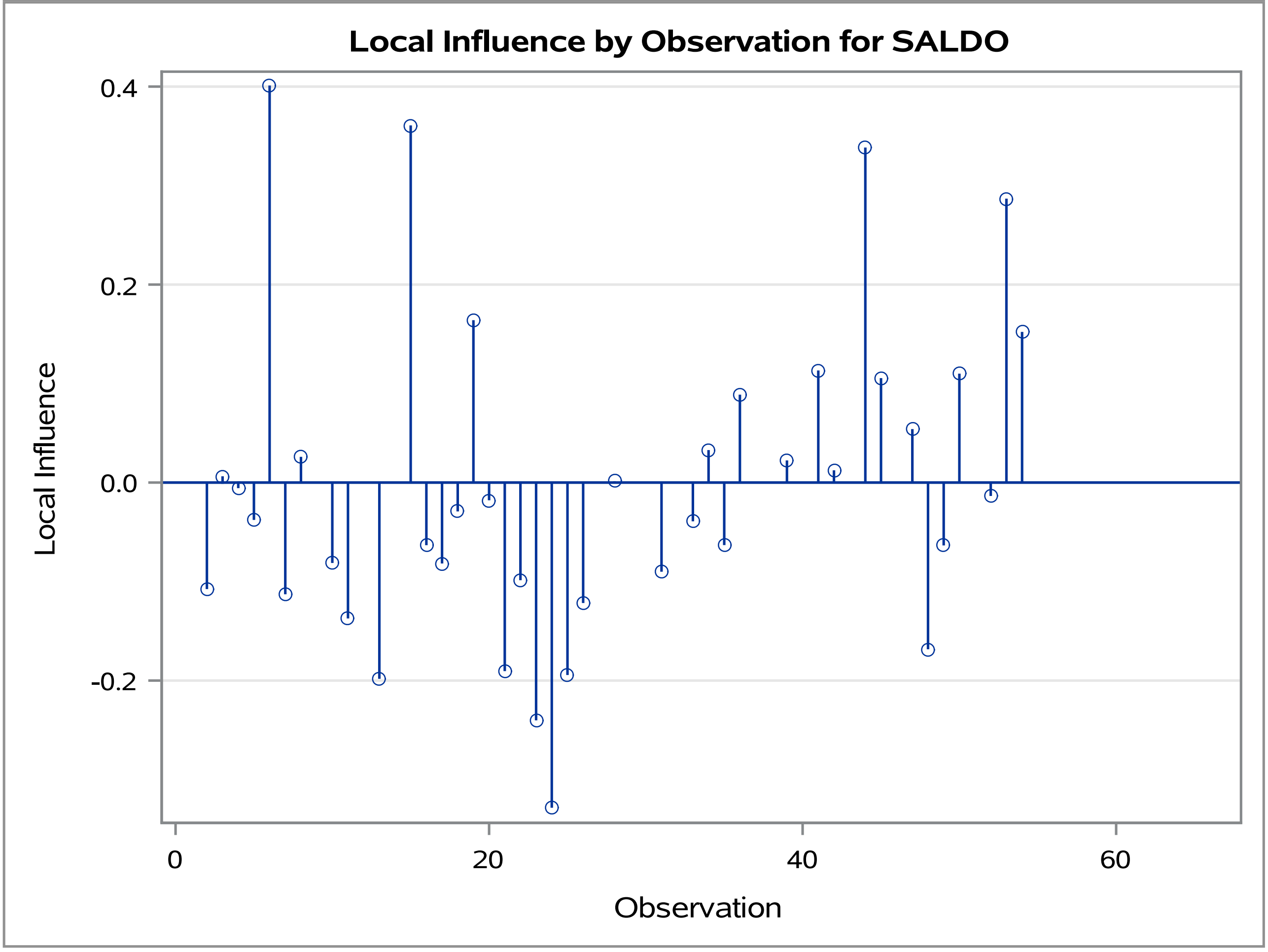

The NLIN Procedure

Estimation Summary	
**Method**	Gauss-Newton
**Iterations**	5
**R**	1.675E-6
**PPC(a)**	2.466E-6
**RPC(a)**	0.000029
**Object**	3.65E-10
**Objective**	5674.369
**Observations Read**	67
**Observations Used**	40
**Observations Missing**	27

Note: An intercept was not specified for this model.

Source	DF	Sum of Squares	Mean Square	F Value	Approx Pr > F
**Model**	2	72325.1	36162.5	242.17	<.0001
**Error**	38	5674.4	149.3		
**Uncorrected Total**	40	77999.4			

Parameter	Estimate	Approx Std Error	Approximate 95% Confidence Limits	
**a**	1.8793	0.4569	0.9543	2.8043
**b**	0.00472	0.000282	0.00415	0.00529

Approximate Correlation Matrix		
	**a**	**b**
**a**	1.0000000	-0.9823820
**b**	-0.9823820	1.0000000

exp x exp testando ad libitum vs mantenca EXP SIMPLES

The NLMIXED Procedure

Specifications	
**Data Set**	WORK.IMPORT
**Dependent Variable**	SALDO
**Distribution for Dependent Variable**	Normal
**Optimization Technique**	Dual Quasi-Newton
**Integration Method**	None

Dimensions	
**Observations Used**	40
**Observations Not Used**	27
**Total Observations**	67
**Parameters**	5

Initial Parameters					
**a1**	**b1**	**a2**	**b2**	**S2**	**Negative Log Likelihood**
1.05	0.016	1.05	0.016	363	164.646923

Iteration History					
**Iteration**	**Calls**	**Negative Log Likelihood**	**Difference**	**Maximum Gradient**	**Slope**
**1**	10	160.1096	4.537315	1776.84	-954518
**2**	17	160.1096	0.000032	1777.61	-4.59836
**3**	21	159.7874	0.322177	621.685	-0.46863
**4**	23	159.7478	0.039574	114.400	-0.09117
**5**	27	159.7451	0.002748	90.3173	-0.00334
**6**	31	159.7266	0.018495	298.906	-0.00271
**7**	35	159.6931	0.033457	626.081	-0.02917
**8**	38	159.6739	0.019256	107.011	-0.04823
**9**	41	159.6612	0.01268	177.235	-0.00959
**10**	44	159.6560	0.005193	60.3519	-0.00586
**11**	47	159.6555	0.000542	11.5033	-0.00075
**12**	50	159.6554	0.000067	7.35558	-0.00009
**13**	52	159.6553	0.000062	21.7703	-0.00008
**14**	56	159.6549	0.000456	44.6730	-0.00011
**15**	60	159.6484	0.006515	65.2169	-0.00077
**16**	63	159.6473	0.001062	41.1439	-0.00246
**17**	66	159.6468	0.000516	14.2958	-0.00087
**18**	69	159.6467	0.000087	7.00141	-0.00011

exp x exp testando ad libitum vs mantenca EXP SIMPLES

The NLMIXED Procedure

Iteration History					
Iteration	Calls	Negative Log Likelihood	Difference	Maximum Gradient	Slope
19	73	159.6465	0.00022	20.0753	-0.00014
20	77	159.6445	0.00197	107.248	-0.00028
21	81	159.6339	0.010599	269.561	-0.00297
22	85	159.6010	0.032899	283.581	-0.06503
23	92	159.4020	0.199022	1236.86	-0.04524
24	99	158.5751	0.826831	2402.40	-0.43373
25	101	157.3129	1.262245	3073.78	-7.73699
26	103	153.5467	3.766167	2018.95	-53.8912
27	109	152.5980	0.948749	1476.43	-11.7065
28	112	152.4899	0.108085	628.079	-0.33999
29	115	152.4353	0.054611	632.528	-0.05951
30	119	151.8816	0.553738	2094.06	-0.14397
31	123	151.2319	0.649668	5421.13	-1.57961
32	126	150.8868	0.345057	4255.88	-3.59371
33	128	150.3388	0.54801	2028.89	-3.71084
34	130	149.5470	0.791836	3094.27	-0.96029
35	132	148.8402	0.706789	2460.95	-1.91024
36	137	148.5301	0.310065	966.637	-2.51636
37	139	148.3410	0.189084	1968.20	-0.57389
38	142	148.2629	0.078113	1010.41	-0.29450
39	146	148.0631	0.199843	240.578	-0.29106
40	148	147.9444	0.118719	2943.91	-0.18751
41	152	147.6637	0.280648	340.418	-0.83106
42	154	147.3784	0.285335	2943.41	-0.24647
43	157	147.2991	0.079246	1071.62	-0.37998
44	159	147.2228	0.076313	497.282	-0.13198
45	161	147.1710	0.051792	1998.55	-0.12693
46	163	147.1106	0.06043	475.827	-0.17354
47	165	147.0513	0.059335	153.082	-0.03031
48	168	147.0386	0.012709	298.968	-0.02697
49	171	147.0336	0.004966	471.129	-0.01145
50	175	147.0135	0.020122	864.457	-0.01146
51	178	147.0029	0.010555	560.892	-0.03109
52	180	146.9922	0.010764	387.043	-0.01578
53	182	146.9731	0.019068	214.969	-0.02076
54	185	146.9674	0.005641	42.7898	-0.01762
55	188	146.9656	0.00186	99.3692	-0.00362

exp x exp testando ad libitum vs mantenca EXP SIMPLES

The NLMIXED Procedure

Iteration History					
Iteration	Calls	Negative Log Likelihood	Difference	Maximum Gradient	Slope
**56**	191	146.9649	0.000637	39.4110	-0.00057
**57**	194	146.9649	0.000091	2.90577	-0.00018
**58**	197	146.9649	3.97E-6	3.69359	-7.1E-6
**59**	200	146.9649	4.034E-7	0.38249	-6.61E-7

NOTE: GCONV convergence criterion satisfied.

Fit Statistics	
**-2 Log Likelihood**	293.9
**AIC (smaller is better)**	303.9
**AICC (smaller is better)**	305.7
**BIC (smaller is better)**	312.4

Parameter Estimates								
Parameter	Estimate	Standard Error	DF	t Value	Pr > |t|	95% Confidence Limits		Gradient
**a1**	1.4697	0.7837	40	1.88	0.0681	-0.1142	3.0536	0.000572
**b1**	0.01353	0.002096	40	6.46	<.0001	0.009298	0.01777	0.22952
**a2**	1.4150	0.6643	40	2.13	0.0394	0.07236	2.7577	0.001028
**b2**	0.01492	0.001845	40	8.09	<.0001	0.01119	0.01865	0.38249
**S2**	90.9540	20.3442	40	4.47	<.0001	49.8368	132.07	-2.69E-6

Correlation Matrix of Parameter Estimates					
	a1	b1	a2	b2	S2
**a1**	1.0000	-0.9947	0.0003	-0.0003	-0.0152
**b1**	-0.9947	1.0000	-0.0003	0.0003	0.0153
**a2**	0.0003	-0.0003	1.0000	-0.9953	-0.0198
**b2**	-0.0003	0.0003	-0.9953	1.0000	0.0198
**S2**	-0.0152	0.0153	-0.0198	0.0198	1.0000

Additional Estimates								
Label	Estimate	Standard Error	DF	t Value	Pr > |t|	Alpha	Lower	Upper
**La1-Ma2**	0.05468	1.0272	40	0.05	0.9578	0.05	-2.0214	2.1308
**Lb1-Mb2**	-0.00139	0.002792	40	-0.50	0.6216	0.05	-0.00703	0.004253

exp x exp testando ad libitum vs mantenca EXP SIMPLES

The NLMIXED Procedure

Correlation Matrix of Additional Estimates		
**Label**	**Corr1**	**Corr2**
**La1-Ma2**	1.0000	-0.9948
**Lb1-Mb2**	-0.9948	1.0000

exp x exp testando ad libitum vs mantenca DUPLO EXP

The NLMIXED Procedure

Specifications	
**Data Set**	WORK.IMPORT
**Dependent Variable**	SALDO
**Distribution for Dependent Variable**	Normal
**Optimization Technique**	Dual Quasi-Newton
**Integration Method**	None

Dimensions	
**Observations Used**	40
**Observations Not Used**	27
**Total Observations**	67
**Parameters**	5

Initial Parameters					
a1	b1	a2	b2	S2	Negative Log Likelihood
1.9303	0.0051	1.9303	0.0051	363	182.349106

Iteration History					
Iteration	Calls	Negative Log Likelihood	Difference	Maximum Gradient	Slope
**1**	20	166.1354	16.21375	13294.6	-9.516E7
**2**	29	165.7967	0.338681	13153.2	-497212
**3**	33	162.0759	3.720801	15517.8	-2.59522
**4**	35	160.4310	1.644859	6389.59	-2.56219
**5**	37	160.2078	0.223242	6708.00	-1.43400
**6**	39	159.9746	0.233195	967.693	-0.56399
**7**	42	159.9694	0.005217	131.763	-0.00948
**8**	45	159.9682	0.001188	191.773	-0.00112
**9**	49	159.9477	0.020457	877.473	-0.00183
**10**	52	159.9335	0.014237	394.515	-0.02462
**11**	55	159.9316	0.001899	32.7448	-0.00376
**12**	58	159.9315	0.000037	16.7677	-0.00005
**13**	64	159.9289	0.002691	566.044	-0.00003
**14**	68	159.9207	0.008143	106.561	-0.00397
**15**	71	159.9202	0.000499	122.241	-0.00051
**16**	75	159.9147	0.00549	416.512	-0.00046
**17**	81	159.7048	0.209954	2973.58	-0.01178
**18**	85	158.4640	1.240736	4629.57	-1.03360

exp x exp testando ad libitum vs mantenca DUPLO EXP

The NLMIXED Procedure

Iteration History					
**Iteration**	**Calls**	**Negative Log Likelihood**	**Difference**	**Maximum Gradient**	**Slope**
**19**	92	154.5438	3.92025	8229.04	-2.63238
**20**	98	153.7036	0.840204	7786.08	-5.85993
**21**	100	153.0672	0.636379	22865.5	-4.01095
**22**	104	151.4409	1.626317	23170.8	-5.57651
**23**	106	150.1386	1.302262	27451.2	-10.3811
**24**	108	148.7337	1.404901	5631.92	-4.19328
**25**	113	148.5867	0.147037	887.456	-0.78659
**26**	117	148.1426	0.444099	2742.53	-1.54243
**27**	120	148.0620	0.080555	344.610	-0.24130
**28**	123	148.0416	0.020455	396.578	-0.05593
**29**	126	148.0391	0.002449	139.901	-0.00529
**30**	129	148.0391	0.000054	18.7339	-0.00012
**31**	132	148.0391	1.333E-6	1.61439	-2.78E-6
**32**	135	148.0391	9.329E-9	0.10833	-1.77E-8

NOTE: GCONV convergence criterion satisfied.

Fit Statistics	
**-2 Log Likelihood**	296.1
**AIC (smaller is better)**	306.1
**AICC (smaller is better)**	307.8
**BIC (smaller is better)**	314.5

Parameter Estimates								
**Parameter**	**Estimate**	**Standard Error**	**DF**	**t Value**	**Pr > |t|**	**95% Confidence Limits**		**Gradient**
**a1**	1.7416	0.4869	40	3.58	0.0009	0.7575	2.7257	-0.00008
**b1**	0.004636	0.000329	40	14.08	<.0001	0.003970	0.005301	-0.10833
**a2**	1.9888	0.4751	40	4.19	0.0002	1.0287	2.9490	-0.00002
**b2**	0.004850	0.000265	40	18.27	<.0001	0.004313	0.005386	-0.03948
**S2**	95.9735	21.4792	40	4.47	<.0001	52.5625	139.38	-9.7E-7

exp x exp testando ad libitum vs mantenca DUPLO EXP

The NLMIXED Procedure

Correlation Matrix of Parameter Estimates					
	a1	b1	a2	b2	S2
a1	1.0000	-0.9792	0.0008	-0.0008	-0.0245
b1	-0.9792	1.0000	-0.0008	0.0008	0.0250
a2	0.0008	-0.0008	1.0000	-0.9808	-0.0329
b2	-0.0008	0.0008	-0.9808	1.0000	0.0335
S2	-0.0245	0.0250	-0.0329	0.0335	1.0000

Additional Estimates								
Label	Estimate	Standard Error	DF	t Value	Pr > |t|	Alpha	Lower	Upper
La1-Ma2	-0.2472	0.6800	40	-0.36	0.7181	0.05	-1.6216	1.1271
Lb1-Mb2	-0.00021	0.000423	40	-0.51	0.6151	0.05	-0.00107	0.000640

Correlation Matrix of Additional Estimates		
Label	Corr1	Corr2
La1-Ma2	1.0000	-0.9756
Lb1-Mb2	-0.9756	1.0000

EXP SIMPLES modelo reduzido

The NLMIXED Procedure

Specifications	
**Data Set**	WORK.IMPORT
**Dependent Variable**	SALDO
**Distribution for Dependent Variable**	Normal
**Optimization Technique**	Dual Quasi-Newton
**Integration Method**	None

Dimensions	
**Observations Used**	40
**Observations Not Used**	27
**Total Observations**	67
**Parameters**	3

Initial Parameters

a	b	S2	Negative Log Likelihood
1.05	0.016	363	164.646923

Iteration History

Iteration	Calls	Negative Log Likelihood	Difference	Maximum Gradient	Slope
**1**	15	162.5699	2.076991	2636.00	-824647
**2**	17	162.2792	0.290714	573.760	-0.46846
**3**	19	162.2621	0.0171	70.2499	-0.03333
**4**	26	162.1904	0.071721	261.689	-0.00370
**5**	28	162.1781	0.012292	589.326	-0.02822
**6**	30	162.1610	0.017142	17.7545	-0.02329
**7**	35	162.1556	0.005386	179.693	-0.00709
**8**	38	162.1544	0.001138	2.27803	-0.00264
**9**	41	162.1543	0.000156	5.91290	-0.00019
**10**	44	162.1543	0.000029	11.6563	-0.00002
**11**	50	162.1519	0.002393	72.9281	-0.00003
**12**	59	161.9923	0.159573	319.323	-0.00580
**13**	61	161.8466	0.145733	2058.84	-0.18168
**14**	65	160.9691	0.877443	4716.47	-2.64664
**15**	67	159.5009	1.468245	70.9242	-6.72422
**16**	73	159.1963	0.304593	439.470	-0.84912
**17**	76	159.1359	0.060376	1734.25	-0.06052
**18**	80	158.5541	0.581806	3453.43	-0.15085

EXP SIMPLES modelo reduzido

The NLMIXED Procedure

Iteration History

Iteration	Calls	Negative Log Likelihood	Difference	Maximum Gradient	Slope
**19**	85	156.9758	1.57833	2660.19	-3.78912
**20**	91	156.6216	0.354133	5229.34	-2.93134
**21**	93	156.4121	0.209522	1460.63	-1.95557
**22**	95	156.0439	0.368178	3456.34	-0.34919
**23**	98	155.8175	0.226399	4027.67	-0.31767
**24**	100	155.4601	0.357467	1126.80	-0.63767
**25**	105	155.2650	0.195085	2136.15	-0.38204
**26**	107	155.1307	0.134279	587.072	-0.26253
**27**	112	155.1115	0.01916	1415.26	-0.15911
**28**	116	155.0615	0.050073	296.482	-0.12318
**29**	119	155.0555	0.005996	124.654	-0.00445
**30**	122	155.0549	0.000543	6.01481	-0.00113
**31**	125	155.0549	0.000011	0.31519	-0.00002
**32**	128	155.0549	1.059E-7	0.52862	-2.67E-7

NOTE: GCONV convergence criterion satisfied.

Fit Statistics

**-2 Log Likelihood**	310.1
**AIC (smaller is better)**	316.1
**AICC (smaller is better)**	316.8
**BIC (smaller is better)**	321.2

Parameter Estimates

Parameter	Estimate	Standard Error	DF	t Value	Pr > |t|	95% Confidence Limits		Gradient
**a**	1.4929	0.6475	40	2.31	0.0264	0.1842	2.8015	0.001495
**b**	0.01406	0.001705	40	8.25	<.0001	0.01062	0.01751	0.52862
**S2**	136.30	30.4847	40	4.47	<.0001	74.6906	197.91	1.093E-6

Correlation Matrix of Parameter Estimates

	a	b	S2
**a**	1.0000	-0.9950	-0.0199
**b**	-0.9950	1.0000	0.0200
**S2**	-0.0199	0.0200	1.0000

EXP SIMPLES modelo reduzido

The REG Procedure
Model: MODEL1
Dependent Variable: Pred Predicted Value

**Number of Observations Read**	67
**Number of Observations Used**	40
**Number of Observations with Missing Values**	27

Analysis of Variance					
Source	DF	Sum of Squares	Mean Square	F Value	Pr > F
**Model**	1	12206	12206	117.37	<.0001
**Error**	38	3951.61647	103.98991		
**Corrected Total**	39	16157			

**Root MSE**	10.19754	**R-Square**	0.7554
**Dependent Mean**	37.54691	**Adj R-Sq**	0.7490
**Coeff Var**	27.15948		

Parameter Estimates						
Variable	Label	DF	Parameter Estimate	Standard Error	t Value	Pr > |t|
**Intercept**	Intercept	1	9.90300	3.01836	3.28	0.0022
**SALDO**	SALDO	1	0.74052	0.06835	10.83	<.0001

EXP SIMPLES modelo reduzido

The REG Procedure
Model: MODEL1
Dependent Variable: Pred Predicted Value

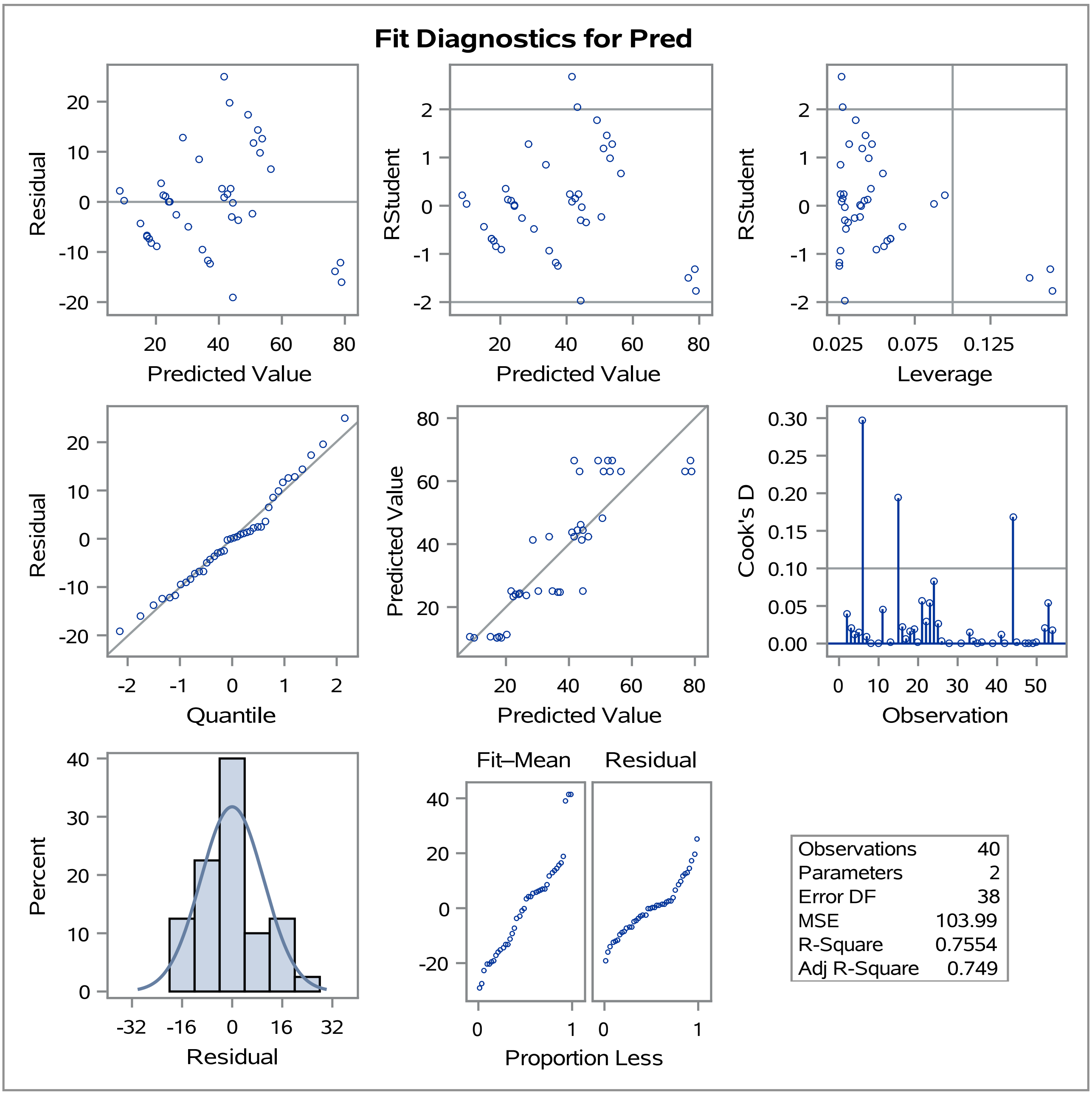

EXP SIMPLES modelo reduzido

The REG Procedure
Model: MODEL1
Dependent Variable: Pred Predicted Value

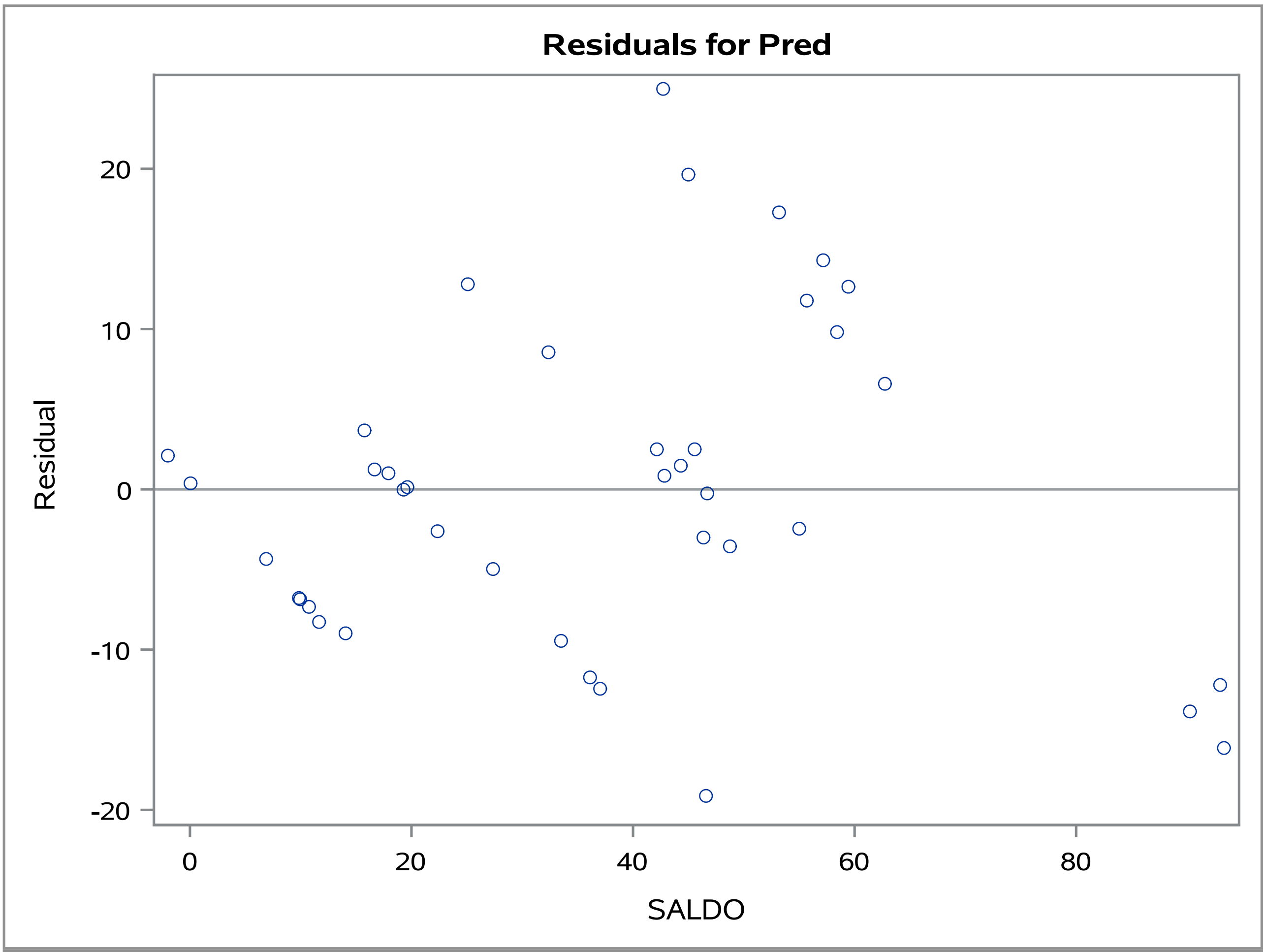

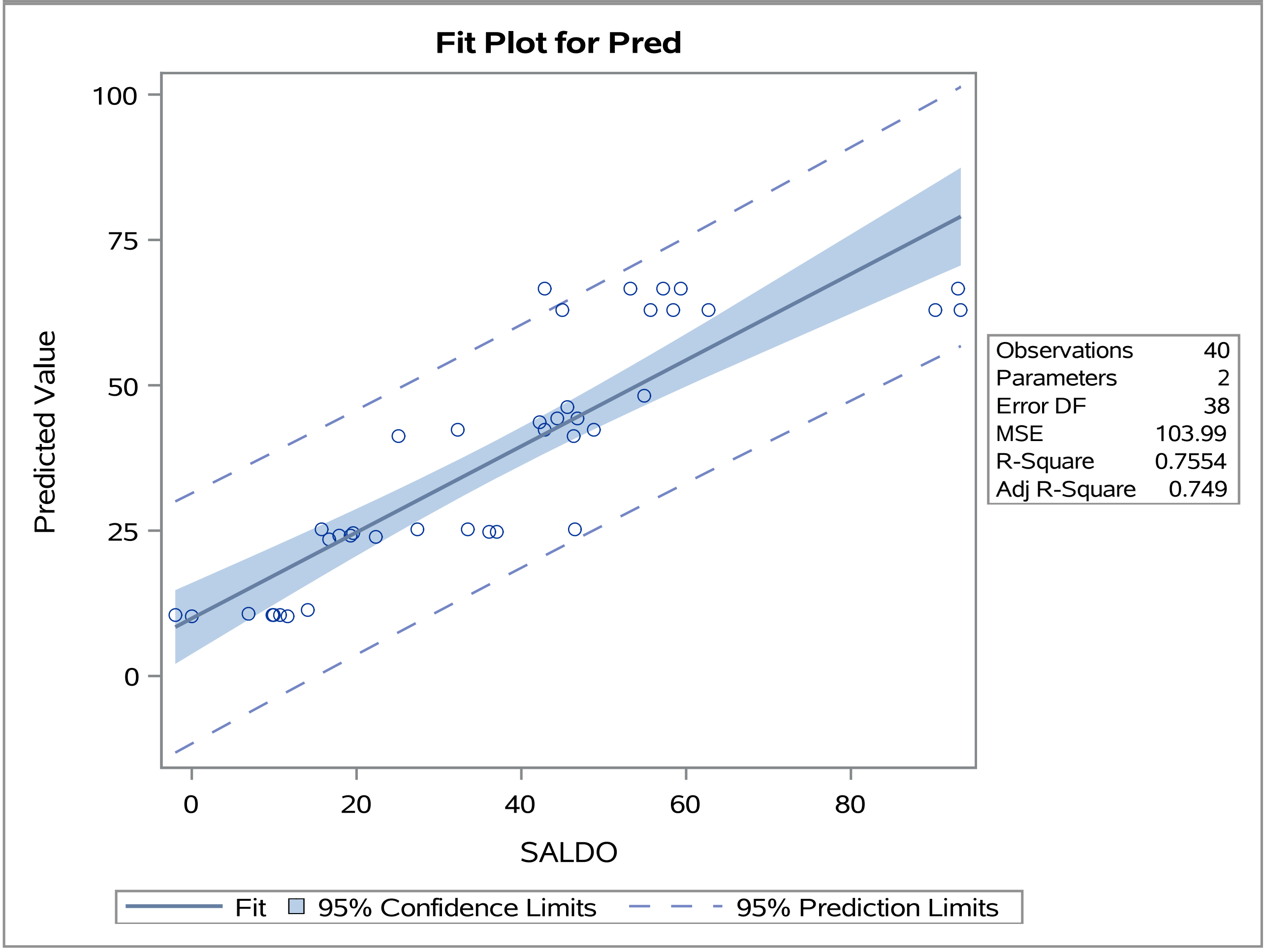

EXP SIMPLES modelo reduzido

The MEANS Procedure

Analysis Variable : MSE				
N	Mean	Std Dev	Minimum	Maximum
40	136.3014097	227.3184195	0.0092131	925.5726751
